# Exploring the Potential Role of Moonlighting Function of the Surface-Associated Proteins From *Mycobacterium bovis* BCG Moreau and Pasteur by Comparative Proteomic

**DOI:** 10.3389/fimmu.2019.00716

**Published:** 2019-04-26

**Authors:** Talita Duarte Pagani, Ana Carolina R. Guimarães, Mariana C. Waghabi, Paloma Rezende Corrêa, Dário Eluan Kalume, Marcia Berrêdo-Pinho, Wim Maurits Degrave, Leila Mendonça-Lima

**Affiliations:** ^1^Laboratório de Genômica Funcional e Bioinformática, Instituto Oswaldo Cruz, Fundação Oswaldo Cruz, Rio de Janeiro, Brazil; ^2^Laboratório Interdisciplinar de Pesquisas Médicas, Instituto Oswaldo Cruz, Fundação Oswaldo Cruz—FIOCRUZ, Rio de Janeiro, Brazil; ^3^Unidade de Espectrometria de Massas e Proteômica, Instituto de Bioquímica Médica Leopoldo de Meis, Universidade Federal do Rio de Janeiro, Rio de Janeiro, Brazil; ^4^Laboratório de Microbiologia Celular, Instituto Oswaldo Cruz, Fundação Oswaldo Cruz, Rio de Janeiro, Brazil

**Keywords:** *Mycobacterium bovis* BCG Moreau, bi-dimensional electrophoresis, MALDI-TOF-TOF, surface-associated proteins, moonlighting proteins

## Abstract

Surface-associated proteins from *Mycobacterium bovis* BCG Moreau RDJ are important components of the live Brazilian vaccine against tuberculosis. They are important targets during initial BCG vaccine stimulation and modulation of the host's immune response, especially in the bacterial-host interaction. These proteins might also be involved in cellular communication, chemical response to the environment, pathogenesis processes through mobility, colonization, and adherence to the host cell, therefore performing multiple functions. In this study, the proteomic profile of the surface-associated proteins from *M. bovis* BCG Moreau was compared to the BCG Pasteur reference strain. The methodology used was 2DE gel electrophoresis combined with mass spectrometry techniques (MALDI-TOF/TOF), leading to the identification of 115 proteins. Of these, 24 proteins showed differential expression between the two BCG strains. Furthermore, 27 proteins previously described as displaying moonlighting function were identified, 8 of these proteins showed variation in abundance comparing BCG Moreau to Pasteur and 2 of them presented two different domain hits. Moonlighting proteins are multifunctional proteins in which two or more biological functions are fulfilled by a single polypeptide chain. Therefore, the identification of such proteins with moonlighting predicted functions can contribute to a better understanding of the molecular mechanisms unleashed by live BCG Moreau RDJ vaccine components.

## Introduction

Tuberculosis (TB) is one of the 10 major causes of death worldwide. According to the World Health Organization (WHO), TB killed 1.7 million people in 2016 with 10.4 million new cases estimated worldwide ratifying the need for more effective treatment and prevention (1). To date, Bacillus Calmette-Guérin (BCG) is the only widely used prophylactic measure against TB (1). BCG is an attenuated *Mycobacterium bovis* strain obtained at the beginning of the Twentieth century at the Pasteur Institute, in Lille. It was distributed to more than 34 countries and maintained in culture without adequate standardization for a long time (2). Because of *in vitro* evolution, slightly different BCG substrains emerged from the parental BCG, such as those with deletions and duplications of genomic regions and/or single nucleotide polymorphism (SNPs) well documented through analysis and genome sequencing (3–9). These genetic differences among the various BCG strains in use worldwide partially explain the variable efficacy in protection against pulmonary TB in adults (2). In Brazil, the strain used for vaccine production since 1927 is *M. bovis* BCG Moreau. The genomic comparison between BCG Pasteur, reference strain, and BCG Moreau showed regions of difference (RD), for example the loss of RD2 and RD14 in BCG Pasteur and RD16 in BCG Moreau, leading to unique genomic/proteomic characteristics (5, 10). These particularities justify more detailed proteomic studies in order to elucidate which proteins are effectively expressed by these bacteria.

BCG is a live attenuated vaccine, and the expression of secreted and surface-associated proteins is extremely relevant since these proteins may play a role in the bacteria-host cell interaction at the beginning of infection (11, 12). These proteins can also be released from the surface since they are non-covalently linked to the mycomembrane (13). Many intracellular proteins with a known function in cell metabolism have also been found on the cell surface, such as glutamine synthetase, gamma-glutamyl phosphate reductase, and cysteine desulfurase (14). Different factors may contribute to the switch between functions such as release to the extracellular space, changes in temperature, redox state of the cell, oligomeric state of the protein, direct interactions with a variety of binding partner proteins, or even to changes in the cellular concentration of a ligand/substrate, cofactor or product, bringing to light the importance of surface-associated proteins playing different roles in cell system (15, 16). This switch between functions is a characteristic called moonlighting (15, 17).

Considering this variability on protein expression, localization and function(s) in different strains of BCG, we compared the surface-associated proteome from the Brazilian strain used in TB vaccine production, *M. bovis* BCG Moreau, to that of BCG Pasteur through 2DE gel electrophoresis combined with mass spectrometry. Complementary to the information already available in the literature, our approach allows a more confident evaluation of expression, abundance, localization and function(s) of proteins between these two BCG strains. The results presented here may lead to the identification of key components of the *M. bovis* BCG Moreau vaccine strain which can be related to the variability in immunological response observed in vaccinated individuals.

## Materials and Methods

### Mycobacterial Cultivation

*Mycobacterium bovis* BCG Pasteur strain 1173P2 was obtained from the Pasteur Institute (Paris, France) and seed-stocks maintained at −80°C. *M. bovis* BCG Moreau was supplied in Sauton/potato medium by the Ataulpho de Paiva Foundation (FAP), producer of the BCG vaccine in Brazil. Both strains were cultured as surface pellicles for 2 weeks at 37°C in Sauton medium (18).

### Surface Fraction Preparation

After the removal of the culture filtrate, the surface associated material was obtained through vigorous manual shaking of the bacterial pellicle with 2 mm glass beads, essentially as described (19). The surface components were recovered in Milli-Q sterile water and centrifuged twice at 2,500 *g* for 10 min at 4°C. Aliquots of 1 mL were further centrifuged twice at 16,000 *g* in order to remove any remaining bacteria. The surface-associated protein fraction was obtained using an adaptation of the method described by Wessel and Flugge (20). Briefly, proteins were precipitated with 15% TCA/acetone, the resulting pellets were washed sequentially with 400 μL of 100% cold acetone, 200 μL of diethyl ether, and 200 μL of chloroform. The final pellets were resuspended in 100 μL Isoelectric focusing (IEF) buffer (8M ureum/2% CHAPS).

### Bi-Dimensional Electrophoresis

The immobilized pH gradient (IPG) strips and all 2DE reagents were purchased from Bio-Rad, (Hercules, CA, USA). For the first dimension, 500 μg of proteins were diluted to a final volume of 300 μL of rehydration solution (8M urea, 2% CHAPS), 4 mM Tributyl phosphine (TBP), 0.4% ampholytes pH 3-10, trace of bromophenol blue). The samples were applied to IPG strips (17 cm, pH interval of 4–7) by in-gel rehydration and incubated for 1 h at room temperature. All isoelectric focusing was performed on a Protean® IEF cell (Bio-Rad) with a temperature of 20°C and a maximum current of 50 μA/strip. Running conditions: active rehydration (50V) for 11 h; step 1- linear gradient from 1 to 250V over 20 min; step 2 - linear gradient from 250 to 10,000V over 2 h; step 3- constant 10,000V until complete 80,000 V/h. After isoelectric focusing, proteins were reduced in 130 mM dithiothreitol (DTT) and alkylated in 270 mM iodoacetamide, both in equilibration buffer (6M urea, 2% SDS, 375 mM Tris-HCl pH 8.8, 20% glycerol). Second dimension separation was done in 17 cm, 12% SDS-PAGE gels, 1.0 mm thick, using a vertical system (Bio-Rad) in standard Tris/glycine/SDS buffer at 40 mA/gel, 10°C, until the tracking dye left the gel. Proteins were visualized with Comassie Brilliant Blue (CBB) following procedures described elsewhere (21).

### Image Analysis

Gel images were documented using a GS-800^TM^ calibrated imaging densitometer (Bio-Rad) and images were analyzed using PDQuest^TM^ software (Bio-Rad). During the alignment of the images, to compensate for subtle differences in sample loading, gel staining, and destaining, the volume of each spot was normalized in relation to the total density of valid spots present in the gel image. Comparison of 2DE maps derived from three independent protein preparations, each one obtained from three independent BCG cultures, was performed. To determine experimental isoelectric point (p*I*) and molecular mass (*M*_r_) coordinates for each single spot, 2DE gels were calibrated using a select set of reliable identification landmarks distributed throughout the entire gel. The theoretical p*I* and *M*_r_ of proteins identified by mass spectrometry were obtained using the BCG Moreau RDJ genome reference sequence (9; accession number: AM412059.2).

### Protein Digestion, Peptide Extraction and MALDI-TOF/TOF Analysis

In-gel digestion of the 2DE SDS-PAGE separated proteins was carried out using the procedure according to Shevchenko (22). Briefly, protein spots were excised, and the gel pieces were washed three times with 50% (v/v) acetonitrile in 25 mM ammonium bicarbonate for 15 min each, dehydrated in acetonitrile, and dried in a vacuum centrifuge. Gel pieces were rehydrated in 15 μL of 50 mM ammonium bicarbonate containing 20 ng of sequencing grade modified trypsin (Promega). After 15 min, 20 μL of 50 mM ammonium bicarbonate was added to keep the gel pieces wet during tryptic digestion (37°C, 16 h). To extract peptides, 20 μL of 0.5% (v/v) trifluoroacetic acid (TFA) in 50% (v/v) acetonitrile were added and samples were sonicated for 30 min. The separated liquid was concentrated under vacuum to an approximate volume of 10 μL. The resulting peptides were extracted, partially dried, and salts were removed using ZipTipC18 columns (Millipore, Bedford, MA) following the manufacturer's instructions. The tryptic peptides were analyzed on a 4700-Proteomics Analyzer MALDI-TOF/TOF (Applied Biosystems, Foster City, CA). All mass spectra were acquired on positive ion reflector mode with 2,000 shots per spot and externally mass calibrated with a peptide mixture. The 10 most intense ion peaks from the peptide mass fingerprinting (or MS run) were further submitted to fragmentation using post source decay (PSD) mode with collision induced dissociation (CID) gas off and 1 keV collision energy.

### Data Analyses and Protein Identification

Following MS/MS acquisition, the processed data files (ppw files) from the MALDI-TOF/TOF were analyzed on a Mascot Server license v. 2.2 (23, 24). The mass spectra were searched against the *M. bovis* BCG str. Moreau protein database (9; accession number: AM412059.2). The parameters used for the search were as follows: peptide and fragment ions mass tolerance was set at 0.5 Da; maximum of one miss cleavage site by trypsin; carbamidomethylation of cysteine residues as fixed modification, whereas oxidation of methionine/tryptophan, acetylation of the N-terminal, pyroglutamic acid, pyroglutamine, and deamidation of asparagine/glutamine were considered as variable modifications. Positive protein hit identification was accepted with at least 1 matched unique peptide. False discovery rate was estimated at <1%. The peptide ion score was considered >15 with a significance threshold of *p* < 0.05, whereas the protein score was above 20. Finally, a good correlation between the experimental and theoretical molecular mass and p*I* was also considered for positive identifications. The mass spectrometry proteomics data have been deposited to the ProteomeXchange Consortium via the PRIDE partner repository (25) with the dataset identifier PXD006141.

### Statistical Analysis

Differences between spot intensity observed in 2DE gel images of *M. bovis* BCG strains Moreau and Pasteur were considered statistically significant when ^*^p < 0.05; ^**^p < 0.01; or ^***^p < 0.001, as determined by Graph Pad Prism 4.0 software (Graph- Pad Software Inc., San Diego, CA, USA). The unpaired Student‘s *t*-test was used to analyze the significant differences among both strains, using data obtained from at least 3 different sets of independent biological samples.

### Bioinformatic Data

Regions of difference RD2 and RD14 both present on BCG Moreau were evaluated in terms of protein identification and predicted function according to BCG Moreau genome while the region of difference RD16 absent from BCG Moreau was evaluated according to *M. tuberculosis* H37Rv reference genome (accession numbers: AM412059.2 and NC_000962, respectively). For this purpose, TubercuList (http://genolist.pasteur.fr/TubercuList/) was used as the reference site to find the information about predicted function and amino acid sequence from all the proteins in *M. tuberculosis* H37Rv encompassing RD2, RD14, and RD16 allowing the construction of Table 1.

**Table 1:** Proteins encoded by the genes localized in the regions of difference RD2, RD14, and RD16.

**Region of Difference**	**ORFs completely removed from the genome**	**Orthologs H37Rv/BCG Moreau**	**Protein identification**	**Predicted function**
RD2 (10,787 bp)	Rv1979c–Rv1987	Rv1979c/BCGM_1980c	Possible conserved permease	Unknown; Possibly involved in transport of amino acid across the membrane
		Rv1980c/mpb64/BCGM_1981c	Immunogenic protein mpt64 (antigen mpt64/mpb64)	Unknown
		Rv1981c/nrdF1/BCGM_1982c	Ribonucleoside-diphosphate reductase (beta chain)NRDF1 (Ribonucleotide reductase small subunit) (R2F protein)	Involved in the DNA replication pathway. Catalyzes the biosynthesis of deoxyribonucleotides from the corresponding ribonucleotides, precursors that are necessary for DNA synthesis (catalytic activity: 2-deoxyribonucleoside diphosphate + oxidized thioredoxin + H2O = ribonucleoside diphosphate + reduced thioredoxin)
		Rv1982c/BCGM_1983c	Conserved hypothetical protein	Unknown
		Rv1983/PE_PGRS35/BCGM_1984	PE-PGRS family protein	Unknown
		Rv1984c/cfp21/BCGM_1985c	Probable cutinase precursor cfp21	Hydrolyzes cutin
		Rv1985c/BCGM_1986c	Probable transcriptional regulatory protein (probably	Involved in transcriptional mechanism
			Lysr- family	
		Rv1986/BCGM_1987	Probable conserved integral membrane protein	Unknown; possibly involved in transport of lysine across the membrane
		Rv1987/BCGM_1988	Possible chitinase	Hydrolysis of chitin
RD14 (9,068 bp)	Rv1765A–Rv1772	Rv1765A/BCGM_1774c	Putative transposase (fragment)	Possibly required for the transposition of an insertion element
		Rv1766/BCGM_1775	Conserved hypothetical protein	Unknown
		Rv1767/BCGM_1776	Conserved hypothetical protein	Unknown
		Rv1768/PE_PGRS31/BCGM_1777	PE-PGRS family protein	Unknown
		Rv1769/BCGM_1778	Conserved hypothetical protein	Unknown
		Rv1770/BCGM_1779	Conserved hypothetical protein	Unknown
		Rv1771/BCGM_1780	Probable oxidoreductase	Unknown; Probably involved in cellular metabolism
		Rv1772/BCGM_1781	Hypothetical protein	Unknown
RD16 (7,608 bp)	Rv3401–Rv3404c	Rv3401/*Δ	Conserved hypothetical protein	Unknown; Probably enzyme involved in cellular metabolism
		Rv3402c/*Δ	Conserved hypothetical protein	Unknown; Thought to be involved in cell process
		Rv3403c/*Δ	Hypothetical protein	Unknown
		Rv3404c/*Δ	Conserved hypothetical protein	Unknown

*Δ *means deleted from the genome of BCG Moreau*.

Putative signal peptide for protein export were predicted using SignalP 4.1 (26–28), LipoP 1.0 (29, 30), TatP 1.0 (31, 32) and SecretomeP 2.0 (33, 34) in order to predict protein localization. Potential transmembrane domains were predicted with TMHMM 2.0 (35, 36). Beta-barrel membrane proteins structural subclass from integral membrane proteins were discriminated using a Hidden Markov Model method with PRED-TMBB (37–39).

The protein sequences described with moonlighting function were retrieved from the MoonProt (40, 41) or Multitasking Protein databases (42) as well as references on **Table 3** and Table S3. These sequences were used to construct the sequence database used in comparison to the sequences of the proteins identified in this work. The similarity search was performed using BLASTP (BLOSUM62 matrix), assuming the results with bitscore > 50 and *E*-value < e-10. Domain analyses were accomplished with proteins that obtained hit in the MoonProt and Multitasking databases using CDD (43, 44) and PFAM (45, 46).

## Results

### Proteins Encoded by the Genes From Regions of Difference RD2, RD14, and RD16

According to the literature, the regions of difference RD2 and RD14 are present in BCG Moreau, whereas RD16 is absent, when compared to BCG Pasteur (5). Table 1 lists the proteins encoded by these RDs. Our analysis on the surface associated proteome map of BCG Moreau (**Figure 2** and Table 2) detected Mpb64, encoded in RD2, and revealed the Rv3406 protein, whose regulation is affected by the truncation of gene *rv3405c*, due to deletion of RD16 (10).

**Table 2:** Differential surface-associated proteins between *M. bovis* BCG Moreau and *M. bovis* BCG Pasteur.

**Spot no**.	**Gene**	**Protein identification**	Fold difference M/P**	***p*-value**
32*	*BCGM_3440*	Alpha-ketoglutarate dependent sulfate ester dioxygenase	14.15	0.0017
33*			518.63	0.0021
39	*mpb70*	Secreted immunogenic protein Mpb70	4.10	0.1366
49*			23.74	<0.0001
50			1585.00	0.0558
51*			75.11	0.0346
52			0.38	0.2983
56*			8.99	0.0031
58			19.74	0.0709
43*	*mpb64*	Immunogenic protein Mpt64 (lost in BCG Pasteur due to RD2)	9.66	0.0022
44*			21.67	0.0002
119	*ahpC*	Alkyl hydroperoxide reductase C protein	1.63	0.0885
120			3.11	0.0524
121*			13.70	<0.0001
26*	*adoK*	Adenosine kinase	7.71	0.0217
14	*fadA3*	Probable beta-ketoacyl CoA thiolase	2.56	0.0538
24*			6.26	0.0104
145			1.09	0.8296
146*			2.47	0.0464
60*	*clpP2*	ATP-dependent Clp protease proteolytic subunit 2	5.57	0.0302
75			1.43	0.1921
42*	*BCGM_0830c*	Fatty acid binding protein-like protein (UPF0678)	5.14	0.0043
64	*echA3*	Probable enoyl-CoA hydratase (crotonase)	88.56	0.4390
118*			4.95	<0.0001
38*	*fixB*	Electron transfer flavoprotein (alpha-subunit)	4.12	0.0361
133			1.25	0.2959
134			1.23	0.5248
67*	*ppiA*	Iron regulated peptidyl-prolyl cis-trans isomerase A	3.81	0.0267
68			3.04	0.1288
69			1.37	0.5389
117			0.79	0.3860
9	*apa*	Alanine and proline rich secreted protein	0.90	0.7515
147*			2.83	0.0125
148*			3.40	0.0015
46*	*cfp17. garA*	Glycogen accumulation regulator GarA	1.76	0.0120
47			0.47	0.1294
48*			3.39	0.0160
149*	*TB39.8. fhaA*	FHA domain-containing protein	3.04	0.0311
150			2.42	0.1602
151			1.04	0.9438
152			1.29	0.4435
154*	*BCGM_1880c*	Probable reductase	2.44	0.0296
155			1.46	0.1402
124*	*prcB*	20S Proteasome (beta subunit)	1.93	0.0229
4*	*dnaK*	Chaperone protein DnaK (Hsp70)	1.62	0.0271
21*	*fadA*	Possible acyl-CoA thiolase	0.26	0.0025
107*	*esxJ*	ESAT-6 like protein EsxJ	0.51	0.0081
74	*TB18.6*	Conserved hypothetical protein (UPF0098)	0.50	0.2573
87*			0.56	0.0483
89			0.76	0.0707
5*	*groEL2*	Chaperonin 2, GroEL2 (65 kDa antigen; Hsp65)	0.68	0.0257
66*	*fba*	Fructose-biphosphate aldolase	0.69	0.0209
164			0.48	0.3384
122*	*gpm1*	Phosphoglycerate mutase 1	0.71	0.0310
72*	*ssb*	Single-strand binding protein	0.77	0.0467
73			0.41	0.0930

*Differences in the spot intensity of BCG Moreau and Pasteur 2DE were considered by performing unpaired statistical analysis*.

* *p < 0.05*.

** *Ratio of mean pixel intensity value for the specified protein spot in BCG Moreau (M) vs. Pasteur (P)*.

### Identification of *M. bovis* BCG Moreau Surface-Associated Proteins From 2DE Gels Using MALDI-TOF-TOF

**Figure 1:**
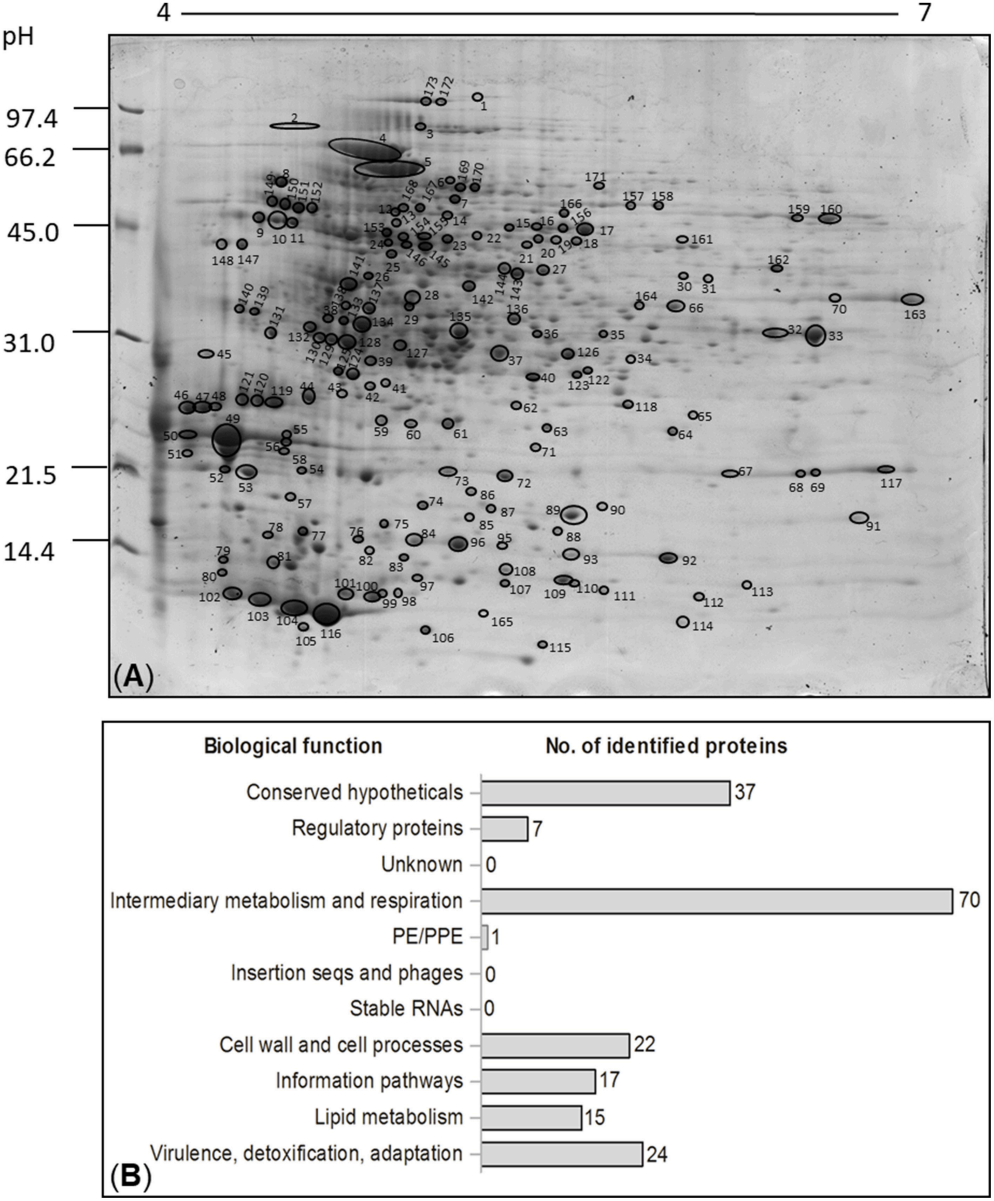
**(A)** Representative 2DE gel showing the proteomic profile of surface-associated proteins from the Brazilian vaccine strain, BCG Moreau. Molecular weight marker is indicated to the left in kDa. All identified proteins described in Table S1 are indicated and numbered. **(B)** Functional classification of all the identified proteins of the surface fraction from *M. bovis* BCG Moreau, cultured in Sauton medium. Categories of identified proteins grouped according to the biological function described on the left. The number of identified proteins is shown on the right. The classifications were generated using Tuberculist (http://genolist.pasteur.fr/TubercuList/).

The first goal of this study was to perform a proteomic analysis of surface-associated proteins of *M. bovis* BCG Moreau through 2DE gel electrophoresis and MALDI-TOF-TOF and compare it to *M. bovis* BCG Pasteur. The confirmation of the proteomic profile and the differences in protein expression between the two strains analyzed were done by comparing the surface proteomic maps in the pH 4–7 range through five biological replicates. Figure 1A shows a representative map of the surface-associated proteins of BCG Moreau. According to the statistical analysis and overlapping of the processed gel image (or virtual image), all replicates presented a reproducible profile related to the total number of spots as well as the localization (migration) and intensity (data not shown). A total of 173 protein spots are reported and they ranged in *Mr* between 19 and 97 kDa, mostly concentrated above the 31 kDa range. Analysis of the 2DE profiles showed that, in some cases, different spots were identified as representing the same peptide sequence (Table S1), possibly due to the occurrence of proteolysis, different protein isoforms, post-translational modifications (PTMs) or the formation of complexes or heterodimers between proteins. These events may cause differences between theoretical and experimental *Mr* and p*I*. The occurrence of different proteins identified in the same spot was also observed and can be explained by the limit of resolution in the 2DE technique (47). Among the 173 spots detected, 115 different proteins were identified by mass spectrometry (Table S1), which were classified according to the *M. bovis* BCG Moreau gene annotation and orthologs in *M. tuberculosis* H37Rv and *M. bovis* BCG Pasteur (accession numbers: AM412059.2, NC_000962 and AM408590.1, respectively). Functional classification of all identified proteins *per spot*, according to the TubercuList databank (http://genolist.pasteur.fr/TubercuList/) revealed that the major group composed by 70 proteins are related to intermediary metabolism/respiration whereas 37 proteins, the second major group, are conserved hypothetical proteins, without associated function (Figure 1B).

#### Bioinformatics Analyses of the Identified Surface-Associated Proteins

**Figure 2:**
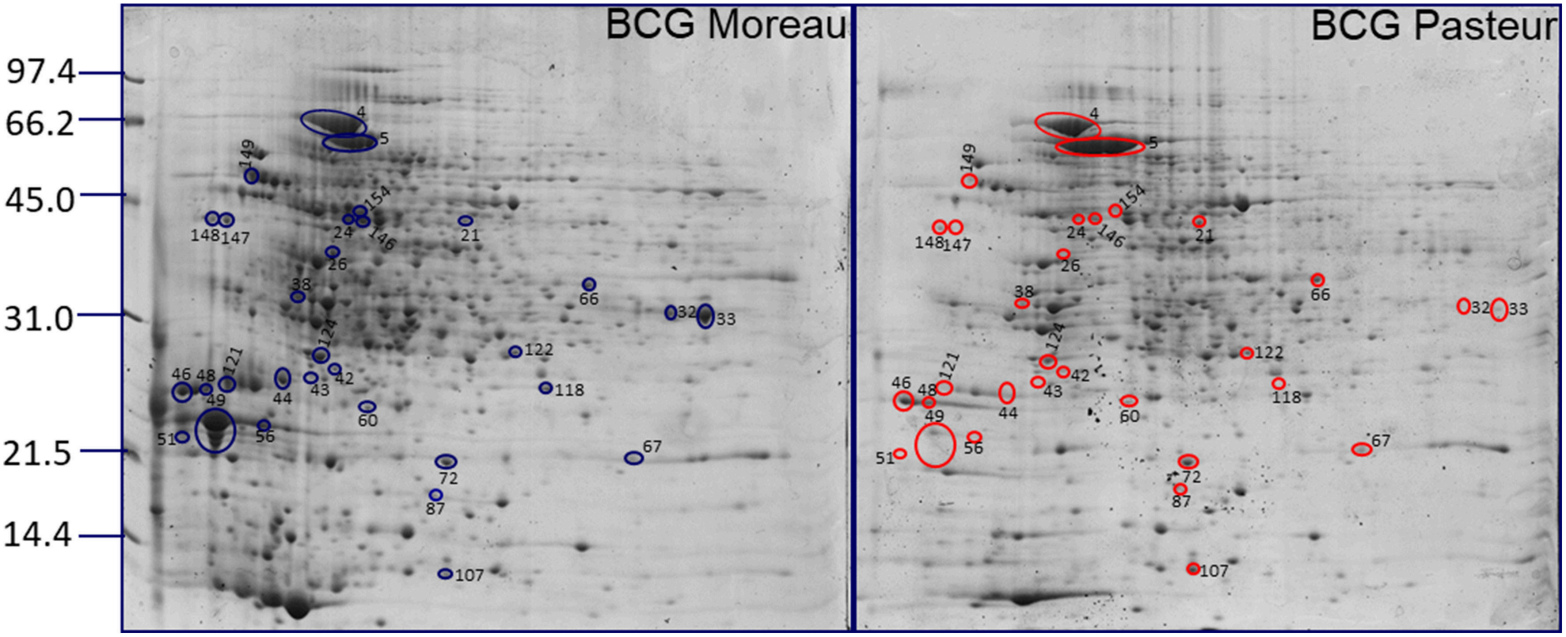
Differential surface-associated proteins between BCG Moreau and BCG Pasteur. Representative 2DE proteomic profiles between BCG Moreau **(left)** and BCG Pasteur **(right)** with differential spots (intensity) are marked and identified in blue (Moreau) and red (Pasteur). Molecular weight marker indicated to the left in kDa.

In order to predict the type of secretion process, transmembrane portion unit on the surface-associated proteins and their comparison with the membrane and secreted proteins already described in the literature, a combination of bioinformatic online programs and mycobacterium genome evaluation was used (Table S2). 2DE comparative quantification with non-paired *t*-test statistical analysis allowed the identification of 31 spots differentially expressed between BCG Moreau and Pasteur that represented 24 distinct proteins (Figure 2 and Table 2)−17 proteins were more abundant in BCG Moreau and 7 proteins in BCG Pasteur (Table 2). All 115 different proteins identified by mass spectrometry were searched against the Moonprot and Multitasking databases and we could predict moonlighting function for 27 of them (Table 3). Moreover, among the 27 moonlighting proteins, 5 of them were more abundant in BCG Moreau whereas 3 protein spots were up-regulated in BCG Pasteur (Table S3). Domain analysis on the moonlighting predicted proteins using CDD and pFam databases allowed the identification of two domain hits for fructose-biphosphate aldolase (Fba) and aldehyde dehydrogenase (AldC), thus, suggesting distinct functions (data not shown). However, the spot corresponding to the Fba protein was found 2-fold more expressed in BCG Pasteur than in BCG Moreau.

**Table 3:** *M. bovis* BCG Moreau surface-associated proteins with moonlighting predicted function.

**Gene**	**BCG Moreau**	**Predicted function**	**Moonlighting function**	**Organism/species**	**References**
*ppiA*	BCGM_0009	Probable iron-regulated peptidyl-prolyl cis-trans isomerase A	Proinflammatory cytokine-activate endothelial cells	*Homo sapiens*	(48)
			Induces apoptosis of gastric epithelial cells	*Helicobacter pylori*	(49)
			Ppiases might play a role in the intracellular survival by subverting the host cell defenses, such as oxidative stress as well as by immunomodulation	*Mycobacterium tuberculosis*	(50)
	BCGM_0129	Probable serine protease	Chaperone, aids in folding of proteins	*Escherichia coli*	(51)
				*Escherichia coli*	(52)
				*Escherichia coli*	(53)
*BCGM_0152*	BCGM_0152	Probable short-chain type dehydrogenase/reductase	Transcriptional repressor	*Homo sapiens*	(54)
*gabD1*	BCGM_0238c	Probable succinate-semialdehyde dehydrogenase [nadp+] dependant (ssdh)	Tau-crystallin/alpha-enolase	*Pseudemys scripta; Petromyzon marinus*	(55)
			Transcriptional repressor	*Escherichia coli; Salmonella typhimurium*	(56–59)
*fabG4*	BCGM_0247c	Probable 3-oxoacyl-[acyl-carrier protein] reductase	Transcriptional repressor	*Homo sapiens*	(54)
*dnaK*	BCGM_0357	Probable chaperone protein	Plasminogen binding	*Bifidobacterium*	(60)
				*Neisseria meningitides*	(61)
*fba §*	BCGM_0369c	Probable fructose-biphosphate aldolase	Plasminogen binding	*Candida albicans*	(62)
			V-ATPase assembly	*Saccharomyces cerevisiae*	(63–65)
			Adhesin	*Streptococcus pneumoniae; Neisseria meningitides*	(66, 67)
*groEL2*	BCGM_0447	60 kDa chaperonin 2, GroEL2	Toxin	*Enterobacter aerogenes*	(68)
			Invasion in a HeLa cell model	*Legionella pneumophila*	(69)
			Binds DNA	*Mycobacterium tuberculosis*	(70)
			Regulation of inflammation	*Mycobacterium leprae*	(71)
			Aggravation of atherosclerosis	*Chlamydia pneumoniae*	(72)
			Role in the growth of the bacteria	*Helicobacter pylori*	(73)
			Essential for biofilm formation	*Mycobacterium smegmatis*	(74)
			Stimulation of osteoclastogenesis	*Escherichia coli*	(75)
*Lpd*	BCGM_0470	Dihydrolipoamide dehydrogenase Lpd	Protease	*Homo sapiens*	(76)
*gpm1*	BCGM_0498	Probable phosphoglycerate mutase 1	Plasminogen binding protein	*Streptococcus sp. Oral*	(77)
*echA3*	BCGM_0644c	Probable enoyl-CoA hydratase	RNA and microtubule binding protein	Rice seed	(78)
*tuf*	BCGM_0699	Probable elongation factor TU tuf (EF-TU)	Attachment to human cells and mucins	*Lactobacillus johnsonii NCC533 (La1)*	(79)
			Fibronectin, plasminogen, and mucin binding	*Mycoplasma pneumoniae; Candida albicans; Streptococcus gordonii; Pseudomonas aeruginosa*	(62, 80–82)
			Cytoskeleton structure regulation	Cereal grains	(83)
*eno*	BCGM_1046	Probable enolase	Binds plasminogen, laminin, fibronectin, mucin, and actin	*Lactobacillus crispatus ST1 and Lactobacillusjohnsonii F133; Streptococcus gordonii; Lactobacillus plantarum; Streptococcus suis; Borrelia burgdorferi; Candida albicans; Staphylococcus aureus; Neisseria meningitidis; Aeromonas hydrophila; Echinococcus granulosus; Trichomonas vaginalis*	(61, 81, 84–92)
			It has neurotrophic and neuroprotective effects on rather a broad spectrum of neurons in the central nervous system (promotes cell survival)	*Rattus norvegicus*	(93)
			Activates homotypic vacuole fusion	*Saccharomyces cerevisiae*	(94)
			Depending on the localization may be involved in food vacuole formation or may play a role in transcription	*Plasmodium falciparum*	(95)
			Mitochondrial tRNA import	*Saccharomyces cerevisiae*	(96)
*glyA1*	BCGM_1119	Probable serine hydroxymethiltransferase 1	mRNA binds the 5' untranslated region (UTR) of its own mRNA	*Homo sapiens*	(97)
*fum*	BCGM_1124c	Probable fumarase fum(Fumarate hydratase)	Crystallins (alpha-, beta-gama-, delta-, epsilon- and tau- crystallins)	Vertebrate lenses	(98)
			Tumor suppressor	*Homo sapiens*	(99)
*gap*	BCGM_1465	Probable glyceraldehyde 3 phosphate dehydrogenase	Mucin, fibronectin, laminin, plasminogen, and fibrinogen binding protein	*Mycoplasma genitalium; Candida albicans; Candida albicans; Candida albicans; Streptococcus pneumoniae; enterohemorrhagic and enteropathogenic Escherichia coli*	(62, 100–103)
			Single-gene disorders	*Homo sapiens*	(104)
			Regulatory role in the bundling/unbundling of microtubules; involvements in the assembly of junctional triads from transverse tubules in skeletal muscle cells; protein kinase activity; single- stranded DNA-binding activity regulating transcription; human Nuclear uracil DNA glycocylase activity	Group A Streptococci	(105)
			Binds uPAR/CD87 receptor on human cells	*Streptococcus pyogenes*	(106)
			Gene-specific silencing of translation	Chordates	(107)
			Oral biofilm	*Streptococcus oralis*	(108)
			Transferrin receptor, NAD-ribosylating activity	Staphylococci	(109)
			Role in extracellular polysaccharide synthesis	*Xanthomonas campestris pv. Campestris*	(110)
*pgk*	BCGM_1466	Probable phosphoglycerate kinase	Interacts with plasminogen and its tissue activator	*Streptococcus pneumoniae*	(111)
			Acts in tumor angiogenesis as a disulphide reductase	*Homo sapiens*	(112)
*can*	BCGM_1505c	Probable aconitate hydratase Acn	Iron responsive element binding protein—iron uptake and use	mammalian tissues (beef liver)	(113)
			Iron-dependent RNA-binding activity—mRNA binding protein	*Homo sapiens; Mycobacterium tuberculosis*	(114, 115)
			Protection to mitochondrial DNA and regulation to mitochondrial DNA maintenance	*Saccharomyces cerevisiae*	(116, 117)
*BCGM_1880c*	BCGM_1880c	Probable reductase	Protease	Eukaryotes	(76)
*glnA1*	BCGM_2221	Glutamine synthetase	Control of gene expression	Bacteria	(118)
			Adhesion to host	*Lactobacillus crispatus ST1*	(119)
*ahpE*	BCGM_2239c	Peroxiredoxin AhpE	Plasminogen binding	*Candida albicans*	(62)
			Molecular chaperone function	Yeast	(120)
			Cell wall biogenesis	*Candida albicans*	(121)
*ahpC*	BCGM_2431	Alkyl hydroperoxide reductase c protein	Molecular chaperone function	Yeast	(120)
			Plasminogen binding	*Candida albicans*	(62)
			Cell wall biogenesis	*Candida albicans*	(121)
			Bifunctional enzyme with Glutathione peroxidase and phospholipase A2 activities	*Homo sapiens*	(122)
*ndkA*	BCGM_2449c	Probable nucleoside diphosphate kinase ndkA	Regulate cellular redox state and enhances multiple stress tolerance in transgenic plants	*Arabidopsis thaliana*	(123)
*aldC #*	BCGM_2863c	Probable aldehyde dehydrogenase	Eta-crystallin; Tau-crystallin/alpha-enolase	*Elephantulus edwardii; Anas platyrhynchos/Pseudemys scripta/Petromyzon marinus;*	(55, 124)
			Transcriptional regulator	*Salmonella typhimurium*	(59)
*adhC*	BCGM_3053	Probable NADP-dependent alcohol dehydrogenase	Zeta-crystallin	*Hyla japonica;* Guinea Pig	(125–128)
*groEL1*	BCGM_3451c	60 kDa chaperonin 1, GroEL1	Associated with nucleoids	*Mycobacterium tuberculosis*	(70)
			Toxin	*Enterobacter aerogenes*	(68)
			Invasion in a HeLa cell model	*Legionella pneumophila*	(69)
			Involved in mycolic acid biosynthesis during biofilm formation in mycobacteria	*Mycobacterium smegmatis*	(74)
			Regulation of inflammation	*Mycobacterium leprae*	(71)
			Regulates macrophage tumor necrosis factor-alpha and matrix metalloproteinase expression	*Chlamydia pneumoniae*	(72)
			Role in the growth of the bacteria	*Helicobacter pylori*	(73)
			Stimulator of osteoclast formation	*Escherichia coli*	(75)
*groES*	BCGM_3452c	10 kDa chaperonin	Activate human macrophages in synergy with LPS	*Francisella tularensis*	(129)

§ *Two domain hits: FTBP_aldolase_II (canonical hit) and ICL_KPHMT (moonlighting hit)*.

# *Two domain hits: ALDH_F1AB_F2_RALDH1 (canonical hit) and putA (moonlighting hit)*.

## Discussion

In this study, the surface-associated proteins from *M. bovis* BCG Moreau were investigated by 2DE combined with MALDI-TOF-TOF and bioinformatic analysis. Two-dimensional gel electrophoresis maps in the pH range of 4–7 led to the identification of 173 spots (Figure 1A) that could be assigned by mass spectrometry to a total of 115 different proteins (Table S1). This choice of pH range was based on previous analysis carried out in the broader pH range of 3–10, which showed that the majority of protein spots occurred in the pH range of 4–7 (data not shown). These results complement our previous report on the secretome of BCG strains Moreau and Pasteur (12) (Table 1).

The genome of BCG Moreau differs from BCG Pasteur, among others, by the presence of two regions (RD2 and RD14; 5). Several proteins encoded in these two regions have been described as potentially immunogenic (130–133), reinforcing the importance of performing studies to address the protein expression in these two strains. In this context, differentially expressed proteins present on the bacterial surface can partially account for some differences observed in individual response to vaccination. Among the proteins identified by mass spectrometry, the Mpb64 protein (encoded in RD2) was observed to be expressed in *M. bovis* BCG Moreau. The quantification performed by the PDQuest software indicated a very low expression of the Mpb64 protein in BCG Pasteur proteome map, probably due to the background observed in the area where the protein should be located (Figure 2, spots 43 and 44).

Mpb64 is an immunodominant antigen capable of inducing protective immunity by T cell response; however the role of this protein in the pathogenesis of tuberculosis is not known (134, 135). We found Mpb64 expression on the cell surface proteome of BCG Moreau (spots 43 and 44, Figure 1A) with the expected predicted signal peptide and transmembrane domains (Table S2), suggesting that vaccination with BCG Moreau could trigger a better cellular immune response when compared to BCG Pasteur. With respect to RD16, this characteristic deletion found in BCG Moreau (between genes *rv3400*, and *rv3405c)* leads to the truncation and functional loss of a TetR transcriptional regulator encoded by *rv3405c*, resulting in the constitutive expression of the adjacent *rv3406* gene (10); the resulting protein (Rv3406) was identified in the surface proteome of BCG Moreau, and is absent in Pasteur (Figure 2).

Considering the surface-associated proteomic profiles from *M. bovis* BCG Moreau and Pasteur, differences between the experimental and theoretical protein molecular mass and pI were observed for 49% of the identified spots, which may be due to post-translational modifications (PTMs), protein degradation, the presence of isoforms and conformers of the proteins (136, 137). For example, Apa (spots 9, 147, and 148) is already described as glycosylated on diverse threonine residues, which could result in the observation of multiple protein species by 2DE gel (138). In fact, the experimental molecular mass and pI of spots 9, 147, and 148 are in disagreement to the theoretical ones (Table S1). We observed that the ESAT-6 like protein EsxJ and glutamine synthetase (GS) found in spots 107 and 169/170, respectively, were identified as N-formylation-containing proteins (Table S1). Such PTM could contribute to electrophoretic mobility shift in 2DE gels, and therefore, would account for the differences between experimental and theoretical pI. It has been reported that N-formylated peptides may serve as good candidates for a universal vaccine against *M. tuberculosis* when administered in combination with drugs (139–141).

Overall, these PTMs could be important factors for eliciting the immune response. It has been reported that the glycosylated motif found in Apa protein was related to the high capacity of BCG to stimulate an immune response in BCG-immunized guinea pigs (142, 143). Our results indicate that the Apa protein shows higher expression on the cell surface of BCG Moreau compared to Pasteur (Figure 2 and Table 2). Other important proteins identified in the BCG Moreau surface-associated proteome were secreted antigens 85A and B (spots 37, 126, and 127 of Figure 1A). These proteins belong to the antigen Ag-85 complex and are found in association with the mycobacteria cell surface, constituting the major secreted proteins observed on mycobacteria culture filtrate (12, 144, 145). These proteins are strongly immunogenic and can trigger both humoral and cellular immune responses *in vivo* (144, 146–148). Ag85 proteins have mycolyltransferase activity, crucial to maintain the cell wall integrity of mycobacteria (149–151). In addition, they can bind to human fibronectin (152) and elastin (153) present in the extracellular matrix having, therefore, moonlighting functions. Recent studies demonstrated that Ag-85 complex was identified as a target for mannose-binding lectin and ficolins (154). These proteins have been extensively studied as potential candidates for new vaccines against tuberculosis (155, 156).

Protein fate in terms of cellular localization is an important aspect that might be explored on surface-associated proteins (Table S2). Non-covalently surface-associated proteins can be secreted through different systems such as via signal peptide (as predicted by Signal P and Lipo P software), non-classical secretory pathway like proteins without an N-terminal signal peptide (according to Secretome P analysis), and twin-arginine translocation pathway (as found by Tat prediction program) in which a twin-arginine consensus motif is located within the signal peptide itself (29, 31, 157–159). The non-classical secretory system was predicted for aconitase (spots 172 and 173) and glutamine synthetase (spots 169 and 170). In *M. tuberculosis*, aconitase is found in the cytosol, cell wall, and cell membrane fractions and it has also been described as a bifunctional protein acting as an enzyme in the presence of iron and RNA-binding in the absence of iron (115, 160). Glutamine synthetase (GS) presented moonlighting features of acyltransferase in *M. tuberculosis* (161). Previous studies have shown that this enzyme is secreted into the culture medium and plays a crucial role in pathogenicity as well as in bacterial growth (162, 163). In *Lactobacillus crispatus*, GS is a novel adhesive moonlighting enzyme that associates to the cell surface at an acidic pH (119). Further experiments must be carried out in order to show that aconitase, glutamine synthetase and other surface-associated proteins (Table 3) have moonlighting behavior also in *M. bovis* BCG Moreau.

Bioinformatic tools have been used to predict protein moonlighting function by primary sequence analysis and hence domain investigations of predicted moonlighting proteins can be pursued to propose novel functions that corroborate with *in vitro* and *in vivo* studies (40, 42, 164, 165). Nevertheless, most moonlighting proteins described to date have been identified by chance (166). In general, highly conserved proteins, often metabolic proteins/enzymes (167, 168) or molecular chaperones (169), receptors (170), ribosomal proteins, and transmembrane channels (171), were shown to be moonlighting proteins (172). These findings suggest that the presence of intracellular proteins at “unexpected” locations is not always due to experimental artifacts such as cellular lysis. The methodology used here for obtaining the fraction of surface-associated proteins is well characterized (19), strengthening the fact that the intracellular proteins identified in this study may be performing moonlighting functions. In addition, the appearance of a new function for the same protein can be considered a great advantage for the microorganism since it optimizes the functional repertoire encoded by a compact genome. In this context, bioinformatic investigation using the Moonprot/Multitask programs led to the prediction of moonlighting functions for some of the identified proteins, already described for other organisms/species (Table 3). Besides, Conserved Domains Database (CDD) and pFam were also used to infer the number of domains these proteins could present (data not shown). Most of the identified proteins grouped in the “intermediary metabolism” functional category (Figure 1B), raising the question of why proteins normally encountered intracellularly had been found surface-associated to the mycomembrane. This could be explained by different functions these proteins may have depending on localization. Finally, other features such as the presence of distinct protein surfaces or domains influencing the oligomeric state of the protein, concentration of cellular ligands, substrates and cofactors must also be considered to presume moonlighting function for these kind of proteins (16, 173–175).

Interestingly, we found that fructose-biphosphate aldolase (Fba) and the aldehyde dehydrogenase (AldC) contained two domains hit: one representative of a canonical function in mycobacteria and another representing a probable moonlighting function already described in other organisms (Table 3). In mycobacteria, the canonical domain hit for fructose-biphosphate aldolase is FTBP_aldolase_II representative of an enzyme that controls the condensation of dihydroxyacetone phosphate with glyceraldehyde-3-phosphate to yield fructose-1,6-bisphosphate (176). Nevertheless, the moonlighting domain hit is ICL_KPHMT that represents an enzyme superfamily that catalyzes the formation and cleavage of either P-C (proline-cysteine) or C-C (cysteine-cysteine) bonds (data not shown). In other organisms, as described in Table 3, the moonlighting function of fructose- biphosphate aldolase is related to protein binding and cell adherence. (177) confirmed experimentally by *in vitro* assays that Fba from *M. tuberculosis* binds to human plasminogen. This generates the proteolytic enzyme plasmin leading to the breakdown of extracellular matrix and basal membrane proteins, contributing to tissue injury in tuberculosis. More recently, de la Paz Santangelo et al. (178) reported that Fba of *M. tuberculosis* binds to human plasminogen in a dose dependent manner and is important for *M. tuberculosis* growth. According to our results Fba is less expressed on the cell surface of BCG Moreau than in BCG Pasteur (Table S3). On the other hand, the canonical domain hit for aldehyde dehydrogenase is ALDH_F1AB_F2_RALDH1 that corresponds to NAD+-dependent retinal dehydrogenase 1 also known as aldehyde dehydrogenase family 1 member A1 (ALDH1A1) in humans. It is a cytosolic enzyme that catalyzes the oxidation of retinaldehyde to retinoic acid (RA). RA is the active metabolite of vitamin A and it is required for spermatogenesis and many other biological processes (179). The moonlighting domain hit is PutA, a trifunctional protein in bacteria: transcriptional regulator, proline dehydrogenase and pyrroline-5- carboxylate dehydrogenase (data not shown). Christgen et al. (180) discovered a membrane binding region on the PutA domain from *Escherichia coli* AldC that explains the PutA functional switch from self-regulating transcriptional repressor to membrane binding domain. Our results indicate that AldC is a surface-associated protein from *M. bovis* BCG Moreau (spot 171 of Figure 1A) not found in the culture filtrate of BCG Moreau (Table S2). Fba and AldC may play a role in the immunopathology of tuberculosis, but this still needs further investigation.

Altogether, the differences in abundance of surface-associated proteins identified between BCG strains Moreau and Pasteur could have an impact on vaccine efficacy. The finding that some of these proteins have moonlighting functions opens new possibilities for investigating the role of extracellular proteins on the bacterial-host interface.

## Author Contributions

TP, MB-P, and LM-L conceived, designed, performed experiments and analyzed results. AG performed bioinformatic analysis. MW and PC performed statistical analysis of results. DK organized, analyzed, processed, and deposited the mass spectrometry data on PRIDE repository. WD conceived and designed experiments. TP, DK, PC, MB-P, and LM-L wrote the paper.

### Conflict of Interest Statement

The authors declare that the research was conducted in the absence of any commercial or financial relationships that could be construed as a potential conflict of interest.

## Abbreviations

TB: Tuberculosis
WHO: World Health Organization
BCG: Bacillus Calmette-Guerin
SNPs: single nucleotide polymorphisms
RD: Region of Difference
2DE gel: Two-dimensional gel electrophoresis
PTMs: Post-translational modifications
FH: Fumarate hydratase
TCA: trichloroacetic acid
GS: glutamine synthetase
TDM: trehalose dimycolate
CDD: Conserved Domains Database
Fba: fructose-biphosphate aldolase
AldC: aldehyde dehydrogenase.
