# Supplementary material for: Exploring the Potential Role of Moonlighting Function of the Surface-Associated Proteins From Mycobacterium bovis BCG Moreau and Pasteur by Comparative Proteomic
Source: Front Immunol. 2019 Apr 26;10:716. doi: 10.3389/fimmu.2019.00716 (PMC6497762; doi:10.3389/fimmu.2019.00716)
Supplement: Supplementary file 1 [file Table_1.DOCX]

***Supplementary material***

**Exploring the potential role of moonlighting function of the surface-associated proteins from *Mycobacterium bovis* BCG Moreau and Pasteur by comparative proteomic**

## Talita Duarte Pagani^1Ɨ*^, Ana Carolina R. Guimarães^1^, Mariana C. Waghabi^1^, Paloma Rezende Corrêa^1^, Dário Eluan Kalume^2,3^, Marcia Berrêdo-Pinho^4^, Wim Maurits Degrave^1^ and Leila Mendonça-Lima^1*^

^1^Laboratório de Genômica Funcional e Bioinformática, Instituto Oswaldo Cruz, Fundação Oswaldo Cruz, Rio de Janeiro, RJ, Brazil

^2^Laboratório Interdisciplinar de Pesquisas Médicas (LIPMED), Instituto Oswaldo Cruz—IOC, Fundação Oswaldo Cruz—FIOCRUZ, Rio de Janeiro,Brazil and

^3^Unidade de Espectrometria de Massas e Proteômica (UEMP), Instituto de Bioquímica Médica Leopoldo de Meis, Universidade Federal do Rio de Janeiro - UFRJ, Rio de Janeiro, Brazil

^4^Laboratório de Microbiologia Celular, Instituto Oswaldo Cruz, Fundação Oswaldo Cruz, Rio de Janeiro, RJ, Brazil

^Ɨ^ Present address: Laboratório de Genômica Estrutural, Instituto de Biofísica Carlos Chagas Filho, Universidade Federal do Rio de Janeiro, Rio de Janeiro, RJ, Brazil

* Co-corresponding authors

Correspondence:Dr Talita Duarte Pagani ([tdpagani@yahoo.com); Dr](mailto:tdpagani@yahoo.com);%20Dr) Leila Mendonça-Lima ( lmlima@ioc.fiocruz.br)

**Table S1. Identified surface-associated proteins from *M. bovis* BCG Moreau by MS/MS.** The protein classification was also made based in H37Rv and BCG Pasteur orthologs. Functional Classification (FC) are reported for the proteins with numbers given by Tuberculist (TubercuList, 2004) .

Table S1.

| **Spot no.** | **gene** | **BCG Moreau** | **Orthologs H37Rv / Pasteur** | **Protein Identification** | **FC** | **Protein score** | **Peptide Sequence** | **Delta^&^ (Da)** | **Ion Score** | **Cov (%)** | **Theor *M*_r_  (kDa)** | **Theor pI** | **Exp *M*_r_ (kDa)** | **Exp  pI** |
| --- | --- | --- | --- | --- | --- | --- | --- | --- | --- | --- | --- | --- | --- | --- |
| 1 | *metE* | BCGM_1160c | Rv1133c | Probable 5-methyltetrahydropteroyl triglutamate- | 7 | 142 | ADQAEYLR | 0.0020 | 33 | 11 | 81.5 | 5.13 | 113.8 | 5.3 |
|  |  |  | BCG1194c | homocysteine methyltransferase |  |  | ATEGYWAGR | 0.0117 | 46 |  |  |  |  |  |
|  |  |  |  |  |  |  | AVDGAGAPIER | 0.0039 | 29 |  |  |  |  |  |
|  |  |  |  |  |  |  | RADQAEYLR | 0.0043 | 14 |  |  |  |  |  |
|  |  |  |  |  |  |  | AGEIDEAEYVR | 0.0083 | 40 |  |  |  |  |  |
|  |  |  |  |  |  |  | QPFTATITGSPR | 0.0089 | 50 |  |  |  |  |  |
|  |  |  |  |  |  |  | LGLDVLVHGEPER | 0.0141 | 32 |  |  |  |  |  |
|  |  |  |  |  |  |  | LHLPPLPTTTIGSYPQTSAIR | 0.0350 | 46 |  |  |  |  |  |
| 2 | *metE* | BCGM_1160c | Rv1133c | Probable 5-methyltetrahydropteroyl triglutamate- | 7 | 71 | AGEIDEAEYVR | 0.0693 | 51 | 4 | 81.5 | 5.13 | 78.5 | 4.6 |
|  |  |  | BCG1194c | homocysteine methyltransferase |  |  | ^c^QPFTATITGSPR | 0.0635 | 21 |  |  |  |  |  |
|  |  |  |  |  |  |  | QPFTATITGSPR | 0.0574 | 29 |  |  |  |  |  |
|  |  |  |  |  |  |  | DGHDAVADEIASSR | 0.0732 | 25 |  |  |  |  |  |
| 3 | *dlaT* | BCGM_2215 | Rv2215 | Dihydrolipoamide acyltransferase, E2 component of | 7 | 73 | APPAPAPALAHLR | 0.0494 | 10 | 4 | 57.0 | 4.90 | 86.7 | 5.1 |
|  |  |  | BCG2231 | pyruvate dehydrogenase |  |  | IHPNINASYNEDTK | 0.0554 | 73 |  |  |  |  |  |
| 4 | *dnaK* | BCGM_0357 | Rv0350 | Probable chaperone protein | 0 | 112 | DAGQIAGLNVLR | -0.0398 | 12 | 10 | 66.8 | 4.85 | 66.1 | 5.0 |
|  |  |  | BCG0389 |  |  |  | NQAETLVYQTEK | -0.0513 | 36 |  |  |  |  |  |
|  |  |  |  |  |  |  | ATSGDNHLGGDDWDQR | -0.0623 | 32 |  |  |  |  |  |
|  |  |  |  |  |  |  | SETFTTADDNQPSVQIQVYQGER | -0.1147 | 86 |  |  |  |  |  |
| 5 | *groEL2* | BCGM_0447 | Rv0440 | 60 kDa chaperonin 2, GroEL2 | 0 | 103 | TIAYDEEAR | -0.0228 | 19 | 9 | 56.7 | 4.85 | 59.7 | 5.1 |
|  |  |  | BCG0479 |  |  |  | EIELEDPYEK | -0.0308 | 17 |  |  |  |  |  |
|  |  |  |  |  |  |  | ^c^QEIENSDSDYDR | -0.0332 | 13 |  |  |  |  |  |
|  |  |  |  |  |  |  | DETTIVEGAGDTDAIAGR | -0.0515 | 105 |  |  |  |  |  |
| 6 | *groEL1* | BCGM_3451c | Rv3417c | 60 kDa chaperonin 1, GroEL1 | 0 | 94 | LIEYDETAR | -0,0002 | 25 | 10 | 55.8 | 4.98 | 56.0 | 5.3 |
|  |  |  | BCG3487c |  |  |  | EVGLEVLGSAR | -0.0171 | 12 |  |  |  |  |  |
|  |  |  |  |  |  |  | AFGGPTVTNDGVTVAR | -0.0077 | 54 |  |  |  |  |  |
|  |  |  |  |  |  |  | AAVEEGIVPGGGASLIHQAR | -0.0102 | 59 |  |  |  |  |  |
| 7 | *gabD1* | BCGM_0238c | Rv0234c | Probable succinate-semialdehyde dehydrogenase | 7 | 189 | TFTAATDDEVDAAIAR | 0.0278 | 71 | 17 | 54.3 | 5.36 | 52.5 | 5.3 |
|  |  |  | BCG0271c | [nadp+] dependent (ssdh) |  |  | FIVHADIYDDFVDK | 0.0151 | 57 |  |  |  |  |  |
|  |  |  |  |  |  |  | HASNVPQC^d^ALYLADVIAR | 0.0504 | 75 |  |  |  |  |  |
|  |  |  |  |  |  |  | DMALYTEEVFGPVASVFR | 0.0539 | 19 |  |  |  |  |  |
|  |  |  |  |  |  |  | AANIDEAVEIANATTFGLGSNAWTR | 0.0764 | 54 |  |  |  |  |  |
| 8 | *tig* | BCGM_2466c | Rv2462c | Probable trigger factor (tf) protein | 3 | 592 | NQLPTMFADVR | 0.0203 | 23 | 28 | 50.6 | 4.43 | 54.1 | 4.5 |
|  |  |  | BCG2482c |  |  |  | FNELLVEQGSSR | 0.0296 | 104 |  |  |  |  |  |
|  |  |  |  |  |  |  | LIAGLDDAVVGLSADESR | 0.0389 | 114 |  |  |  |  |  |
|  |  |  |  |  |  |  | LAAGEHAGQEAQVTVTVR | 0.0378 | 90 |  |  |  |  |  |
|  |  |  |  |  |  |  | INVEVPFAELEPDFQR | 0.0469 | 102 |  |  |  |  |  |
|  |  |  |  |  |  |  | ^a^QYGIEPQ^c^QLFGYLQER | 0.0591 | 44 |  |  |  |  |  |
|  |  |  |  |  |  |  | ^a^QYGIEPQQLFGYLQER | 0.0521 | 82 |  |  |  |  |  |
|  |  |  |  |  |  |  | EYGQDLQFTAEVDIRPK | 0.0426 | 106 |  |  |  |  |  |
|  |  |  |  |  |  |  | ELPEPDDEFAQLASEFDSIDELR | 0.0749 | 111 |  |  |  |  |  |
| 9 | *apa* | BCGM_1871 | Rv1860 | Alanine and proline rich secreted protein | 3 | 170 | LGSDMGEFYMPYPGTR | 0.0762 | 49 | 27 | 32.7 | 4.93 | 45.5 | 4.4 |
|  |  |  | BCG1896 |  |  |  | TTGDPPFPGQPPPVANDTR | 0.0647 | 59 |  |  |  |  |  |
|  |  |  |  |  |  |  | INQETVSLDAN^a^GVSGSASYYEVK | 0.0869 | 77 |  |  |  |  |  |
|  |  |  |  |  |  |  | FSDPSKPN^a^GQIWTGVIGSPAANAPDAGPPQR | 0.1348 | 49 |  |  |  |  |  |
|  | *eno* | BCGM_1046 | Rv1023 | Probable enolase | 7 | 48 | AAADSAELPLFR | 0.0402 | 34 | 6 | 44.8 | 4.47 | 45.5 | 4.4 |
|  |  |  | BCG1080 |  |  |  | VQIVGDDIFVTNPER | 0.0554 | 37 |  |  |  |  |  |
|  |  |  |  |  |  |  |  |  |  |  |  |  |  |  |

Table S1 *(continued)*

| **Spot no.** | **gene** | **BCG Moreau** | **Orthologs H37Rv / Pasteur** | **Protein Identification** | **FC** | **Protein score** | **Peptide Sequence** | **Delta^&^ (Da)** | **Ion Score** | **Cov (%)** | **Theor *M*_r_  (kDa)** | **Theor pI** | **Exp *M*_r_ (kDa)** | **Exp  pI** |
| --- | --- | --- | --- | --- | --- | --- | --- | --- | --- | --- | --- | --- | --- | --- |
| 10 | *eno* | BCGM_1046 | Rv1023 | Probable enolase | 7 | 159 | PIIEQVGAR | -0.0147 | 19 | 22 | 44.8 | 4.47 | 45.0 | 4.5 |
|  |  |  | BCG1080 |  |  |  | YAGDLAFPR | -0,0175 | 50 |  |  |  |  |  |
|  |  |  |  |  |  |  | WGAEVYHALK | -0.0238 | 24 |  |  |  |  |  |
|  |  |  |  |  |  |  | AAADSAELPLFR | -0.0189 | 41 |  |  |  |  |  |
|  |  |  |  |  |  |  | VQIVGDDIFVTNPER | -0.0262 | 70 |  |  |  |  |  |
|  |  |  |  |  |  |  | AAVPSGASTGEHEAVELR | -0.0257 | 20 |  |  |  |  |  |
|  |  |  |  |  |  |  | AVQAVLDEIGPAVIGLNADDQR | -0.0282 | 60 |  |  |  |  |  |
| 11 | *eno* | BCGM_1046 | Rv1023 | Probable enolase | 7 | 538 | PIIEQVGAR | 0.0269 | 58 | 38 | 44.8 | 4.47 | 44.7 | 4.5 |
|  |  |  | BCG1080 |  |  |  | YAGDLAFPR | 0.0258 | 65 |  |  |  |  |  |
|  |  |  |  |  |  |  | WGAEVYHALK | 0.0024 | 61 |  |  |  |  |  |
|  |  |  |  |  |  |  | AAADSAELPLFR | 0.0289 | 67 |  |  |  |  |  |
|  |  |  |  |  |  |  | VQIVGDDIFVTNPER | 0.0383 | 119 |  |  |  |  |  |
|  |  |  |  |  |  |  | AAVPSGASTGEHEAVELR | 0.0468 | 58 |  |  |  |  |  |
|  |  |  |  |  |  |  | GNPTVEVEVALIDGTFAR | 0.0480 | 83 |  |  |  |  |  |
|  |  |  |  |  |  |  | AVQAVLDEIGPAVIGLNADDQR | 0.0704 | 77 |  |  |  |  |  |
|  |  |  |  |  |  |  | VNQIGTLTETLDAVTLAHHGGYR | 0.0753 | 65 |  |  |  |  |  |
|  |  |  |  |  |  |  | EGLSTGLGDEGGFAPDVAGTTAALDLISR | 0.0957 | 88 |  |  |  |  |  |
| 12 | *manA* | BCGM_3268c | Rv3255c | Probable mannose-6-phosphate isomerase | 7 | 27 | HVDVPELLR | -0.0273 | 27 | 5 | 43.3 | 4.98 | 48.5 | 5.0 |
|  |  |  | BCG3284c |  |  |  | GTAAWVAADDGPIR | -0.0395 | 16 |  |  |  |  |  |
| 13 | *atpD* | BCGM_1337 | Rv1310 | Probable ATP synthase beta chain atpD | 7 | 25 | VVDLLTPYVR | -0.0325 | 17 | 4 | 53.1 | 4.86 | 46.3 | 5.0 |
|  |  |  | BCG1370 |  |  |  | VTGPVVDVEFPR | -0.0447 | 25 |  |  |  |  |  |
| 14 | *fadA3* | BCGM_1098c | Rv1074c | Probable beta-ketoacyl CoA thiolase | 1 | 259 | NPLFDGAQER | 0.0116 | 66 | 17 | 42.6 | 4.92 | 48.0 | 5.3 |
|  |  |  | BCG1132c |  |  |  | AGEGDAFISAGVETVSR | 0.0183 | 97 |  |  |  |  |  |
|  |  |  |  |  |  |  | VVAVALGYDFLPGTTVNR | 0.0159 | 51 |  |  |  |  |  |
|  |  |  |  |  |  |  | EITPVTLPDGTTVSTDDGPRPGTTYEK | 0.0353 | 110 |  |  |  |  |  |
|  | *ino1* | BCGM_0047c | Rv0046c | Myo-inositol-1-phosphate synthase | 7 | 62 | SPPEQLPDDIAR | 0.0191 | 62 | 3 | 39.9 | 4.96 | 48.0 | 5.3 |
|  |  |  | BCG0077c |  |  |  |  |  |  |  |  |  |  |  |
| 15 | *tuf* | BCGM_0699 | Rv0685 | Probable elongation factor TU tuf (EF-TU) | 2 | 32 | AFDQIDNAPEER | 0.0911 | 32 | 7 | 43.6 | 5.28 | 44.3 | 5.5 |
|  |  |  | BCG0734 |  |  |  | ELLAAQEFDEDAPVVR | 0.1196 | 16 |  |  |  |  |  |
|  | *glpX* | BCGM_1125c | Rv1099c | Fructose-1,6-bisphosphatase class 2 | 10 | 31 | HAQLIHDVR | 0.0682 | 31 | 2 | 34.5 | 5.06 | 44.3 | 5.5 |
|  |  |  | BCG1150c |  |  |  |  |  |  |  |  |  |  |  |
| 16 | *tuf* | BCGM_0699 | Rv0685 | Probable elongation factor TU tuf (EF-TU) | 2 | 27 | AFDQIDNAPEER | 0.0093 | 20 | 7 | 43.6 | 5.28 | 45.1 | 5.5 |
|  |  |  | BCG0734 |  |  |  | ELLAAQEFDEDAPVVR | 0.0136 | 26 |  |  |  |  |  |
| 17 | *tuf* | BCGM_0699 | Rv0685 | Probable elongation factor TU tuf (EF-TU) | 2 | 275 | AFDQIDNAPEER | 0.0171 | 69 | 17 | 43.6 | 5.28 | 44.0 | 5.7 |
|  |  |  | BCG0734 |  |  |  | LLDQGQAGDNVGLLLR | 0.0138 | 122 |  |  |  |  |  |
|  |  |  |  |  |  |  | ELLAAQEFDEDAPVVR | 0.0120 | 93 |  |  |  |  |  |
|  |  |  |  |  |  |  | GQVVTKPGTTTPHTEFEGQVYILSK | 0.0086 | 56 |  |  |  |  |  |
| 18 | *kasB* | BCGM_2247 | Rv2246 | 3-oxoacyl-[acyl-carrier protein] synthase 2 | 1 | 59 | IEAVPIAGFAQMR | 0.0452 | 15 | 10 | 46.4 | 5.29 | 43.3 | 5.6 |
|  |  |  | BCG2263 |  |  |  | AINNALGGNRPAVYAPK | 0.0561 | 37 |  |  |  |  |  |
|  |  |  |  |  |  |  | TLDDPFVEEFDLPVR | 0.0560 | 42 |  |  |  |  |  |
| 19 | *argG* | BCGM_1664 | Rv1658 | Probable Argininosuccinate synthase | 7 | 43 | ALDC^d^GAVEAVVVDAR | 0.0433 | 43 | 10 | 43.6 | 5.26 | 43.4 | 5.6 |
|  |  |  | BCG1697 |  |  |  | SAESLYDFNLATYDEGDSFDQSAAR | 0.0569 | 18 |  |  |  |  |  |
| 20 | *fadE25* | BCGM_3287c | Rv3274c | Probable acyl-CoA dehydrogenase | 1 | 69 | ITQIYEGTNQIQR | 0.0498 | 34 | 6 | 41.7 | 5.21 | 43.4 | 5.5 |
|  |  |  | BCG3303c |  |  |  | GSPTTELYFENC^d^R | 0.0504 | 55 |  |  |  |  |  |
| 21 | *fadA* | BCGM_0876 | Rv0859 | Possible acyl-CoA thiolase | 1 | 69 | EDVDAYALR | 0.0473 | 19 | 7 | 42.3 | 5.20 | 41.6 | 5.5 |
|  |  |  | BCG0911 |  |  |  | AAEAWSGGYFAK | 0.0512 | 46 |  |  |  |  |  |
|  |  |  |  |  |  |  | SEEAFIYEAIR | 0.0569 | 46 |  |  |  |  |  |

Table S1 *(continued)*

| **Spot no.** | **gene** | **BCG Moreau** | **Orthologs H37Rv / Pasteur** | **Protein Identification** | **FC** | **Protein score** | **Peptide Sequence** | **Delta^&^ (Da)** | **Ion Score** | **Cov (%)** | **Theor *M*_r_  (kDa)** | **Theor pI** | **Exp *M*_r_ (kDa)** | **Exp  pI** |
| --- | --- | --- | --- | --- | --- | --- | --- | --- | --- | --- | --- | --- | --- | --- |
| 22 | *tuf* | BCGM_0699 | Rv0685 | Probable elongation factor TU tuf (EF-TU) | 2 | 180 | AFDQIDNAPEER | 0.0483 | 71 | 11 | 43.6 | 5.28 | 43.5 | 5.4 |
|  |  |  | BCG0734 |  |  |  | LLDQGQAGDNVGLLLR | 0.0410 | 80 |  |  |  |  |  |
|  |  |  |  |  |  |  | ELLAAQEFDEDAPVVR | 0.0476 | 76 |  |  |  |  |  |
| 23 | *icl* | BCGM_0475 | Rv0467 | Isocitrate lyase | 7 | 38 | VLIPTQQHIR | 0.0289 | 18 | 5 | 47.0 | 5.03 | 43.2 | 5.3 |
|  |  |  | BCG0507 |  |  |  | SAEQIQQEWDTNPR | 0.0313 | 38 |  |  |  |  |  |
| 24 | *fadA3* | BCGM_1098c | Rv1074c | Probable beta-ketoacyl CoA thiolase | 1 | 65 | VVAVALGYDFLPGTTVNR | -0.2575 | 65 | 4 | 42.6 | 4.92 | 42.6 | 5.0 |
|  |  |  | BCG1132c |  |  |  |  |  |  |  |  |  |  |  |
| 25 | *pgk* | BCGM_1466 | Rv1437 | Probable phosphoglycerate kinase | 7 | 40 | GLLETYHDVLR | -0.0139 | 10 | 10 | 42.5 | 4.83 | 41.4 | 5.0 |
|  |  |  | BCG1498 |  |  |  | AEGLTGGDILLLENIR | -0.0172 | 18 |  |  |  |  |  |
|  |  |  |  |  |  |  | SDLNVPLDEDGTITDAGR | -0.0203 | 40 |  |  |  |  |  |
| 26 | *adoK* | BCGM_2202c | Rv2202c | Adenosine kinase | 7 | 49 | FSEQLLPEHLHK | -0.0143 | 37 | 8 | 34.4 | 4.75 | 38.7 | 4.9 |
|  |  |  | BCG2218c |  |  |  | LGLAFAADPSQQLAR | -0.0097 | 32 |  |  |  |  |  |
| 27 | *gap* | BCGM_1465 | Rv1436 | Probable glyceraldehyde 3-phosphate dehydrogenase | 7 | 447 | GKLDGYALR | 0.0406 | 19 | 27 | 35.9 | 5.19 | 40.0 | 5.6 |
|  |  |  | BCG1497 |  |  |  | VLDDEFGIVK | 0.0161 | 52 |  |  |  |  |  |
|  |  |  |  |  |  |  | VVSWYDNEWGYSNR | 0.0818 | 80 |  |  |  |  |  |
|  |  |  |  |  |  |  | VVSW^e^YDNEWGYSNR | 0.0832 | 62 |  |  |  |  |  |
|  |  |  |  |  |  |  | LPC^d^DVGLEGDDTIVVGR | 0.0715 | 175 |  |  |  |  |  |
|  |  |  |  |  |  |  | VPIPTGSVTDLTVDLSTR | 0.0679 | 133 |  |  |  |  |  |
|  |  |  |  |  |  |  | YYDAPIVSSDIVTDPHSSIFDSGLTK | 0.0946 | 53 |  |  |  |  |  |
|  | *adhC* | BCGM_3053 | Rv3045 | NADP-dependent alcohol dehydrogenase | 7 | 65 | DGQPTQGGYSEAIVVDENYVLR | 0.0923 | 65 | 6 | 37.0 | 5.11 | 40.0 | 5.6 |
|  |  |  | BCG3069 |  |  |  |  |  |  |  |  |  |  |  |
| 28 | *BCGM_0289* | BCGM_0289 | Rv0281 | Putative S-adenosyl-L-methionine-dependent | 10 | 70 | YFDEYFR | 0.0418 | 27 | 12 | 33.0 | 4.88 | 37.0 | 5.1 |
|  |  |  | BCG0321 | methyltransferase |  |  | STDFGEHFVNFQGAR | 0.0541 | 66 |  |  |  |  |  |
|  |  |  |  |  |  |  | SDPLVVDPYAEAF^d^CR | 0.0569 | 22 |  |  |  |  |  |
| 29 | *BCGM_1802* | BCGM_1802 | Rv1794 | Conserved hypothetical protein | 10 | 53 | VLAAPDLEVVALLSR | 0.0500 | 22 | 10 | 32.4 | 4.86 | 35.4 | 5.1 |
|  |  |  | BCG1826 |  |  |  | LLYGVIDDENQPPGSR | 0.0569 | 53 |  |  |  |  |  |
| 30 | *thrC* | BCGM_1322 | Rv1295 | Probable threonine synthase | 7 | 38 | AIDDGWVAR | 0.0278 | 38 | 6 | 37.3 | 5.75 | 39.3 | 6.0 |
|  |  |  | BCG1355 |  |  |  | GYTEYHQLGLIDK | 0.0371 | 22 |  |  |  |  |  |
| 31 | *pafB* | BCGM_2100c | Rv2096c | Proteasome accessory factor B PafB | 10 | 29 | VTEVPTGEQATVWVAEGR | 0.0369 | 16 | 20 | 35.3 | 5.68 | 39.4 | 6.1 |
|  |  |  | BCG2116c |  |  |  | IGAQVTPIGPAGATTVPAGVDLR | 0.0436 | 29 |  |  |  |  |  |
|  |  |  |  |  |  |  | AAGVDVDPLDTGAPVAIASAAAVSGLR | 0.0610 | 14 |  |  |  |  |  |
| 32 | *BCGM_3440* | BCGM_3440 | Rv3406 | Alpha-ketoglutarate-dependent sulfate ester dioxygenase | 7 | 71 | VLFEVLQR | 0.0190 | 11 | 18 | 32.5 | 5.99 | 33.2 | 6.5 |
|  |  |  | BCG3476 |  |  |  | ^c^QVFEKPDFR | 0.0226 | 20 |  |  |  |  |  |
|  |  |  |  |  |  |  | QVFEKPDFR | 0.0256 | 21 |  |  |  |  |  |
|  |  |  |  |  |  |  | SFVGLDSHESR | 0.0260 | 24 |  |  |  |  |  |
|  |  |  |  |  |  |  | LGGDLDPAAVNEIR | 0.0248 | 37 |  |  |  |  |  |
|  |  |  |  |  |  |  | YDYVTTKPLTAAQR | 0.0266 | 51 |  |  |  |  |  |
| 33 | *BCGM_3440* | BCG_M3440 | Rv3406 | Alpha-ketoglutarate-dependent sulfate ester dioxygenase | 7 | 91 | TLLAGDFVR | 0.0037 | 26 | 20 | 32.5 | 5.99 | 32.5 | 6.7 |
|  |  |  | BCG3476 |  |  |  | VLFEVLQR | -0.0030 | 33 |  |  |  |  |  |
|  |  |  |  |  |  |  | QVFEKPDFR | 0.0024 | 28 |  |  |  |  |  |
|  |  |  |  |  |  |  | SFVGLDSHESR | 0.0053 | 24 |  |  |  |  |  |
|  |  |  |  |  |  |  | AIDDYDDQHR | -0.0044 | 24 |  |  |  |  |  |
|  |  |  |  |  |  |  | LGGDLDPAAVNEIR | 0.0006 | 69 |  |  |  |  |  |
|  |  |  |  |  |  |  |  |  |  |  |  |  |  |  |
|  |  |  |  |  |  |  |  |  |  |  |  |  |  |  |
|  |  |  |  |  |  |  |  |  |  |  |  |  |  |  |
|  |  |  |  |  |  |  |  |  |  |  |  |  |  |  |

Table S1 *(continued)*

| **Spot no.** | **gene** | **BCG Moreau** | **Orthologs H37Rv / Pasteur** | **Protein Identification** | **FC** | **Protein score** | **Peptide Sequence** | **Delta^&^ (Da)** | **Ion Score** | **Cov (%)** | **Theor *M*_r_  (kDa)** | **Theor pI** | **Exp *M*_r_ (kDa)** | **Exp  pI** |
| --- | --- | --- | --- | --- | --- | --- | --- | --- | --- | --- | --- | --- | --- | --- |
| 34 | *deoD_1* | BCGM_3320 | Rv3307 | Probable purine nucleoside phosphorylase DeoD | 7 | 251 | IHAYEGHDLR | -0.0000 | 61 | 32 | 27.5 | 5.51 | 30.2 | 5.8 |
|  |  |  | BCG3372 | (inosine phosphorylase) |  |  | ADPRPDPDELAR | -0.0041 | 15 |  |  |  |  |  |
|  |  |  |  |  |  |  | SPLVGGEFVDLTDAYSPR | 0.0064 | 106 |  |  |  |  |  |
|  |  |  |  |  |  |  | ADLQVGQPVLISDHLNLTAR | 0.0033 | 69 |  |  |  |  |  |
|  |  |  |  |  |  |  | ^c^QSDPQLAEGVYAGLPGPHYETPAEIR | 0.0178 | 47 |  |  |  |  |  |
|  |  |  |  |  |  |  | QSDPQLAEGVYAGLPGPHYETPAEIR | 0.0117 | 56 |  |  |  |  |  |
| 35 | *BCGM_1368* | BCGM_1368 | Rv1339 | Conserved hypothetical protein | 10 | 45 | LVAHPTESFGLR | 0.0398 | 27 | 20 | 29.1 | 5.80 | 33.3 | 5.7 |
|  |  |  | BCG1401 |  |  |  | ALLYGPSDTWSR | 0.0398 | 40 |  |  |  |  |  |
|  |  |  |  |  |  |  | ELLLTHIPPWTSR | 0.0437 | 15 |  |  |  |  |  |
|  |  |  |  |  |  |  | AEFDGPVHAVVC^d^DETFEVR | 0.0723 | 14 |  |  |  |  |  |
| 36 | *tsf* | BCGM_2893c | Rv2889c | Probable elongation factor TSF | 2 | 88 | DDVPEDIVASER | 0.0347 | 20 | 16 | 28.8 | 5.26 | 32.9 | 5.5 |
|  |  |  | BCG2910c |  |  |  | SADLPPAVGVLVEYR | 0.0447 | 40 |  |  |  |  |  |
|  |  |  |  |  |  |  | GDDAAAAHAVALQIAALR | 0.0548 | 71 |  |  |  |  |  |
| 37 | *fbpA* | BCGM_3830c | Rv3804c | Secreted antigen 85-A | 1 | 27 | FLEGFVR | -0.0190 | 18 | 8 | 35.7 | 6.08 | 31.2 | 5.3 |
|  |  |  | BCG3866c |  |  |  | VWVYC^d^GN^a^GKPSDLGGN^a^NLPAK | -0.0412 | 28 |  |  |  |  |  |
| 38 | *fixB* | BCGM_3035c | Rv3028c | Probable electron transfer flavoprotein (alpha-subunit) | 7 | 95 | AAVDSGYYPGQFQVGQTGK | -0.0390 | 50 | 15 | 31.7 | 4.71 | 33.2 | 4.7 |
|  |  |  | BCG3051c |  |  |  | AGAVEAEPAAGAGEQVSVEVPAAAENAAR | -0.0843 | 68 |  |  |  |  |  |
| 39 | *mpb70* | BCGM_2880 | Rv2875 | Secreted immunogenic protein mpb70 | 3 | 154 | ^c^QTLQGASVTVTGQGNSLK | -0.0821 | 94 | 9 | 19.1 | 4.75 | 30.2 | 5.0 |
|  |  |  | BCG2897 |  |  |  | QTLQGASVTVTGQGNSLK | -0.0804 | 83 |  |  |  |  |  |
| 40 | *BCGM_0152* | BCGM_0152 | Rv0148 | Probable short-chain type dehydrogenase/reductase | 7 | 188 | VHLYGGYHVLR | 0.0046 | 87 | 39 | 29.8 | 5.26 | 28.8 | 5.6 |
|  |  |  | BCG0184 |  |  |  | YNIHANALAPIAATR | 0.0052 | 51 |  |  |  |  |  |
|  |  |  |  |  |  |  | TALDEFGAVHGVVSNAGILR | -0.0006 | 76 |  |  |  |  |  |
|  |  |  |  |  |  |  | EYALTLAGEGASVVVNDLGGAR | -0.0016 | 23 |  |  |  |  |  |
|  |  |  |  |  |  |  | VVVATSTSGLFGNFGQTNYGAAK | 0.0023 | 29 |  |  |  |  |  |
|  |  |  |  |  |  |  | VALFGNDGANFDKPPSVQDVAAR | 0.0096 | 28 |  |  |  |  |  |
| 41 | *BCGM_1885* | BCGM_1885 | Rv1874 | Hypothetical protein | 10 | 24 | AFFGAADDNDLR | 0.0396 | 15 | 14 | 24.9 | 4.78 | 27.7 | 5.0 |
|  |  |  | BCG1910 |  |  |  | MVASTSAFGANQNIDTVPTSR | 0.0568 | 24 |  |  |  |  |  |
| 42 | *BCGM_0830c* | BCGM_0830c | Rv0813c | UPF0678 fatty acid-binding protein-like protein | 10 | 36 | NIPAFDDLPVPADTANLR | -0.0851 | 36 | 7 | 23.9 | 4.86 | 27.8 | 4.9 |
|  |  |  | BCG0865c |  |  |  |  |  |  |  |  |  |  |  |
| 43 | *mpb64* | BCGM_1981c | Rv1980c |  | 3 | 54 | AFDWDQAYR | -0.0602 | 36 | 17 | 24.8 | 4.84 | 27.0 | 4.7 |
|  |  |  |  |  |  |  | SLENYIAQTR | -0.0660 | 11 |  |  |  |  |  |
|  |  |  |  |  |  |  | EAPYELNITSATYQSAIPPR | -0.1186 | 37 |  |  |  |  |  |
| 44 | *mpb64* | BCGM_1981c | Rv1980c | Immunogenic protein Mpt64 | 3 | 215 | FLSAATSSTPR | 0.0107 | 15 | 29 | 24.8 | 4.84 | 26.7 | 4.6 |
|  |  |  |  |  |  |  | AFDWDQAYR | 0.0166 | 56 |  |  |  |  |  |
|  |  |  |  |  |  |  | SLENYIAQTR | 0.0157 | 37 |  |  |  |  |  |
|  |  |  |  |  |  |  | DKFLSAATSSTPR | 0.0193 | 44 |  |  |  |  |  |
|  |  |  |  |  |  |  | VYQNAGGTHPTTTYK | 0.0230 | 78 |  |  |  |  |  |
|  |  |  |  |  |  |  | EAPYELNITSATYQSAIPPR | 0.0342 | 84 |  |  |  |  |  |
| 45 | *argJ* | BCGM_1659 | Rv1653 | Probable glutamate n-acetyltransferase | 7 | 99 | VTVTVTGAATEDDALVAAR | -0.1435 | 46 | 8 | 41.1 | 4.70 | 29.6 | 4.1 |
|  |  |  | BCG1692 |  |  |  | TTDLSHAYVEENSAYSS | -0.1367 | 75 |  |  |  |  |  |
| 46 | *cfp17* | BCGM_1838 | Rv1827 | Conserved hypothetical protein | 10 | 386 | LVFLTGPK | 0.0309 | 39 | 45 | 17.2 | 4.29 | 25.6 | 4.1 |
|  |  |  | BCG1862 |  |  |  | FLLDQAITSAGR | 0.0419 | 94 |  |  |  |  |  |
|  |  |  |  |  |  |  | HPDSDIFLDDVTVSR | 0.0445 | 131 |  |  |  |  |  |
|  |  |  |  |  |  |  | EPVDSAVLANGDEVQIGK | 0.0420 | 72 |  |  |  |  |  |
|  |  |  |  |  |  |  | LENNEFNVVDVGSLN^a^GTYVNR | 0.0499 | 142 |  |  |  |  |  |
|  |  |  |  |  |  |  |  |  |  |  |  |  |  |  |
|  |  |  |  |  |  |  |  |  |  |  |  |  |  |  |

Table S1 *(continued)*

| **Spot no.** | **gene** | **BCG Moreau** | **Orthologs H37Rv / Pasteur** | **Protein Identification** | **FC** | **Protein score** | **Peptide Sequence** | **Delta^&^ (Da)** | **Ion Score** | **Cov (%)** | **Theor *M*_r_  (kDa)** | **Theor pI** | **Exp *M*_r_ (kDa)** | **Exp  pI** |
| --- | --- | --- | --- | --- | --- | --- | --- | --- | --- | --- | --- | --- | --- | --- |
| 47 | *cfp17* | BCGM_1838 | Rv1827 | Conserved hypothetical protein | 10 | 165 | LVFLTGPK | 0.0349 | 24 | 42 | 17.2 | 4.29 | 25.5 | 4.1 |
|  |  |  | BCG1862 |  |  |  | FLLDQAITSAGR | 0.0524 | 47 |  |  |  |  |  |
|  |  |  |  |  |  |  | HPDSDIFLDDVTVSR | 0.0658 | 91 |  |  |  |  |  |
|  |  |  |  |  |  |  | DQTSDEVTVETTSVFR | 0.0741 | 20 |  |  |  |  |  |
|  |  |  |  |  |  |  | EPVDSAVLAN^a^GDEVQIGK | 0.0731 | 73 |  |  |  |  |  |
|  | *mpb70* | BCGM_2880 | Rv2875 | Secreted immunogenic protein mpb70 | 3 | 26 | ^c^QTLQGASVTVTGQGNSLK | 0.0634 | 26 | 9 | 19.1 | 4.75 | 25.5 | 4.1 |
|  |  |  | BCG2897 |  |  |  |  |  |  |  |  |  |  |  |
| 48 | *cfp17* | BCGM_1838 | Rv1827 | Conserved hypothetical protein | 10 | 155 | LVFLTGPK | -0.0808 | 30 | 34 | 17.2 | 4.29 | 25.5 | 4.2 |
|  |  |  | BCG1862 |  |  |  | FLLDQAITSAGR | -0.1081 | 90 |  |  |  |  |  |
|  |  |  |  |  |  |  | HPDSDIFLDDVTVSR | -0.1456 | 65 |  |  |  |  |  |
|  |  |  |  |  |  |  | LENNEFNVVDVGSLN^a^GTYVNR | -0.1738 | 34 |  |  |  |  |  |
| 49 | *mpb70* | BCGM_2880 | Rv2875 | Secreted immunogenic protein mpb70 | 3 | 304 | LPASTIDELK | -0.0531 | 56 | 14 | 19.1 | 4.75 | 23.1 | 4.2 |
|  |  |  | BCG2897 |  |  |  | ^c^QTLQGASVTVTGQGNSLK | -0.0892 | 157 |  |  |  |  |  |
|  |  |  |  |  |  |  | QTLQGASVTVTGQGNSLK | -0.0954 | 137 |  |  |  |  |  |
| 50 | *mpb70* | BCGM_2880 | Rv2875 | Secreted immunogenic protein mpb70 | 3 | 69 | ^ac^QTLQGASVTVTGQGNSLK | 0.0317 | 44 | 9 | 19.1 | 4.75 | 24.0 | 4.0 |
|  |  |  | BCG2897 |  |  |  | QTLQGASVTVTGQGNSLK | 0.0111 | 48 |  |  |  |  |  |
| 51 | *mpb70* | BCGM_2880 | Rv2875, BCG2897 | Secreted immunogenic protein mpb70 | 3 | 118 | ^c^QTLQGASVTVTGQGNSLK | -0.0446 | 77 | 9 | 19.1 | 4.75 | 22.9 | 4.0 |
|  |  |  | BCG2897 |  |  |  | QTLQGASVTVTGQGNSLK | -0.0460 | 63 |  |  |  |  |  |
| 52 | *mpb70* | BCGM_2880 | Rv2875 | Secreted immunogenic protein mpb70 | 3 | 136 | ^c^QTLQGASVTVTGQGNSLK | 0.0411 | 83 | 23 | 19.1 | 4.75 | 21.4 | 4.2 |
|  |  |  | BCG2897 |  |  |  | QTLQGASVTVTGQGNSLK | 0.0508 | 74 |  |  |  |  |  |
|  |  |  |  |  |  |  | TNSSLLTSILTYHVVAGQTSPANVVGTR | 0.0886 | 21 |  |  |  |  |  |
|  | *tpx* | BCGM_1945 | Rv1932 | Probable thiol peroxidase | 0 | 51 | SVLLNIFPSVDTPV^d^CATSVR | 0.0614 | 51 | 12 | 16.9 | 4.36 | 21.4 | 4.2 |
|  |  |  | BCG1971 |  |  |  |  |  |  |  |  |  |  |  |
| 53 | *tpx* | BCGM_1945 | Rv1932 | Probable thiol peroxidase | 0 | 44 | DLPFAQK | -0.0038 | 44 | 4 | 16.9 | 4.36 | 21.0 | 4.3 |
|  |  |  | BCG1971 |  |  |  |  |  |  |  |  |  |  |  |
| 54 | *hadA* | BCGM_0649 | Rv0635 | (3R)-hydroxyacyl-ACP dehydratase subunit HadA | 10 | 93 | FEKPIVAGDK | -0.0319 | 14 | 18 | 17.5 | 4.51 | 21.4 | 4.6 |
|  |  |  | BCG0684 |  |  |  | YPDHYEVER | -0.0239 | 60 |  |  |  |  |  |
|  |  |  |  |  |  |  | LYC^d^DVYVDSVR | -0.0313 | 57 |  |  |  |  |  |
| 55 | *mpb70* | BCGM_2880 | Rv2875 | Secreted immunogenic protein mpb70 | 3 | 43 | ^c^QTLQGASVTVTGQGNSLK | -0.0292 | 28 | 9 | 19.1 | 4.75 | 24.2 | 4.5 |
|  |  |  | BCG2897 |  |  |  | QTLQGASVTVTGQGNSLK | -0.0176 | 34 |  |  |  |  |  |
| 56 | *mpb70* | BCGM_2880 | Rv2875 | Secreted immunogenic protein mpb70 | 3 | 195 | LPASTIDELK | -0.0088 | 48 | 14 | 19.1 | 4.75 | 23.8 | 4.5 |
|  |  |  | BCG2897 |  |  |  | ^c^QTLQGASVTVTGQGNSLK | -0.0072 | 95 |  |  |  |  |  |
|  |  |  |  |  |  |  | QTLQGASVTVTGQGNSLK | -0.0091 | 95 |  |  |  |  |  |
| 57 | *mpb83* | BCGM_2878 | Rv2873 | Cell surface lipoprotein Mpb83 | 3 | 74 | IDGTHQTLQGADLTVIGAR | 0.0329 | 74 | 8 | 22.0 | 4.86 | 19.1 | 4.5 |
|  |  |  | BCG2895 |  |  |  |  |  |  |  |  |  |  |  |
| 58 | *mpb70* | BCGM_2880 | Rv2875 | Secreted immunogenic protein mpb70 | 3 | 52 | ^a^QTL^c^QGASVTVTGQGNSLK | -0.0375 | 52 | 9 | 19.1 | 4.75 | 23.3 | 4.5 |
|  |  |  | BCG2897 |  |  |  |  |  |  |  |  |  |  |  |
| 59 | *BCGM_1494* | BCGM_1494 | Rv1465 | Possible nitrogen fixation related protein | 7 | 53 | DALAQASEAFEEVTDER | -0.0461 | 25 | 41 | 17.7 | 4.91 | 25.9 | 5.0 |
|  |  |  | BCG1526 |  |  |  | GTVPGDEDVLGDGVAFAGVAK | -0.0491 | 40 |  |  |  |  |  |
|  |  |  |  |  |  |  | VTDVSYDGQG^d^CSISQAATSVLTEQVIGQR | -0.1151 | 31 |  |  |  |  |  |
| 60 | *clpP2* | BCGM_2464c | Rv2460c | Probable ATP-dependent clp protease proteolytic subunit 2 | 7 | 70 | DYGIIDTVLEYR | -0.0378 | 52 | 17 | 23.5 | 4.99 | 25.6 | 5.1 |
|  |  |  | BCG2480c |  |  |  | ADIQTVC^d^LGQAASAAAVLLAAGTPGK | -0.0863 | 41 |  |  |  |  |  |
| 61 | *BCGM_1738c* | BCGM_1738c | Rv1732c | Conserved hypothetical protein | 10 | 142 | AFSAAC^d^TPDTFVFDGQR | -0.0395 | 127 | 20 | 19.4 | 5.02 | 25.5 | 5.2 |
|  |  |  | BCG1771c |  |  |  | GQLDDSRPGN^a^GRPVTAADVR | -0.0557 | 36 |  |  |  |  |  |
|  |  |  |  |  |  |  |  |  |  |  |  |  |  |  |
|  |  |  |  |  |  |  |  |  |  |  |  |  |  |  |

Table S1 *(continued)*

| **Spot no.** | **gene** | **BCG Moreau** | **Orthologs H37Rv / Pasteur** | **Protein Identification** | **FC** | **Protein score** | **Peptide Sequence** | **Delta^&^ (Da)** | **Ion Score** | **Cov (%)** | **Theor *M*_r_  (kDa)** | **Theor pI** | **Exp *M*_r_ (kDa)** | **Exp  pI** |
| --- | --- | --- | --- | --- | --- | --- | --- | --- | --- | --- | --- | --- | --- | --- |
| 62 | *BCGM_3019* | BCGM_3019 | Rv3013 | Conserved hypothetical protein | 10 | 56 | VSW^d^CTVLR | 0.0101 | 10 | 28 | 23.0 | 5.11 | 26.4 | 5.5 |
|  |  |  | BCG3035 |  |  |  | LQVLVNEAPR | 0.0150 | 38 |  |  |  |  |  |
|  |  |  |  |  |  |  | ANSAPWLPIER | 0.0181 | 31 |  |  |  |  |  |
|  |  |  |  |  |  |  | AAALDGGADWVPQAWR | 0.0250 | 28 |  |  |  |  |  |
|  |  |  |  |  |  |  | ELELLDHVAAAEGATAR | 0.0195 | 14 |  |  |  |  |  |
| 63 | *BCGM_3778c* | BCGM_3778c | Rv3755c | Conserved hypothetical protein | 10 | 218 | FGLTVTLAER | -0.0265 | 59 | 38 | 22.0 | 5.43 | 24.7 | 5.6 |
|  |  |  | BCG3814c |  |  |  | DEENM^e^WLVTDHQGER | -0.0491 | 12 |  |  |  |  |  |
|  |  |  |  |  |  |  | SPVADTTVTVDSDGFIVDYPGLAER | -0.0942 | 156 |  |  |  |  |  |
|  |  |  |  |  |  |  | IVAAATANNPAFGAHYDLQTDETGATK | -0.0803 | 48 |  |  |  |  |  |
| 64 | *echA3* | BCGM_0644c | Rv0632c | Probable enoyl-CoA hydratase | 1 | 50 | GGFELAYR | 0.0131 | 36 | 17 | 24.3 | 5.52 | 26.0 | 5.9 |
|  |  |  | BCG0679c |  |  |  | VFSGGFDLK | -0.0176 | 36 |  |  |  |  |  |
|  |  |  |  |  |  |  | SRADALTAIR | -0.0302 | 14 |  |  |  |  |  |
|  |  |  |  |  |  |  | EFAGLNQHAHAATK | -0.0284 | 15 |  |  |  |  |  |
| 65 | *frr* | BCGM_2886c | Rv2882c | Ribosome recycling factor | 2 | 331 | HKEGELLEV | -0.0112 | 41 | 33 | 20.8 | 5.71 | 26.6 | 6.1 |
|  |  |  | BCG2903c |  |  |  | VAVPQLTEER | 0.0020 | 65 |  |  |  |  |  |
|  |  |  |  |  |  |  | LVVIKPYEANQLR | 0.0029 | 85 |  |  |  |  |  |
|  |  |  |  |  |  |  | NSDLGVNPTNDGALIR | -0.0027 | 148 |  |  |  |  |  |
|  |  |  |  |  |  |  | TTHQYVTQIDELVK | -0.0401 | 84 |  |  |  |  |  |
| 66 | *fba* | BCGM_0369c | Rv0363c | Probable fructose-biphosphate aldolase | 7 | 215 | SEIEEALR | 0.0376 | 45 | 28 | 36.5 | 5.49 | 35.7 | 6.0 |
|  |  |  | BCG0401c |  |  |  | YPVNVALHTDHC^d^PK | 0.0360 | 59 |  |  |  |  |  |
|  |  |  |  |  |  |  | LRPDILAQ^a^GQQVAAAK | 0.0653 | 42 |  |  |  |  |  |
|  |  |  |  |  |  |  | DKLDSYVRPLLAISAQR | 0.0505 | 13 |  |  |  |  |  |
|  |  |  |  |  |  |  | YLLAATFGNVHGVYKPGNVK | 0.0583 | 60 |  |  |  |  |  |
|  |  |  |  |  |  |  | LGLPADAKPFDFVFHGGSGSLK | 0.0641 | 95 |  |  |  |  |  |
| 67 | *ppiA* | BCGM_0009 | Rv0009 | Probable iron-regulated peptidyl-prolyl cis-trans isomerase A | 2 | 80 | IALFGNHAPK | -0.0021 | 22 | 53 | 19.2 | 5.80 | 22.5 | 6.2 |
|  |  |  | BCG0009 |  |  |  | TVANFVGLAQGTK | -0.0092 | 57 |  |  |  |  |  |
|  |  |  |  |  |  |  | HTIFGEVIDAESQR | -0.0140 | 24 |  |  |  |  |  |
|  |  |  |  |  |  |  | VIQGFM^b^IQGGDPTGTGR | -0.0248 | 31 |  |  |  |  |  |
|  |  |  |  |  |  |  | TATDGNDRPTDPVVIESITIS | -0.0515 | 26 |  |  |  |  |  |
|  |  |  |  |  |  |  | DYSTQNASGGPSGPFYDGAVFHR | -0.0575 | 23 |  |  |  |  |  |
| 68 | *ppiA* | BCGM_0009 | Rv0009 | Probable iron-regulated peptidyl-prolyl cis-trans isomerase A | 2 | 129 | IALFGNHAPK | -0.0023 | 19 | 41 | 19.2 | 5.80 | 22.8 | 6.6 |
|  |  |  | BCG0009 |  |  |  | TVANFVGLAQGTK | -0.0091 | 82 |  |  |  |  |  |
|  |  |  |  |  |  |  | HTIFGEVIDAESQRHTIFGEVIDAESQR | -0.0248 | 12 |  |  |  |  |  |
|  |  |  |  |  |  |  | VIQGFM^b^IQGGDPTGTGR | -0.0217 | 69 |  |  |  |  |  |
|  |  |  |  |  |  |  | TATDGNDRPTDPVVIESITIS | -0.0422 | 17 |  |  |  |  |  |
| 69 | *ppiA* | BCGM_0009 | Rv0009 | Probable iron-regulated peptidyl-prolyl cis-trans isomerase A | 2 | 101 | IALFGNHAPK | -0.0080 | 16 | 33 | 19.2 | 5.80 | 22.8 | 6.7 |
|  |  |  | BCG0009 |  |  |  | TVANFVGLAQGTK | -0.0193 | 52 |  |  |  |  |  |
|  |  |  |  |  |  |  | VIQGFM^b^IQGGDPTGTGR | -0.0302 | 69 |  |  |  |  |  |
|  |  |  |  |  |  |  | TATDGNDRPTDPVVIESITIS | -0.0473 | 20 |  |  |  |  |  |
| 70 | *BCGM_0101* | BCGM_0101 | Rv0097 | Possible oxidoreductase | 7 | 71 | VWQ^a^SLPAAK | 0.0082 | 14 | 17 | 32.6 | 6.10 | 36.2 | 6.7 |
|  |  |  | BCG0130 |  |  |  | GTYFIDLAR | 0.0264 | 55 |  |  |  |  |  |
|  |  |  |  |  |  |  | TTPPIKWPTVIR | 0.0280 | 10 |  |  |  |  |  |
|  |  |  |  |  |  |  | TGQEILYIC^d^ATGTTK | 0.0107 | 34 |  |  |  |  |  |
|  |  |  |  |  |  |  | NLDDITTDEIRDIVYTNK | 0.0550 | 26 |  |  |  |  |  |
|  | *BCGM_0220* | BCGM_0220 | Rv0216 | Double hotdog hydratase | 7 | 50 | LVLDFYR | 0.0295 | 42 | 13 | 35.8 | 6.49 | 36.2 | 6.7 |
|  |  |  |  |  |  |  | FPAVGDTLYTR | 0.0309 | 30 |  |  |  |  |  |
|  |  |  |  |  |  |  | LTLNIAATHHDWR | 0.0520 | 17 |  |  |  |  |  |
|  |  |  |  |  |  |  | SLVYAVSDSASEPDR | 0.0447 | 27 |  |  |  |  |  |

Table S1 *(continued)*

| **Spot no.** | **gene** | **BCG Moreau** | **Orthologs H37Rv / Pasteur** | **Protein Identification** | **FC** | **Protein score** | **Peptide Sequence** | **Delta^&^ (Da)** | **Ion Score** | **Cov (%)** | **Theor *M*_r_  (kDa)** | **Theor pI** | **Exp *M*_r_ (kDa)** | **Exp  pI** |
| --- | --- | --- | --- | --- | --- | --- | --- | --- | --- | --- | --- | --- | --- | --- |
| 71 | *BCGM_0871* | BCGM_0871 | Rv0854 | Conserved hypothetical protein | 10 | 102 | TAGITDEQVVAYSWTDR | -0.0614 | 94 | 11 | 16.3 | 5.10 | 23.4 | 5.6 |
|  |  |  | BCG0906 |  |  |  | TAGITDEQVVAYSW^e^TDR | -0.0564 | 30 |  |  |  |  |  |
| 72 | *ssb* | BCGM_0055 | Rv0054 | Probable single-strand binding protein | 2 | 353 | DGEALFLR | 0.0026 | 43 | 43 | 17.3 | 5.12 | 21.8 | 5.5 |
|  |  |  | BCG0085 |  |  |  | EAAENVAESLTR | -0.0083 | 33 |  |  |  |  |  |
|  |  |  |  |  |  |  | TVIEVEVDEIGPSLR | -0.0195 | 111 |  |  |  |  |  |
|  |  |  |  |  |  |  | FTPSGAAVANFTVASTPR | -0.0225 | 140 |  |  |  |  |  |
|  |  |  |  |  |  |  | AGDTTITIVGNLTADPELR | -0.0393 | 122 |  |  |  |  |  |
| 73 | *ssb* | BCGM_0055 | Rv0054 | Probable single-strand binding protein | 2 | 323 | DGEALFLR | -0.0037 | 48 | 43 | 17.3 | 5.12 | 22.0 | 5.3 |
|  |  |  | BCG0085 |  |  |  | EAAENVAESLTR | -0.0173 | 54 |  |  |  |  |  |
|  |  |  |  |  |  |  | TVIEVEVDEIGPSLR | -0.0327 | 77 |  |  |  |  |  |
|  |  |  |  |  |  |  | FTPSGAAVANFTVASTPR | -0.0346 | 151 |  |  |  |  |  |
|  |  |  |  |  |  |  | AGDTTITIVGNLTADPELR | -0.0492 | 89 |  |  |  |  |  |
| 74 | *TB18.6* | BCGM_2141c | Rv2140c | Conserved hypothetical protein | 10 | 138 | YYVAVHAVK | -0.0627 | 47 | 23 | 18.6 | 5.41 | 19.6 | 5.3 |
|  |  |  | BCG2157c |  |  |  | WSGFPSETR | -0.0322 | 30 |  |  |  |  |  |
|  |  |  |  |  |  |  | AVIFGTYEQR | -0.0317 | 68 |  |  |  |  |  |
|  |  |  |  |  |  |  | YVGAAPPPGHGVHR | -0.0498 | 62 |  |  |  |  |  |
| 75 | *clpP2* | BCGM_2464c | Rv2460c | Probable ATP-dependent clp protease proteolytic subunit 2 | 7 | 70 | DYGIIDTVLEYR | -0.0378 | 52 | 17 | 23.5 | 4.99 | 17.6 | 5.0 |
|  |  |  | BCG2480c |  |  |  | ADIQTVC^d^LGQAASAAAVLLAAGTPGK | -0.0863 | 41 |  |  |  |  |  |
| 76 | *mpt63* | BCGM_1939c | Rv1926c | Immunogenic protein Mpt63 | 3 | 42 | GSVTPAVSQFNAR | -0.0138 | 42 | 8 | 16.5 | 4.92 | 16.5 | 4.9 |
|  |  |  | BCG1965c |  |  |  |  |  |  |  |  |  |  |  |
| 77 | *mpt63* | BCGM_1939c | Rv1926c | Immunogenic protein Mpt63 | 3 | 85 | TADGINYR | 0.0290 | 13 | 13 | 16.5 | 4.92 | 15.9 | 4.6 |
|  |  |  | BCG1965c |  |  |  | GSVTPAVSQFNAR | -0.0137 | 85 |  |  |  |  |  |
| 78 | *BCGM_0340* | BCGM_0340 | Rv0333 | Hypothetical protein | 10 | 45 | VVQDHVASVFR | -0.0000 | 45 | 8 | 13.1 | 4.46 | 16.0 | 4.4 |
|  |  |  | BCG0372 |  |  |  |  |  |  |  |  |  |  |  |
| 79 | *sseC2* | BCGM_0831c | Rv0814c | Conserved hypothetical protein SseC2 | 7 | 184 | FFAAPGSWTLR | 0.0187 | 19 | 51 | 10.1 | 4.43 | 13.7 | 4.2 |
|  |  |  | BCG0866c |  |  |  | VVDGDGQAVGGAFVR | 0.0104 | 104 |  |  |  |  |  |
|  |  |  |  |  |  |  | ALSAAGN^a^GDAVVQPSGAGIHEVDVK | 0.0387 | 103 |  |  |  |  |  |
| 80 | *sseC2* | BCGM_0831c | Rv0814c | Conserved hypothetical protein SseC2 | 7 | 42 | VVDGDGQAVGGAFVR | -0.0489 | 42 | 15 | 10.1 | 4.43 | 12.9 | 4.2 |
|  |  |  | BCG0866c |  |  |  |  |  |  |  |  |  |  |  |
|  | *groES* | BCGM_3452c | Rv3418c | 10 kDa chaperonin | 0 | 29 | ^f^EKPQEGTVVAVGPGR | -0.0556 | 21 | 15 | 10.8 | 4.62 | 12.9 | 4.2 |
|  |  |  |  |  |  |  | EKPQEGTVVAVGPGR | -0.0598 | 29 |  |  |  |  |  |
| 81 | *groES* | BCGM_3452c | Rv3418c | 10 kDa chaperonin | 0 | 61 | ^f^EKPQEGTVVAVGPGR | -0.0422 | 49 | 15 | 10.8 | 4.62 | 13.7 | 4.4 |
|  |  |  |  |  |  |  | EKPQEGTVVAVGPGR | -0.0374 | 32 |  |  |  |  |  |
| 82 | *BCGM_2928c* | BCGM_2928c | Rv2923c | Conserved hypothetical protein | 10 | 58 | TQLWVER | -0.0242 | 20 | 21 | 14.8 | 4.72 | 15.4 | 4.9 |
|  |  |  | BCG2945c |  |  |  | TQL^e^WVER | -0.0261 | 14 |  |  |  |  |  |
|  |  |  |  |  |  |  | GAQVLVGSEDVDGVFTPGELLK | -0.0845 | 58 |  |  |  |  |  |
| 83 | *mpb70* | BCGM_2880 | Rv2875 | Secreted immunogenic protein mpb70 | 3 | 216 | LPASTIDELK | -0.0383 | 64 | 14 | 19.1 | 4.75 | 14.7 | 5.1 |
|  |  |  | BCG2897 |  |  |  | ^c^QTLQGASVTVTGQGNSLK | -0.0523 | 83 |  |  |  |  |  |
|  |  |  |  |  |  |  | QTLQGASVTVTGQGNSLK | -0.0658 | 115 |  |  |  |  |  |
| 84 | *hspX* | BCGM_2035c | Rv2031c | Heat shock protein | 0 | 153 | SEFAYGSFVR | -0.0047 | 82 | 20 | 16.2 | 5.00 | 16.5 | 5.2 |
|  |  |  | BCG2050c |  |  |  | TVSLPVGADEDDIK | -0.0248 | 37 |  |  |  |  |  |
|  |  |  |  |  |  |  | TVSLPVGADEDDIKATYDK | -0.0265 | 79 |  |  |  |  |  |
| 85 | *hadC* | BCGM_0651 | Rv0637 | (3R)-hydroxyacyl-ACP dehydratase subunit | 10 | 48 | FGADIVVTR | -0.0097 | 24 | 10 | 18.9 | 5.17 | 19.0 | 5.4 |
|  |  |  |  |  |  |  | YPDYFIVGR | -0.0184 | 47 |  |  |  |  |  |
|  | *ahpE* | BCGM_2239c | Rv2238c | Peroxiredoxin AhpE | 0 | 39 | DQNQQLVTLR | -0.0164 | 39 | 6 | 16.8 | 5.24 | 19.0 | 5.4 |
|  |  |  | BCG2255c |  |  |  |  |  |  |  |  |  |  |  |
|  |  |  |  |  |  |  |  |  |  |  |  |  |  |  |

Table S1 *(continued)*

| **Spot no.** | **gene** | **BCG Moreau** | **Orthologs H37Rv / Pasteur** | **Protein Identification** | **FC** | **Protein score** | **Peptide Sequence** | **Delta^&^ (Da)** | **Ion Score** | **Cov (%)** | **Theor *M*_r_  (kDa)** | **Theor pI** | **Exp *M*_r_ (kDa)** | **Exp  pI** |
| --- | --- | --- | --- | --- | --- | --- | --- | --- | --- | --- | --- | --- | --- | --- |
| 86 | *menG* | BCGM_3880 | Rv3853 | Putative S-adenosylmethionine: 2-demethylmenaquinone | 9 | 82 | S^d^CDLQFR | 0.0033 | 23 | 11 | 16.2 | 5.00 | 20.1 | 5.4 |
|  |  |  | BCG3916 | methyltransferase |  |  | SQFAGPISTVR | -0.0002 | 81 |  |  |  |  |  |
|  | *BCGM_0048c* | BCGM_0048c | Rv0047c | Conserved hypothetical protein | 10 | 24 | LTGLLGAFR | -0.1279 | 24 | 5 | 20.4 | 10.04 | 20.1 | 5.4 |
| 87 | *TB18.6* | BCGM_2141c | Rv2140c | Conserved hypothetical protein TB18.6 | 10 | 35 | YYVAVHAVK | -0.0221 | 17 | 15 | 18.6 | 5.41 | 19.3 | 5.5 |
|  |  |  | BCG2157c |  |  |  | WSGFPSETR | -0.0106 | 10 |  |  |  |  |  |
|  |  |  |  |  |  |  | AVIFGTYEQR | -0.0107 | 35 |  |  |  |  |  |
| 88 | *BCGM_3741c* | BCGM_3741c | Rv3716c | Nucleoid-associated protein Rv3716c | 10 | 36 | GSGEVIGVTIDPK | -0.0029 | 22 | 27 | 13.3 | 4.72 | 18.5 | 5.6 |
|  |  |  | BCG3776c |  |  |  | LLEAQQQLANSEVHGQAGGGLVK | -0.0394 | 33 |  |  |  |  |  |
| 89 | *TB18.6* | BCGM_2141c | Rv2140c | conserved hypothetical protein TB18.6 | 10 | 71 | YYVAVHAVK | -0.0331 | 22 | 15 | 18.6 | 5.41 | 19.1 | 5.7 |
|  |  |  | BCG2157c |  |  |  | WSGFPSETR | 0.0126 | 40 |  |  |  |  |  |
|  |  |  |  |  |  |  | AVIFGTYEQR | 0.0135 | 52 |  |  |  |  |  |
| 90 | *canA* | BCGM_1310 | Rv1284 | Beta-carbonic anhydrase | 7 | 102 | GFVFDVATGK | -0.0246 | 23 | 24 | 18.2 | 5.48 | 19.8 | 5.8 |
|  |  |  | BCG1343 |  |  |  | NAGC^d^VVTDDVIR | -0.0275 | 30 |  |  |  |  |  |
|  |  |  |  |  |  |  | TVTDDYLANNVDYASGFK | -0.0542 | 92 |  |  |  |  |  |
| 91 | *rpiB* | BCGM_2469c | Rv2465c | Ribose-5-phosphate isomerase | 7 | 228 | IDILAEYER | -0.0213 | 17 | 48 | 17.3 | 6.14 | 19.0 | 6.8 |
|  |  |  | BCG2485c |  |  |  | EHNNAQLIGIGGR | -0.0235 | 89 |  |  |  |  |  |
|  |  |  |  |  |  |  | VYLGADHAGYELK | -0.0290 | 28 |  |  |  |  |  |
|  |  |  |  |  |  |  | QTGHEPIDC^d^GALR | -0.0365 | 33 |  |  |  |  |  |
|  |  |  |  |  |  |  | C^d^ALAWSVQTAALAR | -0.0437 | 41 |  |  |  |  |  |
|  |  |  |  |  |  |  | YDADDDYPAFC^d^IAAATR | -0.0461 | 122 |  |  |  |  |  |
| 92 | *TB15.3* | BCGM_1641 | Rv1636 | Iron-regulated universal stress protein family protein TB15.3 | 10 | 254 | LLGSVPANVSR | 0.0051 | 72 | 70 | 15.3 | 5.51 | 15.6 | 6.0 |
|  |  |  | BCG1674 |  |  |  | AKVDVLIVHTT | -0.0154 | 38 |  |  |  |  |  |
|  |  |  |  |  |  |  | AADILKDESYK | -0.0115 | 47 |  |  |  |  |  |
|  |  |  |  |  |  |  | VTGTAPIYEILHDAK | -0.0259 | 66 |  |  |  |  |  |
|  |  |  |  |  |  |  | ADLLVVGNVGLSTIAGR | -0.0237 | 64 |  |  |  |  |  |
|  |  |  |  |  |  |  | LIIASAYLPQHEDAR | -0.0208 | 83 |  |  |  |  |  |
|  |  |  |  |  |  |  | NVEERPIVGAPVDALVNLADEEK | -0.0568 | 22 |  |  |  |  |  |
| 93 | *TB15.3* | BCGM_1641 | Rv1636 | Iron-regulated universal stress protein family protein TB15.3 | 10 | 82 | LLGSVPANVSR | -0.0237 | 40 | 47 | 15.3 | 5.51 | 15.6 | 5.7 |
|  |  |  | BCG1674 |  |  |  | AKVDVLIVHTT | -0.0338 | 27 |  |  |  |  |  |
|  |  |  |  |  |  |  | VTGTAPIYEILHDAK | -0.0375 | 26 |  |  |  |  |  |
|  |  |  |  |  |  |  | ADLLVVGNVGLSTIAGR | -0.0431 | 54 |  |  |  |  |  |
|  |  |  |  |  |  |  | LIIASAYLPQHEDAR | -0.0444 | 26 |  |  |  |  |  |
| 95 | *ssb* | BCGM_0055 | Rv0054 | Probable single-strand binding protein | 2 | 27 | DGEALFLR | 0.0285 | 26 | 15 | 17.3 | 5.12 | 16.2 | 5.5 |
|  |  |  | BCG0085 |  |  |  | FTPSGAAVANFTVASTPR | 0.0072 | 22 |  |  |  |  |  |
| 96 | *hspX* | BCGM_2035c | Rv2031c | Heat shock protein | 0 | 200 | SEFAYGSFVR | 0.0088 | 82 | 31 | 16.2 | 5.00 | 16.0 | 5.4 |
|  |  |  | BCG2050c |  |  |  | TVSLPVGADEDDIK | -0.0147 | 54 |  |  |  |  |  |
|  |  |  |  |  |  |  | AELPGVDPDKDVDIMVR | -0.0223 | 21 |  |  |  |  |  |
|  |  |  |  |  |  |  | TVSLPVGADEDDIKATYDK | -0.0213 | 109 |  |  |  |  |  |
| 97 | *hrp1* | BCGM_2636c | Rv2626c | Hypoxic response protein 1 Hrp1 | 10 | 153 | RVPVISEHR | -0.0206 | 16 | 37 | 15.5 | 4.96 | 13.9 | 5.2 |
|  |  |  | BCG2653c |  |  |  | LVGIVTEADIAR | -0.0202 | 60 |  |  |  |  |  |
|  |  |  |  |  |  |  | EHDIGALPIC^d^GDDDR | -0.0431 | 88 |  |  |  |  |  |
|  |  |  |  |  |  |  | GLAAGLDPNTATAGELAR | -0.0431 | 51 |  |  |  |  |  |
| 98 | *Rv0801* | BCGM_0818 | Rv0801 | Conserved hypothetical protein | 10 | 77 | VEM^b^VTFDCSDPAK | -0.0197 | 50 | 31 | 12,6 | 4,92 | 12.9 | 5.1 |
|  |  |  |  |  |  |  | ALKVEM^b^VTFDCSDPAK | -0.0276 | 49 |  |  |  |  |  |
|  | *PPE71* | BCG_M2354c | Rv2356c | PPE family protein | 6 | 26 | AAM^b^VDPVVVAANR | 0.0459 | 26 | 2 | 44,5 | 4,17 | 12.9 | 5.1 |
|  |  |  | BCG2370c |  |  |  |  |  |  |  |  |  |  |  |
|  |  |  |  |  |  |  |  |  |  |  |  |  |  |  |

Table S1 *(continued)*

| **Spot no.** | **gene** | **BCG Moreau** | **Orthologs H37Rv / Pasteur** | **Protein Identification** | **FC** | **Protein score** | **Peptide Sequence** | **Delta^&^ (Da)** | **Ion Score** | **Cov (%)** | **Theor *M*_r_  (kDa)** | **Theor pI** | **Exp *M*_r_ (kDa)** | **Exp  pI** |
| --- | --- | --- | --- | --- | --- | --- | --- | --- | --- | --- | --- | --- | --- | --- |
| 99 | *hspX* | BCGM_2035c | Rv2031c | Heat shock protein | 0 | 64 | SEFAYGSFVR | 0.0281 | 59 | 31 | 16.2 | 5.00 | 12.9 | 5.0 |
|  |  |  | BCG2050c |  |  |  | GILTVSVAVSEGKPTEK | 0.0277 | 21 |  |  |  |  |  |
|  |  |  |  |  |  |  | TVSLPVGADEDDIKATYDK | 0.0340 | 25 |  |  |  |  |  |
| 100 | *hrp1* | BCGM_2636c | Rv2626c | Hypoxic response protein 1 Hrp1 | 10 | 114 | LVGIVTEADIAR | 0.0186 | 34 | 31 | 15.5 | 4.96 | 12.5 | 5.0 |
|  |  |  | BCG2653c |  |  |  | EHDIGALPIC^d^GDDDR | 0.0182 | 55 |  |  |  |  |  |
|  |  |  |  |  |  |  | GLAAGLDPNTATAGELAR | 0.0247 | 68 |  |  |  |  |  |
| 101 | *hspX* | BCGM_2035c | Rv2031c | Heat shock protein | 0 | 63 | SEFAYGSFVR | 0.0175 | 49 | 20 | 16.2 | 5.00 | 12.7 | 4.7 |
|  |  |  | BCG2050c |  |  |  | TVSLPVGADEDDIKATYDK | 0.0243 | 36 |  |  |  |  |  |
| 102 | *groES* | BCGM_3452c | Rv3418c | 10 kDa chaperonin | 0 | 139 | ^f^EKPQEGTVVAVGPGR | -0.0234 | 72 | 15 | 10.8 | 4.62 | 11.9 | 4.2 |
|  |  |  | BCG3488c |  |  |  | EKPQEGTVVAVGPGR | -0.0196 | 89 |  |  |  |  |  |
| 103 | *groES* | BCGM_3452c | Rv3418c | 10 kDa chaperonin | 0 | 67 | EKPQEGTVVAVGPGR | -0.0797 | 67 | 15 | 10.8 | 4.62 | 11.7 | 4.4 |
|  |  |  | BCG3488c |  |  |  |  |  |  |  |  |  |  |  |
| 104 | *groES* | BCGM_3452c | Rv3418c | 10 kDa chaperonin | 0 | 528 | YN^a^GEEYLILSAR | 0.0001 | 106 | 66 | 10.8 | 4.62 | 11.8 | 4.5 |
|  |  |  | BCG3488c |  |  |  | ^f^EKPQEGTVVAVGPGR | -0.0177 | 96 |  |  |  |  |  |
|  |  |  |  |  |  |  | EKPQEGTVVAVGPGR | -0.0105 | 141 |  |  |  |  |  |
|  |  |  |  |  |  |  | IPLDVAEGDTVIYSK | -0.0312 | 123 |  |  |  |  |  |
|  |  |  |  |  |  |  | RIPLDVAEGDTVIYSK | -0.0351 | 45 |  |  |  |  |  |
|  |  |  |  |  |  |  | ILVQANEAETTTASGLVIPDTAK | -0.0633 | 132 |  |  |  |  |  |
| 105 | *groES* | BCGM_3452c | Rv3418c | 10 kDa chaperonin | 0 | 116 | YNGEEYLILSAR | -0.0505 | 44 | 65 | 10.8 | 4.62 | 10.6 | 4.6 |
|  |  |  | BCG3488c |  |  |  | EKP^a^QEGTVVAVGPGR | -0.0410 | 49 |  |  |  |  |  |
|  |  |  |  |  |  |  | IPLDVAEGDTVIYSK | -0.0685 | 68 |  |  |  |  |  |
|  |  |  |  |  |  |  | ILVQANEAETTTASGLVIPDTAK | -0.1165 | 22 |  |  |  |  |  |
| 106 | *TB9.4* | BCGM_3219c | Rv3208A | Conserved hypothetical protein TB9.4 | 10 | 108 | FLIHTAR | 0.0586 | 15 | 45 | 9.4 | 5.02 | 11.0 | 5.3 |
|  |  |  | BCG3235c |  |  |  | IAYVEIGVADAR | 0.0348 | 60 |  |  |  |  |  |
|  |  |  |  |  |  |  | ELVFSSAQTPSEVEELVSNALR | 0.0582 | 67 |  |  |  |  |  |
| 107 | *esxJ* | BCGM_1062c | Rv1038c | ESAT-6 like protein EsxJ | 3 | 131 | FEVHAQTVEDEAR | 0.0028 | 102 | 14 | 11.0 | 5.17 | 13.3 | 5.5 |
|  |  |  | BCG1096c |  |  |  | FEVHAQTVEDEARR | -0.0084 | 52 |  |  |  |  |  |
| 108 | *ahpE* | BCGM_2239c | Rv2238c | Peroxiredoxin AhpE | 0 | 42 | DQNQQLVTLR | -0.0139 | 42 | 6 | 16.8 | 5.24 | 14.4 | 5.5 |
|  |  |  | BCG2255c |  |  |  |  |  |  |  |  |  |  |  |
| 109 | *ndkA* | BCGM_2449c | Rv2445c | Probable nucleoside diphosphate kinase | 7 | 357 | ^c^QLIGEIISR | -0.0132 | 48 | 36 | 14.5 | 5.34 | 13.9 | 5.6 |
|  |  |  | BCG2465c |  |  |  | QLIGEIISR | -0.0157 | 67 |  |  |  |  |  |
|  |  |  |  |  |  |  | GLTIAALQLR | -0.0154 | 37 |  |  |  |  |  |
|  |  |  |  |  |  |  | TLVLIKPDGIER | -0.0237 | 53 |  |  |  |  |  |
|  |  |  |  |  |  |  | ^c^QLAGGTDPVQAAAPGTIR | -0.0361 | 133 |  |  |  |  |  |
|  |  |  |  |  |  |  | QLAGGTDPVQAAAPGTIR | -0.0327 | 133 |  |  |  |  |  |
| 110 | *vapC47* | BCGM_3442 | Rv3408 | Ribonuclease VapC47 | 0 | 169 | ^f^EVGFVTASPGAVR | -0.0190 | 22 | 33 | 14.7 | 5.35 | 13.6 | 5.7 |
|  |  |  | BCG3478 |  |  |  | EVGFVTASPGAVR | -0.0144 | 62 |  |  |  |  |  |
|  |  |  |  |  |  |  | LLISEPETTELR | -0.0199 | 71 |  |  |  |  |  |
|  |  |  |  |  |  |  | TWLTAQSGQGEDAATSTLGR | -0.0490 | 83 |  |  |  |  |  |
| 111 | *BCGM_1187c* | BCGM_1187c | Rv1159A | Pterin-4-alpha-carbinolamine dehydratase | 10 | 72 | AEEVNHHPDIDIR | -0.0197 | 72 | 13 | 10.4 | 5.36 | 13.1 | 5.8 |
| 112 | *glnB* | BCGM_2923c | Rv2919c | Probable nitrogen regulatory protein P-II | 9 | 82 | VWVSPVDTIVR | -0.0054 | 47 | 20 | 12.2 | 6.09 | 13.0 | 6.1 |
|  |  |  | BCG2940c |  |  |  | VW^e^VSPVDTIVR | -0.0091 | 58 |  |  |  |  |  |
|  |  |  |  |  |  |  | IEVVVDDSIVDK | -0.0164 | 23 |  |  |  |  |  |
| 113 | *hadB* | BCGM_0650 | Rv0636 | (3R)-hydroxyacyl-ACP dehydratase subunit | 7 | 24 | FTAVVPVPNDGK | 0.0729 | 24 | 8 | 15.0 | 6.08 | 13.9 | 6.3 |
|  |  |  | BCG0685 |  |  |  |  |  |  |  |  |  |  |  |
| 114 | *cfp17, garA* | BCGM_1838 | Rv1827 | Glycogen accumulation regulator GarA | 10 | 51 | ^f^EPVDSAVLA^a^NGDEVQIGK | -0.1078 | 12 | 11 | 17.2 | 4.29 | 11.5 | 6.1 |
|  |  |  | BCG1862 |  |  |  | EPVDSAVLA^a^NGDEVQIGK | -0.1050 | 51 |  |  |  |  |  |

Table S1 *(continued)*

| **Spot no.** | **gene** | **BCG Moreau** | **Orthologs H37Rv / Pasteur** | **Protein Identification** | **FC** | **Protein score** | **Peptide Sequence** | **Delta^&^ (Da)** | **Ion Score** | **Cov (%)** | **Theor *M*_r_  (kDa)** | **Theor pI** | **Exp *M*_r_ (kDa)** | **Exp  pI** |
| --- | --- | --- | --- | --- | --- | --- | --- | --- | --- | --- | --- | --- | --- | --- |
| 115 | *BCGM_2748* | BCGM_2748 | Rv2749 | Conserved hypothetical protein | 10 | 59 | PVVVVATLTAKPESVDTVR | -0.0381 | 59 | 18 | 11.1 | 5.19 | 10.6 | 5.6 |
|  |  |  | BCG2765 |  |  |  |  |  |  |  |  |  |  |  |
| 116 | *groES* | BCGM_3452c | Rv3418c | 10 kDa chaperonin | 0 | 437 | YNGEEYLILSAR | -0.0892 | 103 | 73 | 10.8 | 4.62 | 11.7 | 4.7 |
|  |  |  | BCG3488c |  |  |  | ^f^EKPQEGTVVAVGPGR | -0.1040 | 40 |  |  |  |  |  |
|  |  |  |  |  |  |  | EKPQEGTVVAVGPGR | -0.1048 | 135 |  |  |  |  |  |
|  |  |  |  |  |  |  | RIPLDVAEGDTVIYSK | -0.1120 | 88 |  |  |  |  |  |
|  |  |  |  |  |  |  | YGGTEIKYN^a^GEEYLILSAR | -0.1282 | 160 |  |  |  |  |  |
|  |  |  |  |  |  |  | ILVQANEAETTTASGLVIPDTAK | -0.1497 | 25 |  |  |  |  |  |
| 117 | *ppiA* | BCGM_0009 | Rv0009 | Probable iron-regulated peptidyl-prolyl cis-trans isomerase A | 2 | 238 | IALFGNHAPK | -0.0913 | 23 | 35 | 19.2 | 5.80 | 22.5 | 6.9 |
|  |  |  | BCG0009 |  |  |  | HTIFGEVIDAESQR | -0.1148 | 95 |  |  |  |  |  |
|  |  |  |  |  |  |  | VIQGFMIQGGDPTGTGR | -0.1258 | 31 |  |  |  |  |  |
|  |  |  |  |  |  |  | DYSTQNASGGPSGPFYDGAVFHR | -0.1525 | 159 |  |  |  |  |  |
| 118 | *echA3* | BCGM_0644c | Rv0632c | Probable enoyl-CoA hydratase | 1 | 100 | GGFELAYR | -0.0478 | 41 | 19 | 24.3 | 5.52 | 27.5 | 5.8 |
|  |  |  | BCG0679c |  |  |  | VFSGGFDLK | -0.0678 | 58 |  |  |  |  |  |
|  |  |  |  |  |  |  | EFAGLNQHAHAATK | -0.1174 | 29 |  |  |  |  |  |
|  |  |  |  |  |  |  | ILTSGEVQPAIDMLR | -0.1089 | 31 |  |  |  |  |  |
| 119 | *ahpC* | BCGM_2431 | Rv2428 | Alkyl hydroperoxide reductase c protein | 0 | 647 | NVDEVLR | -0.0492 | 36 | 58 | 21.5 | 4.50 | 26.1 | 4.5 |
|  |  |  | BCG2447 |  |  |  | VVFFWPK | -0.0825 | 53 |  |  |  |  |  |
|  |  |  |  |  |  |  | TLPFPMLSDIKR | -0.1108 | 75 |  |  |  |  |  |
|  |  |  |  |  |  |  | ELSQAAGVLNADGVADR | -0.1350 | 201 |  |  |  |  |  |
|  |  |  |  |  |  |  | DFTFVC^d^PTEIAAFSK | -0.1678 | 68 |  |  |  |  |  |
|  |  |  |  |  |  |  | ^c^QPGDYFTTITSDEHPGK | -0.1532 | 115 |  |  |  |  |  |
|  |  |  |  |  |  |  | VLDALQ^a^SDELC^d^AC^d^NWR | -0.1394 | 95 |  |  |  |  |  |
|  |  |  |  |  |  |  | VTFIVDPNNEIQFVSATAGSVGR | -0.1772 | 163 |  |  |  |  |  |
| 120 | *ahpC* | BCGM_2431 | Rv2428 | Alkyl hydroperoxide reductase c protein | 0 | 351 | VVFFWPK | -0.0866 | 35 | 47 | 21.5 | 4.50 | 26.4 | 4.4 |
|  |  |  | BCG2447 |  |  |  | TLPFPMLSDIKR | -0.1190 | 53 |  |  |  |  |  |
|  |  |  |  |  |  |  | ^f^ELSQAAGVLNADGVADR | -0.1384 | 31 |  |  |  |  |  |
|  |  |  |  |  |  |  | ELSQAAGVLNADGVADR | -0.1411 | 137 |  |  |  |  |  |
|  |  |  |  |  |  |  | ^c^QPGDYFTTITSDEHPGK | -0.1632 | 83 |  |  |  |  |  |
|  |  |  |  |  |  |  | VLDAL^a^QSDEL^d^CA^d^CNWR | -0.1503 | 37 |  |  |  |  |  |
|  |  |  |  |  |  |  | VTFIVDPNNEIQFVSATAGSVGR | -0.1860 | 106 |  |  |  |  |  |
| 121 | *ahpC* | BCGM_2431 | Rv2428 | Alkyl hydroperoxide reductase c protein | 0 | 293 | NVDEVLR | -0.0445 | 33 | 38 | 21.5 | 4.50 | 26.4 | 4.3 |
|  |  |  | BCG2447 |  |  |  | VVFFWPK | -0.0848 | 38 |  |  |  |  |  |
|  |  |  |  |  |  |  | TLPFPMLSDIKR | -0.1058 | 75 |  |  |  |  |  |
|  |  |  |  |  |  |  | ELSQAAGVLNADGVADR | -0.1279 | 109 |  |  |  |  |  |
|  |  |  |  |  |  |  | ^c^QPGDYFTTITSDEHPGK | -0.1558 | 109 |  |  |  |  |  |
|  |  |  |  |  |  |  | VLDALQSDELC^d^AC^d^NWR | -0.1401 | 43 |  |  |  |  |  |
| 122 | *gpm1* | BCGM_0498 | Rv0489 | Probable phosphoglycerate mutase 1 | 7 | 118 | SYDTPPPPIER | -0.0993 | 38 | 26 | 27.2 | 5.38 | 29.1 | 5.7 |
|  |  |  | BCG0530 |  |  |  | TVLIVAHGNSLR | -0.0970 | 32 |  |  |  |  |  |
|  |  |  |  |  |  |  | YGEEQFMAWR | -0.0975 | 62 |  |  |  |  |  |
|  |  |  |  |  |  |  | FLPYFTDVIVGDLR | -0.1265 | 33 |  |  |  |  |  |
|  |  |  |  |  |  |  | YADIGGGPLTEC^d^LADVVAR | -0.1416 | 39 |  |  |  |  |  |
| 123 | *echA20* | BCGM_3578 | Rv3550 | Probable enoyl-coa hydratase | 1 | 127 | GC^d^FAAFR | -0.0351 | 37 | 14 | 26.3 | 5.41 | 28.8 | 5.6 |
|  |  |  | BCG3614 |  |  |  | LVPQHLMR | -0.0670 | 19 |  |  |  |  |  |
|  |  |  |  |  |  |  | EALNFIDVQR | -0.0871 | 81 |  |  |  |  |  |
|  |  |  |  |  |  |  | TEGFTALIDANR | -0.0927 | 50 |  |  |  |  |  |
|  |  |  |  |  |  |  |  |  |  |  |  |  |  |  |

Table S1 *(continued)*

| **Spot no.** | **gene** | **BCG Moreau** | **Orthologs H37Rv / Pasteur** | **Protein Identification** | **FC** | **Protein score** | **Peptide Sequence** | **Delta^&^ (Da)** | **Ion Score** | **Cov (%)** | **Theor *M*_r_  (kDa)** | **Theor pI** | **Exp *M*_r_ (kDa)** | **Exp  pI** |
| --- | --- | --- | --- | --- | --- | --- | --- | --- | --- | --- | --- | --- | --- | --- |
|  | *tuf* | BCGM_0699 | Rv0685 | Probable elongation factor TU tuf (EF-TU) | 2 | 122 | LIQPVAMDEGLR | -0.1012 | 38 | 12 | 43.6 | 5.28 | 28.8 | 5.6 |
|  |  |  | BCG0734 |  |  |  | LLDQGQAGDNVGLLLR | -0.1296 | 83 |  |  |  |  |  |
|  |  |  |  |  |  |  | GVINVNEEVEIVGIRPSTTK | -0.1492 | 44 |  |  |  |  |  |
| 124 | *prcB* | BCGM_2111c | Rv2110c | Proteasome (beta subunit) | 7 | 310 | YPGGVVMAGDR | -0.0940 | 18 | 27 | 30.3 | 4.65 | 28.5 | 4.8 |
|  |  |  | BCG2127c |  |  |  | YPGGVVMAGDRR | -0.1214 | 13 |  |  |  |  |  |
|  |  |  |  |  |  |  | LYAVELEHYEK | -0.1446 | 77 |  |  |  |  |  |
|  |  |  |  |  |  |  | LYSQVTDGDSGLR | -0.1294 | 30 |  |  |  |  |  |
|  |  |  |  |  |  |  | KLYSQVTDGDSGLR | -0.1415 | 33 |  |  |  |  |  |
|  |  |  |  |  |  |  | GIFPTAVIIDADGAVDVPESR | -0.1863 | 84 |  |  |  |  |  |
|  |  |  |  |  |  |  | VAVEALYDAADDDSATGGPDLVR | -0.1925 | 181 |  |  |  |  |  |
|  | *BCGM_3723* | BCGM_3723 | Rv3699 | Conserved hypothetical protein | 10 | 56 | GAFPAELEVKPNEVDEDELR | -0.1880 | 56 | 8 | 25.0 | 4.69 | 28.5 | 4.8 |
|  |  |  | BCG3758 |  |  |  |  |  |  |  |  |  |  |  |
| 125 | *BCGM_3723* | BCGM_3723 | Rv3699 | Conserved hypothetical protein | 10 | 572 | YLSSVHR | -0.0684 | 19 | 40 | 25.0 | 4.69 | 28.6 | 4.7 |
|  |  |  | BCG3758 |  |  |  | FPAYLLTAHK | -0.1313 | 59 |  |  |  |  |  |
|  |  |  |  |  |  |  | VKFPAYLLTAHK | -0.1513 | 74 |  |  |  |  |  |
|  |  |  |  |  |  |  | ^g^TDEVMDWDSAYR | -0.1512 | 81 |  |  |  |  |  |
|  |  |  |  |  |  |  | ^g^TDEVMD^e^WDSAYR | -0.1665 | 25 |  |  |  |  |  |
|  |  |  |  |  |  |  | FSTVIDSTLFHSLPVDSR | -0.1809 | 95 |  |  |  |  |  |
|  |  |  |  |  |  |  | GAFPAELEVKPNEVDEDELR | -0.1863 | 164 |  |  |  |  |  |
|  |  |  |  |  |  |  | GLTTASFVQADITEFAAYPAGSAGR | -0.1997 | 209 |  |  |  |  |  |
| 126 | *fbpA* | BCGM_3830c | Rv3804c | Secreted antigen 85-A | 1 | 198 | FLEGFVR | -0.0528 | 41 | 21 | 35.7 | 6.08 | 31.1 | 5.6 |
|  |  |  | BCG3866c |  |  |  | EDPAWQR | -0.0636 | 28 |  |  |  |  |  |
|  |  |  |  |  |  |  | WETFLTSELPGWLQANR | -0.1131 | 48 |  |  |  |  |  |
|  |  |  |  |  |  |  | VQFQSGGANSPALYLLDGLR | -0.1430 | 146 |  |  |  |  |  |
|  |  |  |  |  |  |  | VWVY^d^CG^a^NGKPSDLGGNNLPAK | -0.1461 | 11 |  |  |  |  |  |
| 127 | *fbpB* | BCGM_1898c | Rv1886c | Secreted antigen 85-B | 1 | 283 | AADMWGPSSDPAWER | -0.1223 | 107 | 16 | 34.5 | 5.62 | 31.4 | 5.0 |
|  |  |  | BCG1923c |  |  |  | AADM^e^WGPSSDPAWER | -0.1355 | 21 |  |  |  |  |  |
|  |  |  |  |  |  |  | WETLLTSELPQWLSANR | -0.1405 | 76 |  |  |  |  |  |
|  |  |  |  |  |  |  | VQFQ^a^SGGNNSPAVYLLDGLR | -0.1296 | 146 |  |  |  |  |  |
| 128 | *fixA* | BCGM_3036c | Rv3029c | Probable electron transfer flavoprotein (beta-subunit) | 7 | 583 | AVEEALQIR | -0.0658 | 61 | 30 | 28.1 | 4.66 | 30.6 | 4.8 |
|  |  |  | BCG3052c |  |  |  | ^c^QVPDTWSER | -0.0705 | 58 |  |  |  |  |  |
|  |  |  |  |  |  |  | QVPDTWSER | -0.0750 | 49 |  |  |  |  |  |
|  |  |  |  |  |  |  | KLTDGDFTLDR | -0.0882 | 76 |  |  |  |  |  |
|  |  |  |  |  |  |  | EAADAVLDEINER | -0.1008 | 130 |  |  |  |  |  |
|  |  |  |  |  |  |  | EAADGIEGSVTVLTAGPER | -0.1218 | 99 |  |  |  |  |  |
|  |  |  |  |  |  |  | DDGMHGSDVIQTGWALAR | -0.1232 | 87 |  |  |  |  |  |
|  |  |  |  |  |  |  | DDGM^e^HGSDVIQTGWALAR | -0.1324 | 22 |  |  |  |  |  |
|  |  |  |  |  |  |  | ^f^EKEAADGIEGSVTVLTAGPER | -0.1211 | 35 |  |  |  |  |  |
|  |  |  |  |  |  |  | EKEAADGIEGSVTVLTAGPER | -0.1316 | 172 |  |  |  |  |  |
| 129 | *fixA* | BCGM_3036c | Rv3029c | Probable electron transfer flavoprotein (beta-subunit) | 7 | 490 | AVEEALQIR | -0.0679 | 69 | 30 | 28.1 | 4.66 | 31.0 | 4.7 |
|  |  |  | BCG3052c |  |  |  | ^c^QVPDTWSER | -0.0750 | 45 |  |  |  |  |  |
|  |  |  |  |  |  |  | QVPDTWSER | -0.0792 | 55 |  |  |  |  |  |
|  |  |  |  |  |  |  | KLTDGDFTLDR | -0.0918 | 53 |  |  |  |  |  |
|  |  |  |  |  |  |  | EAADAVLDEINER | -0.1033 | 130 |  |  |  |  |  |
|  |  |  |  |  |  |  | EAADGIEGSVTVLTAGPER | -0.1262 | 74 |  |  |  |  |  |
|  |  |  |  |  |  |  | DDGMHGSDVIQTGWALAR | -0.1300 | 63 |  |  |  |  |  |
|  |  |  |  |  |  |  | EKEAADGIEGSVTVLTAGPER | -0.1336 | 164 |  |  |  |  |  |

Table S1 *(continued)*

| **Spot no.** | **gene** | **BCG Moreau** | **Orthologs H37Rv / Pasteur** | **Protein Identification** | **FC** | **Protein score** | **Peptide Sequence** | **Delta^&^ (Da)** | **Ion Score** | **Cov (%)** | **Theor *M*_r_  (kDa)** | **Theor pI** | **Exp *M*_r_ (kDa)** | **Exp  pI** |
| --- | --- | --- | --- | --- | --- | --- | --- | --- | --- | --- | --- | --- | --- | --- |
| 130 | *fixA* | BCGM_3036c | Rv3029c | Probable electron transfer flavoprotein (beta-subunit) | 7 | 160 | AVEEALQIR | -0.0736 | 49 | 23 | 28.1 | 4.66 | 31.0 | 4.6 |
|  |  |  | BCG3052c |  |  |  | ^c^QVPDTWSER | -0.0691 | 19 |  |  |  |  |  |
|  |  |  |  |  |  |  | QVPDTWSER | -0.0814 | 18 |  |  |  |  |  |
|  |  |  |  |  |  |  | KLTDGDFTLDR | -0.0924 | 23 |  |  |  |  |  |
|  |  |  |  |  |  |  | EAADAVLDEINER | -0.1177 | 74 |  |  |  |  |  |
|  |  |  |  |  |  |  | EAADGIEGSVTVLTAGPER | -0.1435 | 17 |  |  |  |  |  |
|  |  |  |  |  |  |  | EKEAADGIEGSVTVLTAGPER | -0.1429 | 83 |  |  |  |  |  |
| 131 | *TB27.3* | BCGM_0588 | Rv0577 | Putative glyoxylase CFP32 | 10 | 180 | AAAAGGQVIAEPADIPSVGR | -0.1836 | 111 | 22 | 27.3 | 4.41 | 31.1 | 4.4 |
|  | *cfp32* |  | BCG0622 |  |  |  | VVPGGGQVMMPAFDIGDAGR | -0.1958 | 53 |  |  |  |  |  |
|  |  |  |  |  |  |  | MSFITDPTGAAVGLWQANR | -0.2100 | 62 |  |  |  |  |  |
| 132 | *pepA* | BCGM_0129 | Rv0125 | Probable serine protease | 7 | 89 | TQDVAVLQLR | -0.0959 | 87 | 20 | 34.9 | 5.04 | 31.8 | 4.6 |
|  |  |  | BCG0159 |  |  |  | SGGGSPTVHIGPTAFLGLGVVDN^a^NGNGAR | -0.1745 | 26 |  |  |  |  |  |
|  |  |  |  |  |  |  | GAGGLPSAAIGGGVAVGEPVVAMGN^a^SGGQGGTPR | -0.1687 | 11 |  |  |  |  |  |
| 133 | *fixB* | BCGM_3035c | Rv3028c | Probable electron transfer flavoprotein (alpha-subunit) | 7 | 641 | VSAELITAAR | -0.0505 | 42 | 48 | 31.7 | 4.71 | 33.0 | 4.7 |
|  |  |  | BCG3051c |  |  |  | IGSGLLVDVVDVR | -0.0709 | 134 |  |  |  |  |  |
|  |  |  |  |  |  |  | AEVLVLVEHAEGALKK | -0.1007 | 147 |  |  |  |  |  |
|  |  |  |  |  |  |  | AAVDSGYYPGQ^a^FQVGQTGK | -0.1078 | 65 |  |  |  |  |  |
|  |  |  |  |  |  |  | TVSPQLYIALGISGAIQHR | -0.1045 | 106 |  |  |  |  |  |
|  |  |  |  |  |  |  | EPAVAGDRPELTEATIVVAGGR | -0.1109 | 34 |  |  |  |  |  |
|  |  |  |  |  |  |  | GVGSAENFSVVEALADSLGAAVGASR | -0.1093 | 64 |  |  |  |  |  |
|  |  |  |  |  |  |  | AGAVEAEPAAGAGEQVSVEVPAAAENAAR | -0.1119 | 200 |  |  |  |  |  |
|  | *BCGM_1910c* | BCGM_1910c | Rv1896c | Putative S-adenosyl-L-met-dependent methyltransferase | 10 | 70 | FFDDFFN^d^CADEAGIR | -0.0886 | 70 | 4 | 33.2 | 4.76 | 33.0 | 4.7 |
|  |  |  |  |  |  |  |  |  |  |  |  |  |  |  |
| 134 | *fixB* | BCGM_3035c | Rv3028c | Probable electron transfer flavoprotein (alpha-subunit) | 7 | 496 | VSAELITAAR | -0.0686 | 31 | 48 | 31.7 | 4.71 | 32.5 | 4.8 |
|  |  |  | BCG3051c |  |  |  | IGSGLLVDVVDVR | -0.0922 | 127 |  |  |  |  |  |
|  |  |  |  |  |  |  | AEVLVLVEHAEGALKK | -0.1222 | 73 |  |  |  |  |  |
|  |  |  |  |  |  |  | AAVDSGYYPGQFQ^a^VGQTGK | -0.1286 | 26 |  |  |  |  |  |
|  |  |  |  |  |  |  | TVSPQLYIALGISGAIQHR | -0.1239 | 125 |  |  |  |  |  |
|  |  |  |  |  |  |  | EPAVAGDRPELTEATIVVAGGR | -0.1275 | 29 |  |  |  |  |  |
|  |  |  |  |  |  |  | GVGSAENFSVVEALADSLGAAVGASR | -0.1369 | 53 |  |  |  |  |  |
|  |  |  |  |  |  |  | AGAVEAEPAAGAGEQVSVEVPAAAENAAR | -0.1368 | 181 |  |  |  |  |  |
| 135 | *cysA2* | BCGM_0832c | Rv0815c | Probable thiosulfate sulfurtransferase | 7 | 161 | SSHTWFVLR | -0.0618 | 70 | 16 | 31.0 | 5.14 | 32.7 | 5.3 |
|  |  |  | BCG0867c |  |  |  | SSHT^e^WFVLR | -0.0675 | 17 |  |  |  |  |  |
|  |  |  |  |  |  |  | AFRDEVLAAINVK | -0.0836 | 70 |  |  |  |  |  |
|  |  |  |  |  |  |  | VVFVEVDEDTSAYDR | -0.0931 | 31 |  |  |  |  |  |
|  |  |  |  |  |  |  | VVFVEVDEDTSAYDRDHIAGAIK | -0.1368 | 54 |  |  |  |  |  |
| 136 | *htdY* | BCGM_3426c | Rv3389c | Probable 3-hydroxyacyl-thioester dehydratase HtdY | 7 | 375 | GQGGFGGAR | -0.0479 | 14 | 39 | 30.3 | 5.17 | 34.2 | 5.5 |
|  |  |  | BCG3602 |  |  |  | IDMPTREDQALIYR | -0.1258 | 23 |  |  |  |  |  |
|  |  |  |  |  |  |  | VGTFNPAALLHGSQGIR | -0.1316 | 112 |  |  |  |  |  |
|  |  |  |  |  |  |  | ALVAELGGGVAANITSIAAR | -0.1396 | 151 |  |  |  |  |  |
|  |  |  |  |  |  |  | GERPAAPEFPDRHPDAR | -0.1497 | 34 |  |  |  |  |  |
|  |  |  |  |  |  |  | FTKPVFPGETLSTVIWR | -0.1527 | 112 |  |  |  |  |  |
|  |  |  |  |  |  |  | GC^d^DPESGSLVAETLTTLVLR | -0.1626 | 45 |  |  |  |  |  |
|  |  |  |  |  |  |  |  |  |  |  |  |  |  |  |
|  |  |  |  |  |  |  |  |  |  |  |  |  |  |  |
|  |  |  |  |  |  |  |  |  |  |  |  |  |  |  |
|  |  |  |  |  |  |  |  |  |  |  |  |  |  |  |

Table S1 *(continued)*

| **Spot no.** | **gene** | **BCG Moreau** | **Orthologs H37Rv / Pasteur** | **Protein Identification** | **FC** | **Protein score** | **Peptide Sequence** | **Delta^&^ (Da)** | **Ion Score** | **Cov (%)** | **Theor *M*_r_  (kDa)** | **Theor pI** | **Exp *M*_r_ (kDa)** | **Exp  pI** |
| --- | --- | --- | --- | --- | --- | --- | --- | --- | --- | --- | --- | --- | --- | --- |
| 137 | *BCGM_2977* | BCGM_2977 | Rv2971 | Probable oxidoreductase | 7 | 546 | TPAQVLLR | -0.0587 | 63 | 37 | 30.4 | 4.76 | 34.5 | 4.9 |
|  |  |  | BCG2993 |  |  |  | LATPDQGFTR | -0.0727 | 47 |  |  |  |  |  |
|  |  |  |  |  |  |  | AVSAALEIGC^d^R | -0.0774 | 80 |  |  |  |  |  |
|  |  |  |  |  |  |  | WNLQLGNAVVVR | -0.0847 | 121 |  |  |  |  |  |
|  |  |  |  |  |  |  | YVDAWGGMIQSR | -0.0868 | 84 |  |  |  |  |  |
|  |  |  |  |  |  |  | YVDA^e^WGGMIQSR | -0.1036 | 17 |  |  |  |  |  |
|  |  |  |  |  |  |  | LIDTAYAYGNEAAVGR | -0.1045 | 151 |  |  |  |  |  |
|  |  |  |  |  |  |  | LLDNPTVTSIASEYVK | -0.1214 | 72 |  |  |  |  |  |
|  |  |  |  |  |  |  | ANAQHTVVTQ^a^SYC^d^PLALGR | -0.1124 | 51 |  |  |  |  |  |
|  |  |  |  |  |  |  | KANAQHTVVTQ^a^SYC^d^PLALGR | -0.1188 | 53 |  |  |  |  |  |
| 138 | *nusG* | BCGM_0653 | Rv0639 | Probable transcription antitermination protein NusG | 2 | 341 | VLVSIFGR | -0.0473 | 50 | 25 | 25.4 | 4.70 | 35.0 | 4.7 |
|  |  |  | BCG0688 |  |  |  | VLPGYILVR | -0.0593 | 90 |  |  |  |  |  |
|  |  |  |  |  |  |  | KVLPGYILVR | -0.0689 | 55 |  |  |  |  |  |
|  |  |  |  |  |  |  | MDLTDDSWAAVR | -0.0863 | 102 |  |  |  |  |  |
|  |  |  |  |  |  |  | MDLTDDS^e^WAAVR | -0.1010 | 21 |  |  |  |  |  |
|  |  |  |  |  |  |  | ETPVELTFGQVSK | -0.1065 | 56 |  |  |  |  |  |
|  |  |  |  |  |  |  | SKPGDWYVVHSYAGYENK | -0.1269 | 97 |  |  |  |  |  |
| 139 | *grpE* | BCGM_0358 | Rv0351 | Probable GrpE protein (hsp-70 cofactor) | 0 | 95 | VQADFANYR | -0.1020 | 58 | 17 | 24.5 | 4.39 | 33.1 | 4.4 |
|  |  |  | BCG0390 |  |  |  | VAELTADLQR | -0.1072 | 17 |  |  |  |  |  |
|  |  |  |  |  |  |  | RIDPETGEVR | -0.1245 | 14 |  |  |  |  |  |
|  |  |  |  |  |  |  | ^c^QGYQLGEQVLR | -0.1209 | 30 |  |  |  |  |  |
|  |  |  |  |  |  |  | QGYQ^a^LGEQVLR | -0.1147 | 54 |  |  |  |  |  |
| 140 | *grpE* | BCGM_0358 | Rv0351 | Probable GrpE protein (Hsp70 cofactor) | 0 | 85 | VQADFANYR | -0.1000 | 35 | 8 | 24.5 | 4.39 | 33.5 | 4.3 |
|  |  |  | BCG0390 |  |  |  | ^c^QGYQLGEQVLR | -0.1231 | 55 |  |  |  |  |  |
|  |  |  |  |  |  |  | QGY^a^QLGEQVLR | -0.1194 | 39 |  |  |  |  |  |
| 141 | *mdh* | BCGM_1267 | Rv1240 | Probable malate dehydrogenase | 7 | 520 | ALNAVAADDVR | -0.0656 | 69 | 40 | 34.3 | 4.65 | 37.2 | 4.8 |
|  |  |  | BCG1300 |  |  |  | GASSAASAASATIDAAR | -0.0807 | 44 |  |  |  |  |  |
|  |  |  |  |  |  |  | SDLLEAN^a^GAIFTAQGK | -0.1024 | 93 |  |  |  |  |  |
|  |  |  |  |  |  |  | IFDGVSLALLVGARPR | -0.1009 | 21 |  |  |  |  |  |
|  |  |  |  |  |  |  | LASGSLLGPDRPIELR | -0.1028 | 56 |  |  |  |  |  |
|  |  |  |  |  |  |  | VAVTGAAGQIGYSLLFR | -0.1024 | 123 |  |  |  |  |  |
|  |  |  |  |  |  |  | GGNWTIVSGLEIDEFSR | -0.1099 | 152 |  |  |  |  |  |
|  |  |  |  |  |  |  | VGVTGNPANTNALIAMTNAPDIPR | -0.1182 | 116 |  |  |  |  |  |
| 142 | *trpS* | BCGM_3375c | Rv3336c | Probable tryptophanyl-tRNA synthetase TrpS | 2 | 107 | DTAEAVVEFVNPIQAR | -0.1343 | 80 | 16 | 36.2 | 5.08 | 38.0 | 5.4 |
|  |  |  | BCG3407c |  |  |  | TLITAAQYLALGIDPGR | -0.1415 | 20 |  |  |  |  |  |
|  |  |  |  |  |  |  | VDELTADPAELEAVLAAGAQR | -0.1587 | 49 |  |  |  |  |  |
|  | *pstS3* | BCGM_0945 | Rv0928 | Periplasmic phosphate-binding lipoprotein PstS3 | 3 | 50 | DFTLPGER | -0.0655 | 37 | 15 | 37.9 | 5.76 | 38.0 | 5.4 |
|  |  |  |  |  |  |  | SFQGGVGEGAR | -0.0752 | 19 |  |  |  |  |  |
|  |  |  |  |  |  |  | RPGSYPIVLATYEIV^d^CSK | -0.1502 | 15 |  |  |  |  |  |
|  |  |  |  |  |  |  | IF^a^NGSITQW^a^NNPAIQALNR | -0.1436 | 33 |  |  |  |  |  |
| 143 | *adhC* | BCGM_3053 | Rv3045 | Probable NADP-dependent alcohol dehydrogenase | 7 | 329 | HWNAGANTR | -0.0869 | 35 | 27 | 37.0 | 5.11 | 39.1 | 5.5 |
|  |  |  | BCG3069 |  |  |  | VGVGC^d^FVDSC^d^R | -0.1093 | 40 |  |  |  |  |  |
|  |  |  |  |  |  |  | SYYATADPDTFR | -0.1306 | 45 |  |  |  |  |  |
|  |  |  |  |  |  |  | VAIIGLGGLGHMGVK | -0.1359 | 66 |  |  |  |  |  |
|  |  |  |  |  |  |  | RDPGPHDVAIDIK | -0.1284 | 38 |  |  |  |  |  |
|  |  |  |  |  |  |  | FAGIC^d^HSDIHTVK | -0.1450 | 76 |  |  |  |  |  |
|  |  |  |  |  |  |  | SYYATADPDTFRK | -0.1438 | 20 |  |  |  |  |  |
|  |  |  |  |  |  |  | DGQPTQGGYSEAIVVDENYVLR | -0.1870 | 163 |  |  |  |  |  |

Table S1 *(continued)*

| **Spot no.** | **gene** | **BCG Moreau** | **Orthologs H37Rv / Pasteur** | **Protein Identification** | **FC** | **Protein score** | **Peptide Sequence** | **Delta^&^ (Da)** | **Ion Score** | **Cov (%)** | **Theor *M*_r_  (kDa)** | **Theor pI** | **Exp *M*_r_ (kDa)** | **Exp  pI** |
| --- | --- | --- | --- | --- | --- | --- | --- | --- | --- | --- | --- | --- | --- | --- |
| 144 | *gap* | BCGM_1465 | Rv1436 | Probable glyceraldehyde-3-phosphate dehydrogenase | 7 | 435 | FDSILGR | -0.0578 | 45 | 24 | 35.9 | 5.19 | 40.1 | 5.5 |
|  |  |  | BCG1497 |  |  |  | VGI^a^NGFGR | -0.0559 | 36 |  |  |  |  |  |
|  |  |  |  |  |  |  | GKLDGYALR | -0.0832 | 50 |  |  |  |  |  |
|  |  |  |  |  |  |  | VLDDEFGIVK | -0.1208 | 52 |  |  |  |  |  |
|  |  |  |  |  |  |  | VVSWYDNEWGYSNR | -0.1482 | 92 |  |  |  |  |  |
|  |  |  |  |  |  |  | VVSW^e^YDNEWGYSNR | -0.1506 | 29 |  |  |  |  |  |
|  |  |  |  |  |  |  | LPC^d^DVGLEGDDTIVVGR | -0.1592 | 171 |  |  |  |  |  |
|  |  |  |  |  |  |  | VPIPTGSVTDLTVDLSTR | -0.1708 | 114 |  |  |  |  |  |
| 145 | *fadA3* | BCGM_1098c | Rv1074c | Probable beta-ketoacyl CoA thiolase fadA3 | 1 | 180 | NPLFDGAQER | 0.0100 | 40 | 26 | 42.6 | 4.92 | 42.5 | 5.2 |
|  |  |  | BCG1132c |  |  |  | AEEAIK^a^NGFFER | 0.0185 | 18 |  |  |  |  |  |
|  |  |  |  |  |  |  | SAAAAAGADEWHDPR | 0.0050 | 27 |  |  |  |  |  |
|  |  |  |  |  |  |  | AGEGDAFISAGVETVSR | 0.0151 | 52 |  |  |  |  |  |
|  |  |  |  |  |  |  | VVAVALGYDFLPGTTVNR | 0.0243 | 107 |  |  |  |  |  |
|  |  |  |  |  |  |  | GNSDSWPDTKNPLFDGAQER | 0.0284 | 34 |  |  |  |  |  |
|  |  |  |  |  |  |  | EITPVTLPDGTTVSTDDGPRPGTTYEK | 0.0341 | 33 |  |  |  |  |  |
|  | *ino1* | BCGM_0047c | Rv0046c | Myo-inositol-1-phosphate synthase INO1 | 7 | 26 | SPPEQLPDDIAR | 0.0084 | 18 | 8 | 39.9 | 4.96 | 42.5 | 5.2 |
|  |  |  |  |  |  |  | LEVWDSPNSAGVIIDAVR | 0.0162 | 26 |  |  |  |  |  |
| 146 | *fadA3* | BCGM_1098c | Rv1074c | Probable beta-ketoacyl CoA thiolase fadA3 | 1 | 182 | NPLFDGAQER | 0.0233 | 65 | 26 | 42.6 | 4.92 | 42.7 | 5.1 |
|  |  |  | BCG1132c |  |  |  | AEEAIKN^a^GFFER | 0.0260 | 33 |  |  |  |  |  |
|  |  |  |  |  |  |  | SAAAAAGADEWHDPR | 0.0242 | 27 |  |  |  |  |  |
|  |  |  |  |  |  |  | AGEGDAFISAGVETVSR | 0.0340 | 80 |  |  |  |  |  |
|  |  |  |  |  |  |  | VVAVALGYDFLPGTTVNR | 0.0406 | 49 |  |  |  |  |  |
|  |  |  |  |  |  |  | GNSDSWPDTKNPLFDGAQER | 0.0555 | 30 |  |  |  |  |  |
|  |  |  |  |  |  |  | EITPVTLPDGTTVSTDDGPRPGTTYEK | 0.0724 | 31 |  |  |  |  |  |
|  | *ino1* | BCGM_0047c | Rv0046c | Myo-inositol-1-phosphate synthase INO1 | 7 | 28 | SPPEQLPDDIAR | 0.0262 | 28 | 6 | 39.9 | 4.96 | 42.7 | 5.1 |
|  |  |  |  |  |  |  | IADVAPTNVIVQR | 0.0158 | 18 |  |  |  |  |  |
| 147 | *apa* | BCGM_1871 | Rv1860 | Alanine and proline rich secreted protein | 3 | 60 | TTGDPPFPGQPPPVANDTR | 0.0395 | 60 | 5 | 32.7 | 4.93 | 41.7 | 4.3 |
|  |  |  | BCG1896 |  |  |  |  |  |  |  |  |  |  |  |
| 148 | *apa* | BCGM_1871 | Rv1860 | Alanine and proline rich secreted protein | 3 | 34 | TTGDPPFPGQPPPVANDTR | 0.0331 | 34 | 5 | 32.7 | 4.93 | 41.8 | 4.2 |
|  |  |  | BCG1896 |  |  |  |  |  |  |  |  |  |  |  |
| 149 | *TB39.8, fhaA* | BCGM_0020c | Rv0020c | FHA domain-containing protein FhaA | 9 | 98 | LGHSEIIVR | 0.0253 | 57 | 6 | 56.0 | 4.87 | 49.0 | 4.4 |
|  |  |  | BCG0050c |  |  |  | YTESPQVPGYAPQ^a^GGGYAEPAGR | 0.0795 | 65 |  |  |  |  |  |
| 150 | *TB39.8, fhaA* | BCGM_0020c | Rv0020c | FHA domain-containing protein FhaA | 9 | 133 | LGHSEIIVR | 0.0296 | 57 | 6 | 56.0 | 4.87 | 49.0 | 4.5 |
|  |  |  | BCG0050c |  |  |  | YTESPQ^a^VPGYAPQGGGYAEPAGR | 0.0751 | 100 |  |  |  |  |  |
| 151 | *TB39.8, fhaA* | BCGM_0020c | Rv0020c | FHA domain-containing protein FhaA | 9 | 186 | LGHSEIIVR | 0.0191 | 61 | 15 | 56.0 | 4.87 | 48.0 | 4.5 |
|  |  |  | BCG0050c |  |  |  | QSGGC^d^GPSPGGGQ^a^PGYGGYGEYGR | 0.0592 | 53 |  |  |  |  |  |
|  |  |  |  |  |  |  | YTESPQVPGYAPQ^a^GGGYAEPAGR | 0.0675 | 119 |  |  |  |  |  |
|  |  |  |  |  |  |  | HPGQ^a^GDYPEQ^a^IGYPDQ^a^GGYPEQR | 0.0874 | 25 |  |  |  |  |  |
| 152 | *TB39.8, fhaA* | BCGM_0020c | Rv0020c | FHA domain-containing protein FhaA | 9 | 174 | LGHSEIIVR | 0.0259 | 56 | 15 | 56.0 | 4.87 | 47.9 | 4.6 |
|  |  |  | BCG0050c |  |  |  | ^a^QSGG^d^CGPSPGGG^c^QPGYGGYGEYGR | 0.0671 | 19 |  |  |  |  |  |
|  |  |  |  |  |  |  | QSGGC^d^GPSPGGGQ^a^PGYGGYGEYGR | 0.0743 | 47 |  |  |  |  |  |
|  |  |  |  |  |  |  | YTESPQVPGYAPQ^a^GGGYAEPAGR | 0.0805 | 120 |  |  |  |  |  |
|  |  |  |  |  |  |  | HPG^a^QGDYPEQIGYPDQGGYPEQ^a^R | 0.0873 | 18 |  |  |  |  |  |
|  | *manB* | BCGM_3270c | Rv3264c | Probable phosphomannomutase ManB | 3 | 48 | DTGADIGLAFDGDADR | 0.0263 | 48 | 3 | 49.0 | 4.68 | 47.9 | 4.6 |
|  |  |  |  |  |  |  |  |  |  |  |  |  |  |  |
|  |  |  |  |  |  |  |  |  |  |  |  |  |  |  |
|  |  |  |  |  |  |  |  |  |  |  |  |  |  |  |

Table S1 *(continued)*

| **Spot no.** | **gene** | **BCG Moreau** | **Orthologs H37Rv / Pasteur** | **Protein Identification** | **FC** | **Protein score** | **Peptide Sequence** | **Delta^&^ (Da)** | **Ion Score** | **Cov (%)** | **Theor *M*_r_  (kDa)** | **Theor pI** | **Exp *M*_r_ (kDa)** | **Exp  pI** |
| --- | --- | --- | --- | --- | --- | --- | --- | --- | --- | --- | --- | --- | --- | --- |
| 153 | *serC* | BCGM_0901c | Rv0884c | Possible phosphoserine aminotransferase | 7 | 293 | IEAIAATGR | 0.0280 | 19 | 34 | 40.2 | 4.77 | 44.2 | 5.1 |
|  |  |  | BCG0936c |  |  |  | A^a^NGIVDTEPYR | 0.0322 | 39 |  |  |  |  |  |
|  |  |  |  |  |  |  | SLHLTYGEFSAK | 0.0002 | 97 |  |  |  |  |  |
|  |  |  |  |  |  |  | A^a^NGIVDTEPYRK | 0.0244 | 31 |  |  |  |  |  |
|  |  |  |  |  |  |  | ADQLTPHLEIPTAIKPR | 0.0392 | 104 |  |  |  |  |  |
|  |  |  |  |  |  |  | SQVVGTIDFVDDVDAGTVAK | 0.0256 | 85 |  |  |  |  |  |
|  |  |  |  |  |  |  | NFASDGGL^e^WLAIMSPAALSR | 0.0434 | 21 |  |  |  |  |  |
|  |  |  |  |  |  |  | LEQLQTLTTTAAALFGTSHR | 0.0498 | 45 |  |  |  |  |  |
|  |  |  |  |  |  |  | LYSWAQERPYTTPFVTDPGLR | 0.0622 | 12 |  |  |  |  |  |
| 154 | *BCGM_1880c* | BCGM_1880c | Rv1869c | Probable reductase | 7 | 142 | SAHTVELPDGAAVR | 0.0252 | 52 | 25 | 43.6 | 4.93 | 43.6 | 5.1 |
|  |  |  | BCG1905c |  |  |  | ASPTTFVIVGGGLAGAK | -0.0152 | 36 |  |  |  |  |  |
|  |  |  |  |  |  |  | RPPIPGSDAAGVHYLR | 0.0226 | 35 |  |  |  |  |  |
|  |  |  |  |  |  |  | SLSDFTIQTSDWYR | 0.0325 | 45 |  |  |  |  |  |
|  |  |  |  |  |  |  | IILFGDEEHLPYDRPPLSK | 0.0465 | 45 |  |  |  |  |  |
|  |  |  |  |  |  |  | TSDPDIYAVGDIAAAEHPLLGTR | 0.0503 | 38 |  |  |  |  |  |
| 155 | *BCGM_1880c* | BCGM_1880c | Rv1869c | Probable reductase | 7 | 429 | LLLATGSAPR | 0.0077 | 14 | 30 | 43.6 | 4.93 | 43.5 | 5.2 |
|  |  |  | BCG1905c |  |  |  | SAHTVELPDGAAVR | 0.0135 | 81 |  |  |  |  |  |
|  |  |  |  |  |  |  | EFLSFWLDGDSR | 0.0069 | 91 |  |  |  |  |  |
|  |  |  |  |  |  |  | EFLSFW^e^LDGDSR | 0.0063 | 49 |  |  |  |  |  |
|  |  |  |  |  |  |  | ASPTTFVIVGGGLAGAK | -0.0194 | 58 |  |  |  |  |  |
|  |  |  |  |  |  |  | RPPIPGSDAAGVHYLR | 0.0070 | 48 |  |  |  |  |  |
|  |  |  |  |  |  |  | SLSDFTIQTSDWYR | 0.0132 | 73 |  |  |  |  |  |
|  |  |  |  |  |  |  | SLSDFTIQTSDW^e^YR | 0.0095 | 41 |  |  |  |  |  |
|  |  |  |  |  |  |  | IILFGDEEHLPYDRPPLSK | 0.0252 | 69 |  |  |  |  |  |
|  |  |  |  |  |  |  | TSDPDIYAVGDIAAAEHPLLGTR | 0.0239 | 101 |  |  |  |  |  |
|  | *fadA3* | BCGM_1098c | Rv1074c | Probable beta-ketoacyl CoA thiolase FadA3 | 1 | 29 | AGEGDAFISAGVETVSR | 0.0102 | 26 | 8 | 42.6 | 4.92 | 43.5 | 5.2 |
|  |  |  | BCG1132c |  |  |  | VVAVALGYDFLPGTTVNR | 0.0078 | 23 |  |  |  |  |  |
| 156 | *tuf* | BCGM_0699 | Rv0685 | Probable elongation factor TU tuf (EF-TU) | 2 | 632 | TTVTGVEMFR | 0.0209 | 11 | 48 | 43.6 | 5.28 | 44.7 | 5.6 |
|  |  |  | BCG0734 |  |  |  | LIQPVAMDEGLR | 0.0253 | 43 |  |  |  |  |  |
|  |  |  |  |  |  |  | AFDQIDNAPEER | 0.0164 | 92 |  |  |  |  |  |
|  |  |  |  |  |  |  | VLHDKFPDLNETK | 0.0047 | 34 |  |  |  |  |  |
|  |  |  |  |  |  |  | LLDQ^a^GQAGDNVGLLLR | 0.0396 | 142 |  |  |  |  |  |
|  |  |  |  |  |  |  | HYAHVDAPGHADYIK | 0.0167 | 103 |  |  |  |  |  |
|  |  |  |  |  |  |  | ^f^ELLAAQEFDEDAPVVR | 0.0413 | 47 |  |  |  |  |  |
|  |  |  |  |  |  |  | ELLAAQEFDEDAPVVR | 0.0362 | 146 |  |  |  |  |  |
|  |  |  |  |  |  |  | GITINIAHVEYQTDKR | 0.0360 | 65 |  |  |  |  |  |
|  |  |  |  |  |  |  | ADAVDDEELLELVEMEVR | 0.0476 | 79 |  |  |  |  |  |
|  |  |  |  |  |  |  | GVINVNEEVEIVGIRPSTTK | 0.0464 | 87 |  |  |  |  |  |
|  |  |  |  |  |  |  | ETDKPFLMPVEDVFTITGR | 0.0448 | 18 |  |  |  |  |  |
|  |  |  |  |  |  |  | GQVVTKPGTTTPHTEFEGQVYILSK | 0.0643 | 13 |  |  |  |  |  |
| 157 | *lpd* | BCGM_0470 | Rv0462 | Dihydrolipoamide dehydrogenase Lpd | 7 | 311 | AIGVDDYMR | 0.0503 | 13 | 27 | 49.2 | 5.53 | 50.9 | 5.8 |
|  |  |  | BCG0502 |  |  |  | WDLTASELAR | 0.0446 | 23 |  |  |  |  |  |
|  |  |  |  |  |  |  | NYGVDVTIVEFLPR | 0.0580 | 101 |  |  |  |  |  |
|  |  |  |  |  |  |  | VLQAIGFAPNVEGYGLDK | 0.0338 | 46 |  |  |  |  |  |
|  |  |  |  |  |  |  | AFGISGEVTFDYGIAYDR | 0.0602 | 31 |  |  |  |  |  |
|  |  |  |  |  |  |  | THYDVVVLGAGPGGYVAAIR | 0.0736 | 115 |  |  |  |  |  |
|  |  |  |  |  |  |  | ATF^d^C^a^QPNVASFGLTEQQAR | 0.0864 | 48 |  |  |  |  |  |
|  |  |  |  |  |  |  | LVPGTSLSANVVTYEEQILSR | 0.0871 | 84 |  |  |  |  |  |

Table S1 *(continued)*

| **Spot no.** | **gene** | **BCG Moreau** | **Orthologs H37Rv / Pasteur** | **Protein Identification** | **FC** | **Protein score** | **Peptide Sequence** | **Delta^&^ (Da)** | **Ion Score** | **Cov (%)** | **Theor *M*_r_  (kDa)** | **Theor pI** | **Exp *M*_r_ (kDa)** | **Exp  pI** |
| --- | --- | --- | --- | --- | --- | --- | --- | --- | --- | --- | --- | --- | --- | --- |
| 158 | *lpd* | BCGM_0470 | Rv0462 | Dihydrolipoamide dehydrogenase Lpd | 7 | 391 | WDLTASELAR | 0.0462 | 32 | 21 | 49.2 | 5.53 | 51.0 | 5.9 |
|  |  |  | BCG0502 |  |  |  | NYGVDVTIVEFLPR | 0.0574 | 110 |  |  |  |  |  |
|  |  |  |  |  |  |  | VLQAIGFAPNVEGYGLDK | 0.0318 | 58 |  |  |  |  |  |
|  |  |  |  |  |  |  | THYDVVVLGAGPGGYVAAIR | 0.0714 | 123 |  |  |  |  |  |
|  |  |  |  |  |  |  | ATFC^d^Q^a^PNVASFGLTEQQAR | 0.0822 | 97 |  |  |  |  |  |
|  |  |  |  |  |  |  | LVPGTSLSANVVTYEEQILSR | 0.0739 | 85 |  |  |  |  |  |
| 159 | *glyA1* | BCGM_1119 | Rv1093 | Probable serine hydroxymethiltransferase 1 | 7 | 123 | VLDFAAFR | 0.0382 | 60 | 21 | 45.0 | 6.12 | 49.2 | 6.5 |
|  |  |  | BCG1153 |  |  |  | ATALEFRPK | 0.0329 | 25 |  |  |  |  |  |
|  |  |  |  |  |  |  | IAATPEFADR | 0.0318 | 19 |  |  |  |  |  |
|  |  |  |  |  |  |  | VIIAGWSAYPR | 0.0411 | 59 |  |  |  |  |  |
|  |  |  |  |  |  |  | AFPLYDGLEEWSLVGR | 0.0416 | 17 |  |  |  |  |  |
|  |  |  |  |  |  |  | YYGGC^d^EHVDVVENLAR | 0.0537 | 47 |  |  |  |  |  |
|  |  |  |  |  |  |  | AGVSVVSGGTDVHLVLVDLR | 0.0529 | 13 |  |  |  |  |  |
| 160 | *glyA1* | BCGM_1119 | Rv1093 | Probable serine hydroxymethiltransferase 1 | 7 | 366 | VLDFAAFR | 0.0411 | 57 | 26 | 45.0 | 6.12 | 49.3 | 6.7 |
|  |  |  | BCG1153 |  |  |  | ATALEFRPK | 0.0304 | 32 |  |  |  |  |  |
|  |  |  |  |  |  |  | IAATPEFADR | 0.0382 | 40 |  |  |  |  |  |
|  |  |  |  |  |  |  | VIIAGWSAYPR | 0.0417 | 67 |  |  |  |  |  |
|  |  |  |  |  |  |  | VIIAGW^e^SAYPR | 0.0357 | 44 |  |  |  |  |  |
|  |  |  |  |  |  |  | AFPLYDGLEEWSLVGR | 0.0452 | 83 |  |  |  |  |  |
|  |  |  |  |  |  |  | AFPLYDGLEE^e^WSLVGR | 0.0398 | 24 |  |  |  |  |  |
|  |  |  |  |  |  |  | YYGGC^d^EHVDVVENLAR | 0.0518 | 77 |  |  |  |  |  |
|  |  |  |  |  |  |  | AGVSVVSGGTDVHLVLVDLR | 0.0470 | 73 |  |  |  |  |  |
|  |  |  |  |  |  |  | DSPLDGQAAEDLLHEVGITVNR | 0.0704 | 66 |  |  |  |  |  |
| 161 | *fabG4* | BCGM_0247c | Rv0242c | Probable 3-oxoacyl-[acyl-carrier protein] reductase | 1 | 80 | GIGATIAEVFAR | 0.0202 | 17 | 22 | 46.8 | 6.04 | 43.9 | 6.0 |
|  |  |  | BCG0280c |  |  |  | ADILVNNAGITR | 0.0195 | 28 |  |  |  |  |  |
|  |  |  |  |  |  |  | GLHEFFTPVLR | 0.0272 | 53 |  |  |  |  |  |
|  |  |  |  |  |  |  | LTEGLVG^a^NGSIGEGGR | 0.0220 | 37 |  |  |  |  |  |
|  |  |  |  |  |  |  | AGEPPLTGSLLIGGAGR | 0.0255 | 21 |  |  |  |  |  |
|  |  |  |  |  |  |  | VVVVGGTPEAAASTNER | 0.0204 | 26 |  |  |  |  |  |
|  |  |  |  |  |  |  | AALEKDYDLVGNNLGGR | 0.0303 | 26 |  |  |  |  |  |
| 162 | *moxR1* | BCGM_1509 | Rv1479 | Probable transcriptional regulatory protein | 9 | 257 | TLAVETFAR | 0.0273 | 20 | 25 | 40.7 | 5.96 | 41.0 | 6.5 |
|  |  |  | BCG1541 |  |  |  | SWVAFGASPR | 0.0347 | 44 |  |  |  |  |  |
|  |  |  |  |  |  |  | S^e^WVAFGASPR | 0.0333 | 36 |  |  |  |  |  |
|  |  |  |  |  |  |  | IIVGQDQLVER | 0.0288 | 42 |  |  |  |  |  |
|  |  |  |  |  |  |  | INVGYPSPEEER | 0.0381 | 53 |  |  |  |  |  |
|  |  |  |  |  |  |  | IQFTPDLVPTDIIGTR | 0.0390 | 111 |  |  |  |  |  |
|  |  |  |  |  |  |  | LVLTYDALADEISPEIVINR | 0.0405 | 86 |  |  |  |  |  |
|  |  |  |  |  |  |  | LQEIAANNFVHHALVDYVVR | 0.0506 | 13 |  |  |  |  |  |
| 163 | *BCGM_0220* | BCGM_0220 | Rv0216 | Double hotdog hydratase | 7 | 376 | LVLDFYR | 0.0456 | 60 | 28 | 35.8 | 6.49 | 36.2 | 7.0 |
|  |  |  | BCG0253 |  |  |  | VGGPYFDDLSK | 0.0186 | 55 |  |  |  |  |  |
|  |  |  |  |  |  |  | FPAVGDTLYTR | 0.0420 | 99 |  |  |  |  |  |
|  |  |  |  |  |  |  | LTLNIAATHHDWR | 0.0476 | 60 |  |  |  |  |  |
|  |  |  |  |  |  |  | SLVYAVSDSASEPDR | 0.0508 | 83 |  |  |  |  |  |
|  |  |  |  |  |  |  | LVYGGHTIGLALAQATR | 0.0605 | 81 |  |  |  |  |  |
|  |  |  |  |  |  |  | IGADAPAPAADPTAHWDGAVFR | 0.0623 | 62 |  |  |  |  |  |
|  |  |  |  |  |  |  | IGADAPAPAADPTAH^e^WDGAVFR | 0.0630 | 34 |  |  |  |  |  |
|  |  |  |  |  |  |  |  |  |  |  |  |  |  |  |

Table S1 *(continued)*

| **Spot no.** | **gene** | **BCG Moreau** | **Orthologs H37Rv / Pasteur** | **Protein Identification** | **FC** | **Protein score** | **Peptide Sequence** | **Delta^&^ (Da)** | **Ion Score** | **Cov (%)** | **Theor *M*_r_  (kDa)** | **Theor pI** | **Exp *M*_r_ (kDa)** | **Exp  pI** |
| --- | --- | --- | --- | --- | --- | --- | --- | --- | --- | --- | --- | --- | --- | --- |
| 164 | *fba* | BCGM_0369c | Rv0363c | Probable fructose-bisphosphate aldolase fba | 7 | 200 | SEIEEALR | 0.0487 | 42 | 26 | 36.5 | 5.49 | 35.8 | 5.8 |
|  |  |  | BCG0401c |  |  |  | LYTSPEDFEK | 0.0204 | 39 |  |  |  |  |  |
|  |  |  |  |  |  |  | YPVNVALHTDHC^d^PK | 0.0406 | 72 |  |  |  |  |  |
|  |  |  |  |  |  |  | LRPDILAQ^a^GQQVAAAK | 0.0712 | 54 |  |  |  |  |  |
|  |  |  |  |  |  |  | YLLAATFGNVHGVYKPGNVK | 0.0607 | 55 |  |  |  |  |  |
|  |  |  |  |  |  |  | LGLPADAKPFDFVFHGGSGSLK | 0.0629 | 39 |  |  |  |  |  |
| 165 | *espC* | BCGM_3643c | Rv3615c | ESX-1 secretion-associated protein EspC | 10 | 97 | TENLTVQPER | 0.0227 | 45 | 20 | 10.8 | 5.11 | 11.8 | 5.3 |
|  |  |  | BCG3679c |  |  |  | IYSEADEAWR | 0.0257 | 57 |  |  |  |  |  |
|  |  |  |  |  |  |  | IYSEADEAWRK | 0.0250 | 42 |  |  |  |  |  |
| 166 | *fum* | BCGM_1124c | Rv1098c | Probable fumarase fum (Fumarate Hydratase) | 7 | 252 | ALDWHTVVK | -0.0020 | 49 | 17 | 50.1 | 5.31 | 48.9 | 5.6 |
|  |  |  | BCG1158c |  |  |  | AVENFPISGR | 0.0233 | 40 |  |  |  |  |  |
|  |  |  |  |  |  |  | TAANSFEAQAAR | 0.0228 | 44 |  |  |  |  |  |
|  |  |  |  |  |  |  | C^d^IAGLTANVEHLR | 0.0258 | 47 |  |  |  |  |  |
|  |  |  |  |  |  |  | VVAVLVAQTGLSELR | 0.0283 | 93 |  |  |  |  |  |
|  |  |  |  |  |  |  | LGELAIGGTAVGTGLNAPDDFGVR | 0.0453 | 92 |  |  |  |  |  |
| 167 | *metK* | BCGM_1421 | Rv1392 | Probable s-adenosylmethionine synthetase | 7 | 282 | NVVAAGLAER | 0.0259 | 32 | 28 | 43.0 | 4.90 | 50.5 | 5.1 |
|  |  |  | BCG1453 |  |  |  | EAFADITNTVR | 0.0334 | 67 |  |  |  |  |  |
|  |  |  |  |  |  |  | IIVDTYGGWAR | 0.0356 | 55 |  |  |  |  |  |
|  |  |  |  |  |  |  | ^a^NGVLPYLRPDGK | 0.0326 | 24 |  |  |  |  |  |
|  |  |  |  |  |  |  | KIIVDTYGGWAR | 0.0399 | 83 |  |  |  |  |  |
|  |  |  |  |  |  |  | FVLGGPMGDAGLTGR | 0.0408 | 32 |  |  |  |  |  |
|  |  |  |  |  |  |  | AIGEVFDLRPGAIIR | 0.0388 | 48 |  |  |  |  |  |
|  |  |  |  |  |  |  | VLNTVLDDLAHETLDASTVR | 0.0613 | 98 |  |  |  |  |  |
|  |  |  |  |  |  |  | DLNLLRPIYAPTAAYGHFGR | 0.0661 | 15 |  |  |  |  |  |
|  | *atpD* | BCGM_1337 | Rv1310 | Probable ATP synthase beta chain AtpD | 7 | 46 | FEHWSIHR | 0.0324 | 18 | 5 | 53.1 | 4.86 | 50.5 | 5.1 |
|  |  |  | BCG1370 |  |  |  | VVDLLTPYVR | 0.0224 | 31 |  |  |  |  |  |
|  |  |  |  |  |  |  | KPPAFEELEPR | 0.0349 | 38 |  |  |  |  |  |
| 168 | *atpD* | BCGM_1337 | Rv1310 | Probable ATP synthase beta chain AtpD | 7 | 161 | FEHWSIHR | 0.0372 | 52 | 19 | 53.1 | 4.86 | 49.6 | 5.1 |
|  |  |  | BCG1370 |  |  |  | VVDLLTPYVR | 0.0274 | 27 |  |  |  |  |  |
|  |  |  |  |  |  |  | TVLIQEMINR | 0.0335 | 12 |  |  |  |  |  |
|  |  |  |  |  |  |  | TISLQPTDGLVR | 0.0242 | 13 |  |  |  |  |  |
|  |  |  |  |  |  |  | KPPAFEELEPR | 0.0344 | 50 |  |  |  |  |  |
|  |  |  |  |  |  |  | NFGGTSVFAGVGER | 0.0361 | 88 |  |  |  |  |  |
|  |  |  |  |  |  |  | FTQAGSEVSTLLGR | 0.0349 | 36 |  |  |  |  |  |
|  |  |  |  |  |  |  | DTALVFGQMDEPPGTR | 0.0393 | 22 |  |  |  |  |  |
|  | *metK* | BCGM_1421 | Rv1392 | Probable S-adenosylmethionine synthetase | 7 | 53 | EAFADITNTVR | 0.0295 | 27 | 21 | 43.0 | 4.90 | 49.6 | 5.1 |
|  |  |  | BCG1453 |  |  |  | IIVDTYGGWAR | 0.0295 | 30 |  |  |  |  |  |
|  |  |  |  |  |  |  | ^a^NGVLPYLRPDGK | 0.0350 | 13 |  |  |  |  |  |
|  |  |  |  |  |  |  | KIIVDTYGGWAR | 0.0344 | 28 |  |  |  |  |  |
|  |  |  |  |  |  |  | FVLGGPMGDAGLTGR | 0.0381 | 17 |  |  |  |  |  |
|  |  |  |  |  |  |  | AIGEVFDLRPGAIIR | 0.0272 | 10 |  |  |  |  |  |
|  |  |  |  |  |  |  | VLNTVLDDLAHETLDASTVR | 0.0562 | 35 |  |  |  |  |  |
| 169 | *glnA1* | BCGM_2221 | Rv2220 | Glutamine synthetase | 7 | 217 | SVFDDGLAFDGSSIR | 0.0366 | 98 | 23 | 53.5 | 5.04 | 54.7 | 5.4 |
|  |  |  | BCG2237 |  |  |  | LVPGYEAPINLVYSQR | 0.0516 | 87 |  |  |  |  |  |
|  |  |  |  |  |  |  | HKGGYFPVAPNDQYVDLR | 0.0598 | 58 |  |  |  |  |  |
|  |  |  |  |  |  |  | DGAPLMYDETGYAGLSDTAR | 0.0628 | 44 |  |  |  |  |  |
|  |  |  |  |  |  |  | GFQSIHESDMLLLPDPETAR | 0.0671 | 18 |  |  |  |  |  |
|  |  |  |  |  |  |  | ENEIEPVNIRPHPYEFALYYDV | 0.0924 | 11 |  |  |  |  |  |

Table S1 *(continued)*

| **Spot no.** | **gene** | **BCG Moreau** | **Orthologs H37Rv / Pasteur** | **Protein Identification** | **FC** | **Protein score** | **Peptide Sequence** | **Delta^&^ (Da)** | **Ion Score** | **Cov (%)** | **Theor *M*_r_  (kDa)** | **Theor pI** | **Exp *M*_r_ (kDa)** | **Exp  pI** |
| --- | --- | --- | --- | --- | --- | --- | --- | --- | --- | --- | --- | --- | --- | --- |
| 170 | *glnA1* | BCGM_2221 | Rv2220 | Glutamine synthetase | 7 | 262 | SVFDDGLAFDGSSIR | 0.0205 | 113 | 23 | 53.5 | 5.04 | 54.5 | 5.4 |
|  |  |  | BCG2237 |  |  |  | LVPGYEAPINLVYSQR | 0.0372 | 87 |  |  |  |  |  |
|  |  |  |  |  |  |  | HKGGYFPVAPNDQYVDLR | 0.0393 | 37 |  |  |  |  |  |
|  |  |  |  |  |  |  | DGAPLMYDETGYAGLSDTAR | 0.0362 | 91 |  |  |  |  |  |
|  |  |  |  |  |  |  | GFQSIHESDMLLLPDPETAR | 0.0429 | 26 |  |  |  |  |  |
|  |  |  |  |  |  |  | ENEIEPVNIRPHPYEFALYYDV | 0.0778 | 13 |  |  |  |  |  |
| 171 | *aldC* | BCGM_2863c | Rv2858c | Probable aldehyde dehydrogenase | 7 | 125 | FVTHPDIR | 0.0371 | 23 | 16 | 48.2 | 5.31 | 56.0 | 5.7 |
|  |  |  | BCG2880c |  |  |  | IVFTGSTEVGKR | 0.0465 | 11 |  |  |  |  |  |
|  |  |  |  |  |  |  | DVLAFYAASPER | 0.0530 | 76 |  |  |  |  |  |
|  |  |  |  |  |  |  | VAGYVPDDAPVAFR | 0.0544 | 49 |  |  |  |  |  |
|  |  |  |  |  |  |  | SANIVFHDC^d^DLER | 0.0530 | 32 |  |  |  |  |  |
|  |  |  |  |  |  |  | AVESGNLSVNSHSSVR | 0.0557 | 37 |  |  |  |  |  |
| 172 | *acn* | BCGM_1505c | Rv1475c | Probable aconitate hydratase Acn | 7 | 62 | AVIAESFER | 0.0296 | 27 | 8 | 102.4 | 4.95 | 108.7 | 5.2 |
|  |  |  | BCG1537c |  |  |  | GDGATIEFDAVVR | 0.0344 | 28 |  |  |  |  |  |
|  |  |  |  |  |  |  | NQLLDDVSGGYTR | 0.0319 | 28 |  |  |  |  |  |
|  |  |  |  |  |  |  | AEPSIEIQYTPAR | 0.0345 | 16 |  |  |  |  |  |
|  |  |  |  |  |  |  | LRN^a^QLLDDVSGGYTR | -0.0214 | 21 |  |  |  |  |  |
|  |  |  |  |  |  |  | TTIAPGSQVVNDYYDR | 0.0326 | 21 |  |  |  |  |  |
|  |  |  |  |  |  |  | FVEFYGEGVAEVPLANR | 0.0447 | 46 |  |  |  |  |  |
| 173 | *acn* | BCGM_1505c | Rv1475c | Probable aconitate hydratase Acn | 7 | 36 | FVEFYGEGVAEVPLANR | 0.0320 | 36 | 1 | 102.4 | 4.95 | 109.5 | 5.1 |
|  |  |  | BCG1537c |  |  |  |  |  |  |  |  |  |  |  |
|  |  |  |  |  |  |  |  |  |  |  |  |  |  |  |
| ^a^ Peptide with modification of asparagine or glutamine by deamidation (NQ). | | | | | | | | | | | | | |  |
| ^b^ Methionine modified by oxidation (M). | | | | | | | | | | | | | |  |
| ^c^ Peptide with modification of glutamine to pyroglutamic acid in N-terminal ð Gln->pyro-Glu (N-term Q). | | | | | | | | | | | | | |  |
| ^d^ Cysteine modified by carbamidomethyl (C). | | | | | | | | | | | | | |  |
| ^e^ Histidine or Tryptophan modified by oxidation (HW). | | | | | | | | | | | | | |  |
| ^f^ Peptide with modification of glutamate to pyroglutamic acid in N-terminal ð Glu->pyro-Glu (N-term E). | | | | | | | | | | | | | |  |
| ^g^ Peptide with acetylation on N-terminal threonine | | | | | | | | | | | | | |  |
| ^#^ Proteins identified only in *M. bovis* BCG Moreau. | | | | | | | | | | | | | |  |
| ^&^ Difference (error) between the experimental and calculated relative peptide molecular masses. | | | | | | | | | | | | | |  |
| *Functional classification (FC) according to Tuberculist (TubercuList, 2004) | | | | | | | | | | | | | |  |
| $ Threshold scores ≥ 0.5 unless a signal peptide is predicted at the same time | | | | | | | | | | | | | |  |
| NA means Not Applicable | | | | | | | | | | | | | |  |

**Reference:**

TubercuList (2004). *TubercuList World-Wide Web Server* [Online]. Available: <http://genolist.pasteur.fr/TubercuList/> [Accessed].

.

**Table S2. Predicted localization of identified proteins.** Signal P (sec-dependent secretion), LipoP (lipoprotein signal peptide), TatP (twin-arginine translocation) and SecretomeP (non-classical secretion) tools were used for prediction of protein localization as well as number of potential transmembrane domais predicted with TMHMM. PRED-TMBB tool was used to predict beta-barrel outer membrane proteins. Proteins identified in our study are compared to those previously reported in the culture filtrate of BCG Moreau (Moreau CF,(Berredo-Pinho et al., 2011) or as present in the culture filtrate or membrane (memb) fractions of *M. tuberculosis* H37Rv, as listed in Tuberculist (TubercuList, 2004).

Table S2

| **BCG Moreau** | **gene** | **Protein Identification** | **Signal P** | **Lipo P** | **Tat P** | **Sec P** | **TMHMM** | **PRED-TMBB** | **Moreau CF** | **Tuberculist** |
| --- | --- | --- | --- | --- | --- | --- | --- | --- | --- | --- |
| BCGM_0009 | *ppiA* | Probable iron-regulated peptidyl-prolyl cis-trans isomerase A | - | - | - | yes | 0 | - | yes | yes |
| BCGM_0020c | *TB39.8, fhaA* | FHA domain-containing protein FhaA | - | - | - | yes | 0 | - | yes | yes |
| BCGM_0047c | *ino1* | Myo-inositol-1-phosphate synthase | - | - | - | - | 0 | - | no | yes |
| BCGM_0048c | *BCGM_0048c* | Conserved hypothetical protein | - | - | - | - | 0 | yes | no | memb |
| BCGM_0055 | *ssb* | Probable single-strand binding protein | - | - | - | yes | 0 | yes | yes | yes |
| BCGM_0101 | *BCGM_0101* | Possible oxidoreductase | - | - | - | - | 0 | - | no | no |
| BCGM_0129 | *pepA* | Probable serine protease | 32-33 | - | - | - | 1 | yes | yes | yes |
| BCGM_0152 | *BCGM_0152* | Probable short-chain type dehydrogenase/reductase | - | - | - | - | 0 | yes | yes | yes |
| BCGM_0220 | *BCGM_0220* | Double hotdog hydratase | - | - | - | - | 0 | yes | yes | yes |
| BCGM_0238c | *gabD1* | Probable succinate-semialdehyde dehydrogenase [nadp+] dependent | - | - | - | - | 0 | yes | yes | yes |
| BCGM_0247c | *fabG4* | Probable 3-oxoacyl-[acyl-carrier protein] reductase | - | - | - | - | 0 | yes | no | yes |
| BCGM_0289 | *BCGM_0289* | Putative S-adenosyl-L-methionine-dependent methyltransferase | - | - | - | - | 0 | yes | no | yes |
| BCGM_0340 | *BCGM_0340* | Hypothetical protein | - | - | - | - | 0 | - | no | memb |
| BCGM_0357 | *dnaK* | Probable chaperone protein | - | - | - | - | 0 | yes | yes | yes |
| BCGM_0358 | *grpE* | Probable grpE protein (hsp-70 cofactor) | - | - | - | yes | 0 | yes | no | yes |
| BCGM_0369c | *fba* | Probable fructose-biphosphate aldolase | - | - | - | - | 0 | - | yes | yes |
| BCGM_0447 | *groEL2* | 60 kDa chaperonin 2, GroEL2 | - | - | - | - | 0 | yes | yes | yes |
| BCGM_0470 | *lpd* | Dihydrolipoamide dehydrogenase Lpd | - | - | - | - | 0 | yes | yes | yes |
| BCGM_0475 | *icl* | Isocitrate lyase | - | - | - | - | 0 | - | yes | yes |
| BCGM_0498 | *gpm1* | Probable phosphoglycerate mutase 1 | - | - | - | - | 0 | yes | no | yes |
| BCGM_0588 | *TB27.3, cfp32* | Putative glyoxylase CFP32 | - | - | - | yes | 0 | yes | yes | yes |
| BCGM_0644c | *echA3* | Probable enoyl-CoA hydratase | - | - | - | - | 0 | yes | yes | yes |
| BCGM_0649 | *hadA* | (3R)-hydroxyacyl-ACP dehydratase subunit HadA | - | - | - | - | 0 | - | no | memb |
| BCGM_0650 | *hadB* | (3R)-hydroxyacyl-ACP dehydratase subunit HadB | - | - | - | yes | 0 | yes | no | yes |
| BCGM_0651 | *hadC* | (3R)-hydroxyacyl-ACP dehydratase subunit HadC | - | - | - | - | 0 | - | no | memb |
| BCGM_0653 | *nusG* | Probable transcription antitermination protein NusG | - | - | - | yes | 0 | yes | no | yes |
| BCGM_0699 | *tuf* | Probable elongation factor TU tuf (EF-TU) | - | - | - | - | 0 | - | yes | yes |
| BCGM_0818 | *Rv0801* | Conserved hypothetical protein | - | - | - | - | 0 | - | no | memb |
| BCGM_0830c | *BCGM_0830c* | UPF0678 fatty acid-binding protein-like protein | - | - | - | - | 0 | yes | no | memb |
| BCGM_0831c | *sseC2* | Conserved hypothetical protein SseC2 | - | - | - | yes | 0 | yes | no | memb |

Table S2: continued

| **BCG Moreau** | **gene** | **Protein Identification** | **Signal P** | **Lipo P** | **Tat P** | **Sec P** | **TMHMM** | **PRED-TMBB** | **Moreau CF** | **Tuberculist** |
| --- | --- | --- | --- | --- | --- | --- | --- | --- | --- | --- |
| BCGM_0832c | *cysA2* | Probable thiosulfate sulfurtransferase | - | - | - | yes | 0 | - | yes | yes |
| BCGM_0871 | *BCGM_0871* | Conserved hypothetical protein | - | - | - | yes | 0 | - | no | memb |
| BCGM_0876 | *fadA* | Possible acyl-CoA thiolase | - | - | - | - | 0 | yes | no | memb |
| BCGM_0901c | *serC* | Possible phosphoserine aminotransferase | - | - | - | yes | 0 | yes | yes | yes |
| BCGM_0945 | *pstS3* | Periplasmic phosphate-binding lipoprotein PstS3 | - | 22-23 | - | yes | 0 | yes | yes | yes |
| BCGM_1046 | *eno* | Probable enolase | - | - | - | - | 0 | yes | no | yes |
| BCGM_1062c | *esxJ* | ESAT-6 like protein EsxJ (ESAT-6 like protein 2) | - | - | - | - | 0 | - | yes | yes |
| BCGM_1098c | *fadA3* | Probable beta-ketoacyl CoA thiolase | - | - | - | - | 0 | yes | yes | yes |
| BCGM_1119 | *glyA1* | Probable serine hydroxymethiltransferase 1 | - | - | - | - | 0 | yes | yes | yes |
| BCGM_1124c | *fum* | Probable fumarase fum (Fumarate hydratase) | - | - | - | - | 0 | yes | yes | yes |
| BCGM_1125c | *glpX* | Fructose-1,6-bisphosphatase class 2 (FBPase) | - | - | - | - | 0 | yes | no | memb |
| BCGM_1160c | *metE* | 5-methyltetrahydropteroyltriglutamate-homocysteine methyltransferase | - | - | - | - | 0 | yes | yes | yes |
| BCGM_1187c | *BCGM_1187c* | Pterin-4-alpha-carbinolamine dehydratase | - | - | - | - | 0 | - | no | yes |
| BCGM_1267 | *mdh* | Probable malate dehydrogenase | - | - | - | - | 0 | yes | no | memb |
| BCGM_1310 | *canA* | Beta-carbonic anhydrase | - | - | - | - | 0 | - | no | yes |
| BCGM_1322 | *thrC* | Probable threonine synthase | - | - | - | yes | 0 | yes | no | yes |
| BCGM_1337 | *atpD* | Probable ATP synthase beta chain atpD | - | - | - | - | 0 | - | no | yes |
| BCGM_1368 | *BCGM_1368* | Conserved hypothetical protein | - | - | - | - | 0 | - | no | memb |
| BCGM_1421 | *metK* | Probable s-adenosylmethionine synthetase | - | - | - | - | 0 | yes | no | memb |
| BCGM_1465 | *gap* | Probable glyceraldehyde 3-phosphate dehydrogenase | - | - | - | - | 0 | yes | yes | yes |
| BCGM_1466 | *pgk* | Probable phosphoglycerate kinase | - | - | - | - | 0 | yes | no | yes |
| BCGM_1494 | *BCGM_1494* | Possible nitrogen fixation related protein | - | - | - | - | 0 | - | no | memb |
| BCGM_1505c | *acn* | Probable aconitate hydratase Acn | - | - | - | yes | 0 | - | yes | yes |
| BCGM_1509 | *moxR1* | Probable transcriptional regulatory protein | - | - | - | - | 0 | yes | no | yes |
| BCGM_1641 | *TB15.3* | Iron-regulated universal stress protein family protein TB15.3 | - | - | - | - | 0 | - | yes | yes |
| BCGM_1659 | *argJ* | Probable glutamate n-acetyltransferase | - | - | - | - | 0 | yes | no | memb |
| BCGM_1664 | *argG* | Probable Argininosuccinate synthase | - | - | - | - | 0 | - | no | memb |
| BCGM_1738c | *BCGM_1738c* | Conserved hypothetical protein | - | - | - | yes | 0 | - | yes | yes |
| BCGM_1802 | *BCGM_1802* | Conserved hypothetical protein | - | - | - | - | 0 | yes | no | yes |
| BCGM_1838 | *cfp17, garA* | Glycogen accumulation regulator GarA | - | - | - | yes | 0 | yes | yes | yes |

Table S2: continued

| **BCG Moreau** | **gene** | **Protein Identification** | **Signal P** | **Lipo P** | **Tat P** | **Sec P** | **TMHMM** | **PRED-TMBB** | **Moreau CF** | **Tuberculist** |
| --- | --- | --- | --- | --- | --- | --- | --- | --- | --- | --- |
| BCGM_1871 | *apa* | Alanine and proline rich secreted protein | 39-40 | - | - | - | 1 | yes | yes | yes |
| BCGM_1880c | *BCGM_1880c* | Probable reductase | - | - | - | - | 0 | yes | yes | yes |
| BCGM_1885 | *BCGM_1885* | Hypothetical protein | - | - | - | - | 0 | - | no | memb |
| BCGM_1898c | *fbpB* | Secreted antigen 85-B | - | - | 40-41 | yes | 1 | yes | yes | yes |
| BCGM_1910c | *BCGM_1910c* | Putative S-adenosyl-L-met-dependent methyltransferase | - | - | - | yes | 0 | - | no | no |
| BCGM_1939c | *mpt63* | Immunogenic protein Mpt63 | 29-30 | - | - | - | 1 | yes | yes | yes |
| BCGM_1945 | *tpx* | Probable thiol peroxidase | - | - | 26-27 | - | 0 | yes | no | yes |
| BCGM_1981c | *mpb64* | Immunogenic protein Mpt64 (antigen Mpt64/Mpb64) | 23-24 | - | - | - | 1 | - | yes | yes |
| BCGM_2035c | *hspX* | Heat shock protein | - | - | - | - | 0 | - | yes | yes |
| BCGM_2100c | *pafB* | Proteasome accessory factor B PafB | - | - | - | - | 0 | yes | no | memb |
| BCGM_2111c | *prcB* | Proteasome (beta subunit) | - | - | - | - | 0 | yes | yes | yes |
| BCGM_2141c | *TB18.6* | Conserved hypothetical protein TB18.6 | - | - | - | yes | 0 | yes | yes | yes |
| BCGM_2202c | *adoK* | Adenosine kinase | - | - | - | - | 0 | yes | no | yes |
| BCGM_2215 | *dlaT* | Dihydrolipoamide acyltransferase | - | - | - | yes | 0 | yes | no | memb |
| BCGM_2221 | *glnA1* | Glutamine synthetase | - | - | - | yes | 0 | - | yes | yes |
| BCGM_2239c | *ahpE* | Peroxiredoxin AhpE | - | - | - | - | 0 | - | no | yes |
| BCGM_2247 | *kasB* | 3-oxoacyl-[acyl-carrier protein] synthase 2 | - | - | - | - | 0 | yes | no | yes |
| BCGM_2354c | *PPE71* | PPE family protein | - | - | 36-37 | yes | 0 | yes | no | memb |
| BCGM_2431 | *ahpC* | Alkyl hydroperoxide reductase c protein | - | - | - | - | 0 | - | no | memb |
| BCGM_2449c | *ndkA* | Probable nucleoside diphosphate kinase ndkA | - | - | - | - | 0 | yes | yes | yes |
| BCGM_2464c | *clpP2* | Probable ATP-dependent clp protease proteolytic subunit 2 | - | - | - | - | 0 | yes | no | memb |
| BCGM_2466c | *tig* | Probable trigger factor (tf) protein | - | - | - | - | 0 | yes | yes | yes |
| BCGM_2469c | *rpiB* | Ribose-5-phosphate isomerase | - | - | - | - | 0 | - | no | yes |
| BCGM_2636c | *hrp1* | Hypoxic response protein 1 Hrp1 | - | - | - | - | 0 | - | yes | yes |
| BCGM_2748 | *BCGM_2748* | Conserved hypothetical protein | - | - | - | - | 0 | - | no | yes |
| BCGM_2863c | *aldC* | Probable aldehyde dehydrogenase | - | - | - | - | 0 | yes | no | yes |
| BCGM_2878 | *mpb83* | Cell surface lipoprotein Mpb83 | - | 24-25 | - | yes | 0 | yes | yes | yes |
| BCGM_2880 | *mpb70* | Major secreted immunogenic protein mpb70 | 30-31 | - | - | - | 1 | yes | yes | yes |
| BCGM_2886c | *frr* | Ribosome recycling factor | - | - | - | - | 0 | - | yes | yes |
| BCGM_2893c | *tsf* | Probable elongation factor TSF | - | - | - | - | 0 | yes | yes | yes |

Table S2: continued

| **BCG Moreau** | **gene** | **Protein Identification** | **Signal P** | **Lipo P** | **Tat P** | **Sec P** | **TMHMM** | **PRED-TMBB** | **Moreau CF** | **Tuberculist** |
| --- | --- | --- | --- | --- | --- | --- | --- | --- | --- | --- |
| BCGM_2923c | *glnB* | Probable nitrogen regulatory protein P-II | - | - | - | - | 0 | - | no | memb |
| BCGM_2928c | *BCGM_2928c* | Conserved hypothetical protein | - | - | - | - | 0 | yes | no | memb |
| BCGM_2977 | *BCGM_2977* | Probable oxidoreductase | - | - | - | - | 0 | yes | yes | yes |
| BCGM_3019 | *BCGM_3019* | Conserved hypothetical protein | - | - | - | - | 0 | yes | no | memb |
| BCGM_3035c | *fixB* | Probable electron transfer flavoprotein (alpha-subunit) | - | - | - | - | 0 | yes | no | yes |
| BCGM_3036c | *fixA* | Probable electron transfer flavoprotein (beta-subunit) | - | - | - | - | 0 | yes | yes | yes |
| BCGM_3053 | *adhC* | Probable NADP-dependent alcohol dehydrogenase | - | - | - | - | 0 | yes | yes | yes |
| BCGM_3219c | *TB9.4* | Conserved hypothetical protein TB9.4 | - | - | - | - | 0 | yes | yes | yes |
| BCGM_3268c | *manA* | Probable mannose-6-phosphate isomerase | - | - | - | - | 0 | yes | no | yes |
| BCGM_3270c | *manB* | Probable phosphomannomutase ManB | - | - | - | - | 0 | yes | no | memb |
| BCGM_3287c | *fadE25* | Probable acyl-CoA dehydrogenase | - | - | - | - | 0 | yes | no | yes |
| BCGM_3320 | *deoD_1* | Probable purine nucleoside phosphorylase DeoD (inosine phosphorylase) | - | - | - | - | 0 | yes | no | memb |
| BCGM_3375c | *trpS* | Probable tryptophanyl-tRNA synthetase TrpS | - | - | - | - | 0 | - | no | yes |
| BCGM_3426c | *htdY* | Probable 3-hydroxyacyl-thioester dehydratase HtdY | - | - | - | - | 0 | yes | yes | yes |
| BCGM_3440 | *BCGM_3440* | Alpha-ketoglutarate-dependent sulfate ester dioxygenase | - | - | - | - | 0 | - | no | memb |
| BCGM_3442 | *vapC47* | Ribonuclease VapC47 | - | - | - | - | 0 | yes | no | no |
| BCGM_3451c | *groEL1* | 60 kDa chaperonin 1, GroEL1 | - | - | - | - | 0 | yes | no | memb |
| BCGM_3452c | *groES* | 10 kDa chaperonin | - | - | - | yes | 0 | yes | yes | yes |
| BCGM_3578 | *echA20* | Probable enoyl-coa hydratase | - | - | - | - | 0 | yes | no | memb |
| BCGM_3643c | *espC* | ESX-1 secretion-associated protein EspC | - | - | - | - | 0 | yes | no | memb |
| BCGM_3723 | *BCGM_3723* | Conserved hypothetical protein | - | - | - | yes | 0 | - | yes | yes |
| BCGM_3741c | *BCGM_3741c* | Nucleoid-associated protein Rv3716c | - | - | - | yes | 0 | - | no | yes |
| BCGM_3778c | *BCGM_3778c* | Conserved hypothetical protein | - | - | - | - | 0 | yes | no | yes |
| BCGM_3830c | *fbpA* | Secreted antigen 85-A | - | - | 41-42 | yes | 1 | yes | yes | yes |
| BCGM_3880 | *menG* | S-adenosylmethionine:2-demethylmenaquinone methyltransferase | - | - | - | - | 0 | yes | no | yes |

**References:**

- Berredo-Pinho, M., Kalume, D.E., Correa, P.R., Gomes, L.H., Pereira, M.P., da Silva, R.F., et al. (2011). Proteomic profile of culture filtrate from the Brazilian vaccine strain Mycobacterium bovis BCG Moreau compared to M. bovis BCG Pasteur. *BMC Microbiol* 11**,** 80. doi: 10.1186/1471-2180-11-80.

- TubercuList (2004). *TubercuList World-Wide Web Server* [Online]. Available: <http://genolist.pasteur.fr/TubercuList/> [Accessed].

**Table S3: Surface-associated proteins with moonlighting predicted function differentially expressed between strains Moreau and Pasteur**

Table S3

| **Spot no.** | **gene** | **Moreau vs Pasteur*** | **Orthologs Moreau / H37Rv / Pasteur** | **EC number** | **Predicted Function** | **Moonlighting Function** | **Organism/Species** | **References** |
| --- | --- | --- | --- | --- | --- | --- | --- | --- |
| ­67 | *ppiA* | ⇧ | BCGM_0009 | 5.2.1.8 | Probable iron-regulated peptidyl-prolyl cis-trans isomerase A | Proinflammatory cytokine - activate endothelial cells | *Homo sapiens* | (Jin et al., 2004) |
|  |  |  | Rv0009 |  |  | Induces apoptosis of gastric epithelial cells | *Helicobacter pylori* | (Basak et al., 2005) |
|  |  |  | BCG0009 |  |  | Ppiases might play a role in the intracellular survival by subverting the host cell defenses, such as oxidative stress as well as by immunomodulation | *Mycobacterium tuberculosis* | (Pandey et al., 2017) |
| ­4 | *dnaK* | ⇧ | BCGM_0357 | 3.6.1.- | Probable chaperone protein | Plasminogen binding | *Bifidobacterium* | (Candela et al., 2007) |
|  |  |  | Rv0350 |  |  |  | *Neisseria meningitidis* | (Knaust et al., 2007) |
|  |  |  | BCG0389 |  |  |  |  |  |
| 66 | *Fba* | ⇩ | BCGM_0369c | 4.1.2.13 | Probable fructose-biphosphate aldolase | Plasminogen binding | *Candida albicans* | (Crowe et al., 2003) |
|  |  |  | Rv0363c |  |  | V-ATPase assembly | *Saccharomyces cerevisiae* | (Lu et al., 2001); (Lu et al., 2004); (Lu et al., 2007) |
|  |  |  | BCG0401c |  |  | Adhesin | *Streptococcus pneumoniae; Neisseria meningitidis* | (Blau et al., 2007); (Tunio et al., 2010) |
| 5 | *groEL2* | ⇩ | BCGM_0447 | NA** | 60 kDa chaperonin 2, GroEL2 | Toxin | *Enterobacter aerogenes* | (Yoshida et al., 2001) |
|  |  |  | Rv0440 |  |  | Invasion in a HeLa cell model | *Legionella pneumophila* | (Garduno et al., 1998) |
|  |  |  | BCG0479 |  |  | Binds DNA | *Mycobacterium tuberculosis* | (Basu et al., 2009) |
|  |  |  |  |  |  | Regulation of inflamation | *Mycobacterium leprae* | (Rha et al., 2002) |
|  |  |  |  |  |  | Aggravation of atherosclerosis | *Chlamydia pneumoniae* | (Kol et al., 1998) |
|  |  |  |  |  |  | Role in the growth of the bacteria | *Helicobacter pylori* | (Yamaguchi et al., 1997) |
|  |  |  |  |  |  | Essential for biofilm formation | *Mycobacterium smegmatis* | (Ojha et al., 2005) |
|  |  |  |  |  |  | Stimulation of osteoclastogenesis | *Escherichia coli* | (Reddi et al., 1998) |
| 122 | *gpm1* | ⇩ | BCGM_0498 | 5.4.2.1 | Probable phosphoglycerate mutase 1 | Plasminogen binding protein | *Streptococcus sp. oral* | (Kinnby et al., 2008) |
|  |  |  | Rv0489 |  |  |  |  |  |
|  |  |  | BCG0530 |  |  |  |  |  |
| 118 | *echA3* | ⇧ | BCGM_0644c | 4.2.1.17 | Probable enoyl-CoA hydratase | RNA and microtubule binding protein | Rice seed | (Chuong et al., 2002) |
|  |  |  | Rv0632c |  |  |  |  |  |
|  |  |  | BCG0679c |  |  |  |  |  |
| ­154 | *BCGM_1880c* | ⇧ | BCGM_1880c | 1.-.-.- | Probable reductase | Protease | Eukaryotes | (Babady et al., 2007) |
|  |  |  | Rv1869c |  |  |  |  |  |
|  |  |  | BCG1905c |  |  |  |  |  |
| 121 | *ahpC* | ⇧ | BCGM_2431 | 1.-.-.- | Alkyl hydroperoxide reductase c protein | Molecular chaperone function | Yeast | (Jang et al., 2004) |
|  |  |  | Rv2428 |  |  | Plasminogen binding | *Candida albicans* | (Crowe et al., 2003) |
|  |  |  | BCG2447 |  |  | Cell wall biogenesis | *Candida albicans* | (Urban et al., 2005) |
|  |  |  |  |  |  | Bifunctional enzyme with Glutathione peroxidase and phospholipase A2 activities | *Homo sapiens* | (Chen et al., 2000) |

* ⇧ is more expressed in Moreau than Pasteur strain

⇩ is more expressed in Pasteur than Moreau strain

** NA means Not Applicable

**References**

Babady, N.E., Pang, Y.P., Elpeleg, O., and Isaya, G. (2007). Cryptic proteolytic activity of dihydrolipoamide dehydrogenase. *Proc Natl Acad Sci U S A* 104(15)**,** 6158-6163. doi: 10.1073/pnas.0610618104.

Basak, C., Pathak, S.K., Bhattacharyya, A., Pathak, S., Basu, J., and Kundu, M. (2005). The secreted peptidyl prolyl cis,trans-isomerase HP0175 of Helicobacter pylori induces apoptosis of gastric epithelial cells in a TLR4- and apoptosis signal-regulating kinase 1-dependent manner. *J Immunol* 174(9)**,** 5672-5680.

Basu, D., Khare, G., Singh, S., Tyagi, A., Khosla, S., and Mande, S.C. (2009). A novel nucleoid-associated protein of Mycobacterium tuberculosis is a sequence homolog of GroEL. *Nucleic Acids Res* 37(15)**,** 4944-4954. doi: 10.1093/nar/gkp502.

Blau, K., Portnoi, M., Shagan, M., Kaganovich, A., Rom, S., Kafka, D., et al. (2007). Flamingo cadherin: a putative host receptor for Streptococcus pneumoniae. *J Infect Dis* 195(12)**,** 1828-1837. doi: 10.1086/518038.

Candela, M., Bergmann, S., Vici, M., Vitali, B., Turroni, S., Eikmanns, B.J., et al. (2007). Binding of human plasminogen to Bifidobacterium. *J Bacteriol* 189(16)**,** 5929-5936. doi: 10.1128/JB.00159-07.

Chen, J.W., Dodia, C., Feinstein, S.I., Jain, M.K., and Fisher, A.B. (2000). 1-Cys peroxiredoxin, a bifunctional enzyme with glutathione peroxidase and phospholipase A2 activities. *J Biol Chem* 275(37)**,** 28421-28427. doi: 10.1074/jbc.M005073200.

Chuong, S.D., Mullen, R.T., and Muench, D.G. (2002). Identification of a rice RNA- and microtubule-binding protein as the multifunctional protein, a peroxisomal enzyme involved in the beta -oxidation of fatty acids. *J Biol Chem* 277(4)**,** 2419-2429. doi: 10.1074/jbc.M109510200.

Crowe, J.D., Sievwright, I.K., Auld, G.C., Moore, N.R., Gow, N.A., and Booth, N.A. (2003). Candida albicans binds human plasminogen: identification of eight plasminogen-binding proteins. *Mol Microbiol* 47(6)**,** 1637-1651.

Garduno, R.A., Garduno, E., and Hoffman, P.S. (1998). Surface-associated hsp60 chaperonin of Legionella pneumophila mediates invasion in a HeLa cell model. *Infect Immun* 66(10)**,** 4602-4610.

Jang, H.H., Lee, K.O., Chi, Y.H., Jung, B.G., Park, S.K., Park, J.H., et al. (2004). Two enzymes in one; two yeast peroxiredoxins display oxidative stress-dependent switching from a peroxidase to a molecular chaperone function. *Cell* 117(5)**,** 625-635. doi: 10.1016/j.cell.2004.05.002.

Jin, Z.G., Lungu, A.O., Xie, L., Wang, M., Wong, C., and Berk, B.C. (2004). Cyclophilin A is a proinflammatory cytokine that activates endothelial cells. *Arterioscler Thromb Vasc Biol* 24(7)**,** 1186-1191. doi: 10.1161/01.ATV.0000130664.51010.28.

Kinnby, B., Booth, N.A., and Svensater, G. (2008). Plasminogen binding by oral streptococci from dental plaque and inflammatory lesions. *Microbiology* 154(Pt 3)**,** 924-931. doi: 10.1099/mic.0.2007/013235-0.

Knaust, A., Weber, M.V., Hammerschmidt, S., Bergmann, S., Frosch, M., and Kurzai, O. (2007). Cytosolic proteins contribute to surface plasminogen recruitment of Neisseria meningitidis. *J Bacteriol* 189(8)**,** 3246-3255. doi: 10.1128/JB.01966-06.

Kol, A., Sukhova, G.K., Lichtman, A.H., and Libby, P. (1998). Chlamydial heat shock protein 60 localizes in human atheroma and regulates macrophage tumor necrosis factor-alpha and matrix metalloproteinase expression. *Circulation* 98(4)**,** 300-307.

Lu, M., Ammar, D., Ives, H., Albrecht, F., and Gluck, S.L. (2007). Physical interaction between aldolase and vacuolar H+-ATPase is essential for the assembly and activity of the proton pump. *J Biol Chem* 282(34)**,** 24495-24503. doi: 10.1074/jbc.M702598200.

Lu, M., Holliday, L.S., Zhang, L., Dunn, W.A., Jr., and Gluck, S.L. (2001). Interaction between aldolase and vacuolar H+-ATPase: evidence for direct coupling of glycolysis to the ATP-hydrolyzing proton pump. *J Biol Chem* 276(32)**,** 30407-30413. doi: 10.1074/jbc.M008768200.

Lu, M., Sautin, Y.Y., Holliday, L.S., and Gluck, S.L. (2004). The glycolytic enzyme aldolase mediates assembly, expression, and activity of vacuolar H+-ATPase. *J Biol Chem* 279(10)**,** 8732-8739. doi: 10.1074/jbc.M303871200.

Ojha, A., Anand, M., Bhatt, A., Kremer, L., Jacobs, W.R., Jr., and Hatfull, G.F. (2005). GroEL1: a dedicated chaperone involved in mycolic acid biosynthesis during biofilm formation in mycobacteria. *Cell* 123(5)**,** 861-873. doi: 10.1016/j.cell.2005.09.012.

Pandey, S., Tripathi, D., Khubaib, M., Kumar, A., Sheikh, J.A., Sumanlatha, G., et al. (2017). Mycobacterium tuberculosis Peptidyl-Prolyl Isomerases Are Immunogenic, Alter Cytokine Profile and Aid in Intracellular Survival. *Front Cell Infect Microbiol* 7**,** 38. doi: 10.3389/fcimb.2017.00038.

Reddi, K., Meghji, S., Nair, S.P., Arnett, T.R., Miller, A.D., Preuss, M., et al. (1998). The Escherichia coli chaperonin 60 (groEL) is a potent stimulator of osteoclast formation. *J Bone Miner Res* 13(8)**,** 1260-1266. doi: 10.1359/jbmr.1998.13.8.1260.

Rha, Y.H., Taube, C., Haczku, A., Joetham, A., Takeda, K., Duez, C., et al. (2002). Effect of microbial heat shock proteins on airway inflammation and hyperresponsiveness. *J Immunol* 169(9)**,** 5300-5307.

Tunio, S.A., Oldfield, N.J., Berry, A., Ala'Aldeen, D.A., Wooldridge, K.G., and Turner, D.P. (2010). The moonlighting protein fructose-1, 6-bisphosphate aldolase of Neisseria meningitidis: surface localization and role in host cell adhesion. *Mol Microbiol* 76(3)**,** 605-615. doi: 10.1111/j.1365-2958.2010.07098.x.

Urban, C., Xiong, X., Sohn, K., Schroppel, K., Brunner, H., and Rupp, S. (2005). The moonlighting protein Tsa1p is implicated in oxidative stress response and in cell wall biogenesis in Candida albicans. *Mol Microbiol* 57(5)**,** 1318-1341. doi: 10.1111/j.1365-2958.2005.04771.x.

Yamaguchi, H., Osaki, T., Taguchi, H., Hanawa, T., Yamamoto, T., Fukuda, M., et al. (1997). Growth inhibition of Helicobacter pylori by monoclonal antibody to heat-shock protein 60. *Microbiol Immunol* 41(12)**,** 909-916.

Yoshida, N., Oeda, K., Watanabe, E., Mikami, T., Fukita, Y., Nishimura, K., et al. (2001). Protein function. Chaperonin turned insect toxin. *Nature* 411(6833)**,** 44. doi: 10.1038/35075148.
